# A new platform for achieving long-term air-stable long persistent luminescence in amorphous organic system

**DOI:** 10.1038/s41377-026-02367-6

**Published:** 2026-08-18

**Authors:** Zhenjiang Liu, Yunshu Meng, Zhihe Chi, Jia Ren, Ziang Song, Hui Zhang, Yu Tian, Yunsheng Wang, Jiaqiang Wang, Dan Li, Manman Fang, Zheng Zhao, Jie Yang, Ben Zhong Tang, Zhen Li

**Affiliations:** 1 Institute of Molecular Aggregation Science, Tianjin University, Tianjin, 300072 China; 2 The International Joint Institute of Tianjin University, Fuzhou, Tianjin University, Tianjin, 300072 China; 3 Guangdong Basic Research Center of Excellence for Aggregate Science, School of Science and Engineering, The Chinese University of Hong Kong, Shenzhen, Guangdong 518172 China; 4 Hubei Key Lab on Organic and Polymeric Opto-Electronic Materials, Department of Chemistry, Wuhan University, Wuhan, 430072 China

**Keywords:** Optical materials and structures, Optical physics

## Abstract

Organic long persistent luminescence (OLPL) materials are at the forefront of research, undergoing rapid evolution due to their extensive potential applications. The realization of hour-level OLPL, particularly an air-stable one, remains a significant challenge. Here, we developed a new platform to achieve long-term air-stable OLPL in an amorphous system through introducing flexible phosphorescence donors as hosts and electron acceptors with the ability to form stable radical anions as guests. Based on this platform, the regulations of OLPL duration times (1.6–7154.3 s) and emission colors (500–680 nm) were easily realized by enhancing the electron-withdrawing ability and increasing the doping concentration of guest molecules, respectively. Additionally, two kinds of non-aromatic chiral substituents were introduced into the phosphorescence hosts, and a rare phenomenon of air-stable circularly polarized organic long persistent luminescence (CP-OLPL) was successfully obtained.

## Abstract (web summary)

An amorphous host–guest platform enables air-stable organic long persistent luminescence lasting for over 2 h, with tunable lifetime, color, and circular polarization characteristics.

## Graphical abstract

**Figure:**
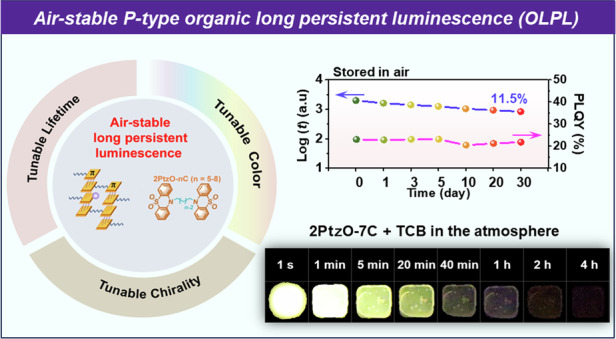

## Introduction

Organic long persistent luminescence (OLPL) is the phenomenon where the emission can last for hours or even longer after light excitation. Due to long emission lifetime, OLPL materials have great potential in practical applications like time-resolved bio-imaging and energy storage^1–8^. However, OLPL is hard to achieve because of the highly localized and unstable excitons in purely organic systems. The breakthrough was done by Adachi’s group in 2017^9^. They successfully utilized electron donor as a guest and an acceptor as a host to construct an n-type amorphous doping system with OLPL lasting for more than 1 h under nitrogen conditions, in which the photo-induced radical ion pair and the resultant charge-separated state were considered to play a key role in prolonging the exciton lifetime. Nevertheless, the performance degradation of OLPL was significantly pronounced when exposed to air, mainly because of the quenching effect from oxygen molecules (Chart S1), regardless of the efforts paid by utilizing filming technology^10^, color tuning^11^, energy level regulation of donor and acceptor^12–16^, and even polymer engineering^6^, etc. (Chart S2–S3). Generally, the oxygen-quenching effect in OLPL systems was mainly carried out in two aspects, namely photoreaction with oxygen and energy transfer to oxygen. To inhibit the photoreaction quenching, Adachi and co-workers constructed a series of ternary p-type doping systems with acceptor dopant, donor host and hole trap^17^. Under air, the corresponding amorphous films could indeed exhibit OLPL emission, but the durations would be shortened by an order of magnitude in comparison with those in nitrogen (Chart S4), because the interaction and energy transfer between long-lived triplet excitons and oxygen were hard to avoid in amorphous systems. These valuable results demonstrated the possibility of realizing air-stable OLPL on one hand, and pointed out that developing hosts capable of isolating oxygen should be an effective strategy for achieving air-stable OLPL.

In comparison with amorphous systems, crystallization could restrict the oxygen diffusion through close molecular packing more easily, thus possibly promoting the air stability of OLPL^18^. For example, Tang and co-workers utilized phosphonium salt to construct crystals, successfully realizing indigo blue OLPL lasting for more than 12 min in air (Chart S5)^19^. Lin and co-workers embedded carbon dots with cyanuric acid crystal to prevent the quenching from oxygen and water, thus achieving hour-level afterglow under ambient conditions^2^. Our group also developed a series of stable OLPL nanocrystals by a top-down method (Chart S5)^20^. While the crystallization aided in stabilizing excitons, it also came with several notable drawbacks, especially for the inferior processability. Besides crystallization, introducing dense polymer matrix could also restrict excitons quenching of oxygen to some extent^21–24^. Moreover, it contributed much to the film manufacturing and application processing. However, the structural uncertainty and inferior batch repeatability in synthesis would largely affect the resultant OLPL performance of polymer systems. Hence, developing a system that combines the advantages of both polymers and crystals is necessary for developing air-stable OLPL materials. It not only can stabilize excitons, but also facilitate processing and practical application.

**Fig. 1:**
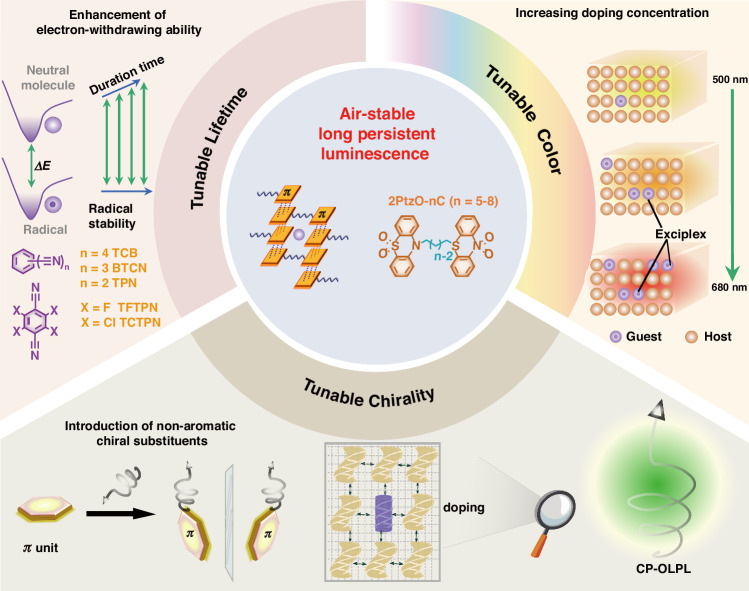
New platform for air-stable OLPL. Schematic representation of air-stable OLPL with tunable lifetime, color, and chirality

To simultaneously achieve exciton stabilization and material processability, a rigid-flexible cooperative molecular network was needed. π–π stacking, an efficient intermolecular interaction from crystalline systems, is known to enhance structural rigidity and suppress oxygen permeation, both of which are highly favorable for generating long-lived excitons and achieving efficient persistent emission^25^. Previous works about our groups and others have well demonstrated that the regulation of π–π stacking is an effective way to maintain the rigidity and prolong the emission lifetime of organic crystals^20,26,27^. In response to flexibility, the incorporation of a long alkyl chain between two rigid π-units should be a good choice. On one hand, the long alkyl chains could improve morphological adaptability, suppress excessive crystallization, and enhance film-forming ability. On the other hand, bilateral π-units offered more possibilities to form π–π stacking in amorphous or film state. Herein, phenothiazine 5,5-dioxide derivatives (2PtzO-nC, *n* = 5–8) with long alkyl chains bridging two π-units were selected as hosts to achieve OLPL, which have been demonstrated to show efficient room temperature phosphorescence (RTP) in the crystal state due to π-π stacking^28^. After melting, long alkyl chains served as a flexible skeleton, facilitating the film formation, while bilateral phenothiazine 5,5-dioxide groups interacted with adjacent molecules through dense π-π stacking to stabilize triplet excitons. With these, the advantages of both polymers and crystals could be integrated. After the TCB guest with the ability to form a stable radical anion being introduced^29^, the doping system where the phosphorescence host acted as an electron donor and guest as an electron acceptor, exhibited air-stable OLPL emission lasting for longer than 2 h under air (Fig. 1). Even after a month of being exposed to air, 1 h afterglow could still be maintained. Experimental results confirmed that the amorphous state with long-range disorder was dominant in this OLPL system, while dispersed microcrystals also existed. This hybrid structure simultaneously guaranteed the material processability and air stability of OLPL materials. Furthermore, the regulations of OLPL duration times (1.6–7154.3 s) and emission colors (500–680 nm) were realized by enhancing the electron-withdrawing ability and increasing the doping concentration of guest molecules. Additionally, when the non-aromatic chiral substituents were introduced into the phenothiazine 5,5-dioxide hosts, air-stable circularly polarized organic long persistent luminescence (CP-OLPL) was achieved for the first time.

## Results

### Phenomenon of air-stable OLPL

All compounds were purified and characterized prior to use. To rule out the potential influence of impurities on the OLPL behavior, high-performance liquid chromatography (HPLC) was performed to confirm the chemical purity of the compounds. All photophysical measurements were conducted only after verifying their high purity, ensuring that the observed OLPL characteristics originate solely from the intrinsic properties of the target molecules (Figs. S1–S3).

**Fig. 2:**
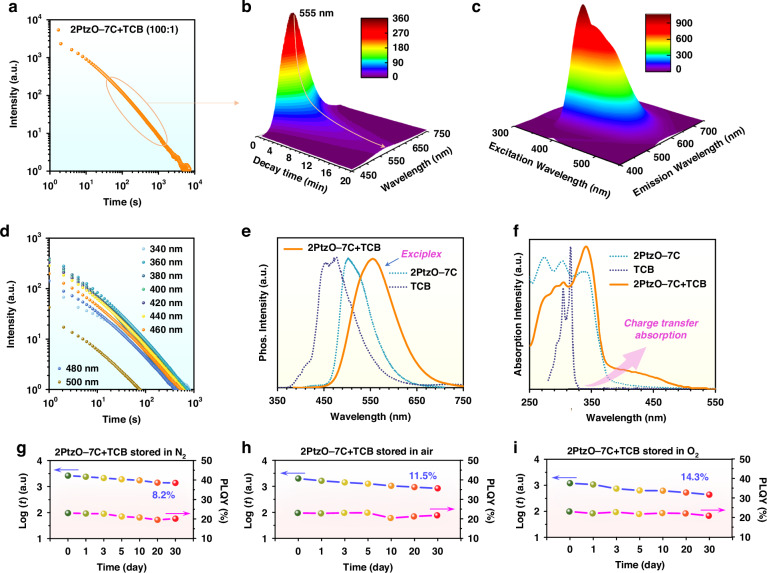
The OLPL behaviors for 2PtzO-7C + TCB system. **a** Time-resolved OLPL-decay curve of 2PtzO-7C + TCB (100:1) at atmosphere after 365 nm UV excitation. **b** Three-dimensional (3D) time-dependent OLPL spectra of 2PtzO-7C + TCB excited by a 365 nm UV lamp. **c** 3D excitation-emission mapping of 2PtzO-7C + TCB. **d** Time-resolved OLPL-decay curves of 2PtzO-7C + TCB excited by different wavelengths. **e** Afterglow emission spectra of 2PtzO-7C + TCB at room temperature, 2PtzO-7C in solid state and TCB in DCM solution at 77 K. **f** UV-Vis absorption spectra of 2PtzO-7C + TCB, 2PtzO-7C in solid state and TCB in DCM solution. **g** Variations of duration times and photoluminescence quantum yields (PLQYs) of 2PtzO-7C + TCB stored under nitrogen for different times. **h** Variations of duration times and photoluminescence quantum yields (PLQYs) of 2PtzO-7C + TCB stored under air for different times. **i** Variations of duration times and photoluminescence quantum yields (PLQYs) of 2PtzO-7C + TCB stored under oxygen for different times

Firstly, the photophysical properties of 2PtzO-nC (*n* = 5–8) host at different states were studied. Taking compound 2PtzO-7C with the best RTP performance as an example, after being melted and cooled to room temperature, it formed a smooth and rigid film with long-range disordered characteristics (Fig. S4a). Similar to its crystals, 2PtzO-7C melt exhibited a main peak at 510 nm (excimer) and a shoulder peak at 450 nm (monomer) in its RTP spectrum (Fig. S4b), indicating there were still abundant π-π stacking in long-range disordered skeleton^28^. Excitingly, a much prolonged RTP lifetime from 86.0 ms to 232.8 ms could be obtained for 2PtzO-7C after melting (Fig. S4c). This suggested the formation of dense and rigid film, benefiting from bilateral π-π stacking and long flexible alkyl chain, could efficiently restrict non-radiative transition and triplet quenching, just like polymers. Similar phenomena were found for other 2PtzO-nC (*n* = 5, 6, 8), further certifying the feasibility of forming polymer-like melts to stabilize triplet excitons (Figs. S5 and S6). According to previous reports, the formation of charge transfer (CT) state is primary for realizing OLPL^3–8^. In consideration of the weak electron-donating ability of phenothiazine 5,5-dioxide groups in 2PtzO-nC (*n* = 5–8), electron acceptor with matched energy level could be introduced as guest. After numerous material screenings, compound TCB was selected for two reasons. On one hand, it could interact with phenothiazine 5,5-dioxide group to form an exciplex for its electron-withdrawing ability. On the other hand, it could form a stable radical anion in some specific environment, which should be also important for the realization of OLPL. Then, 2PtzO-7C and TCB were mixed together with a mass ratio of 100:1 to form a doping molten system. As shown in Figs. 2a and S7, after the removal of 365 nm ultraviolet (UV) excitation, yellow-green emission of 2PtzO-7C + TCB could be detected under air for at least 2 h.

The HPLC diagram of 2PtzO-7C + TCB after UV excitation showed just two retention peaks corresponding to 2PtzO-7C and TCB, indicating the absence of photoreaction or photodegradation under the OLPL process (Fig. S8). To quantitatively evaluate this long emission property, the OLPL duration time (*t*) was defined as the time of emission intensity dropped to 1 detected by the Hitachi F-4600 fluorescence spectrophotometer. As shown in Fig. 2, 2PtzO-7C + TCB exhibited OLPL emission at 555 nm with a duration time up to 7154.3 s and photoluminescence quantum yield (PLQY) of 23.01%. Decay profile of 2PtzO-7C + TCB followed Debye–Edwards law (*t*^−m^, with m = 1.01), which further certified the OLPL character^30–35^. Compared to the individual afterglow emissions of the host at 510 nm in the molten state and the guest at 463 nm in the solution state at 77 K, exciplex should have formed between the electron donor and acceptor in 2PtzO-7C + TCB system. The newly formed absorption band around 450 nm, together with the significantly broadened excitation ranging from 340 to 500 nm, suggested the formation of a ground-state donor–acceptor static exciplex with charge-transfer character, rather than a simple superposition of host and guest absorptions (Fig. 2d, f)^36^. Then, Gaussian peak deconvolution was performed for the steady-state PL spectrum of the 2PtzO-7C + TCB system. As shown in Fig. S9, the spectrum could be well fitted by two bands centered at 543 nm and 590 nm, which were attributed to the residual host RTP and the exciplex emission, respectively. These two peaks showed similar OLPL decay curves; thus, the time-resolved OLPL spectra were consistent with the steady-state one (Figs. S9b and 2b). Because of the partial overlap between RTP emission of 2PtzO-7C host and the charge-transfer absorption of 2PtzO-7C + TCB, the Förster resonance energy transfer (FRET) between 2PtzO-7C host and the exciplex of the donor-acceptor pair could occur, which then affected the emission color and spectral shape of OLPL emission for the doping system (Fig. S9). Excitation power had a certain effect on this OLPL emission, in which stronger power led to longer OLPL duration time (Fig. S10). Besides, lowering the temperature could further enhance the emission intensity of OLPL, resulting from the restricted non-radiative transition (Fig. S11).

Delightfully, even being stored in air for 1 month, OLPL emission from 2PtzO-7C + TCB was still detectable for about 1 h after the UV excitation was removed, showing excellent air-stable property in comparison with traditional n-type amorphous systems (Fig. S12)^9^.

Furthermore, the OLPL performances of 2PtzO-7C + TCB after being stored in different environments for different times were studied in detail (Fig. S13 and Table S1). Duration time of 2PtzO-7C + TCB decreased by 8.2% in nitrogen after 1 month, 11.5% in air and only 14.3% even in pure oxygen (Fig. 2g–i). Unexpectedly, OLPL emission with a duration time of 186 s was also detected in 2PtzO-7C + TCB after being stored in air even for 1 year, which is a record in air-stability for OLPL systems (Fig. S13d). Besides, only slight floats appeared in PLQY of 2PtzO-7C + TCB in nitrogen, oxygen and air after 1 month. Even after being stored under water for 9 days, OLPL emission could be detected for 2PtzO-7C + TCB, exhibiting a good water-stability (Fig. S14).

### Mechanism of air-stable OLPL

To gain a deep understanding of the internal mechanisms behind OLPL emission, we conducted a study on the photophysical properties of TCB when doped in polymethyl methacrylate (PMMA) at 77 K. It was found to exhibit OLPL emission with a duration time up to 10^3 ^s (Fig. S15). Under UV excitation, an obvious electron spin resonance (ESR) signal at 328.7 mT with the *g* value of 2.0015 was detected for TCB doped in PMMA at 77 K (Fig. S16). Almost the same ESR signal was detected even after the removal of UV excitation for 20 min. Therefore, the stable TCB radical anions could be formed at 77 K after UV irradiation, even without a definite electron donor, which should be the main origin of its OLPL^17,29^. However, if PMMA was replaced by dichloromethane (DCM) solution, the emission lifetime would be much shortened at 77 K (Fig. S17). This indicated that the existence of the PMMA host has contributed much to the formation of TCB radical anions^37^. When TCB was doped into 2PtzO-7C through melting, the corresponding radical anions could be further stabilized (Fig. S15b). The resultant ESR signal after UV excitation presented some differences from that of TCB in PMMA at 77 K, showing the influence of host matrix on TCB radical anions. Furthermore, the decay tendency of ESR intensity met with power-law decay trend (Fig. S18), which well corresponded to the time-resolved OLPL-decay curve. This finding further confirmed the positive correlation between radical stability and OLPL duration time. Based on the analysis of the aforementioned photophysical properties and with the electron donor as host, a p-type charge diffusion mechanism was proposed (Fig. S15c). After UV irradiation, an electron can be excited from the HOMO to LUMO of donor (Ⅰ), then transfer to the LUMO of acceptor to form radical anion (Ⅱ). Secondly, holes will diffuse by the hopping of charges among the donor molecules, leading to the long-lived charge separated states (Ⅲ). When the diffused hole approaches a radical anion, the recombination between them can occur, resulting in the OLPL emission (Ⅳ). For the OLPL spectrum, both the RTP emission of the host (543 nm) and the exciplex emission (590 nm) of donor-acceptor pair were contained. Accordingly, their energy gap was calculated to be just 0.18 eV, which should be small enough to realize the exciton recycle between them, then contributing much to the prolonged OLPL emission^20^. Either the LUMO (−3.88 eV) of acceptor or the HOMO of (−5.62 eV) donor was lower than the reduction potential of oxygen (−3.50 eV) (Figs. S19–S21 and Table S2), which could effectively avoid the photoreaction to oxygen, thus promoting the air-stability of 2PtzO-7C + TCB in air^38,39^.

**Fig. 3:**
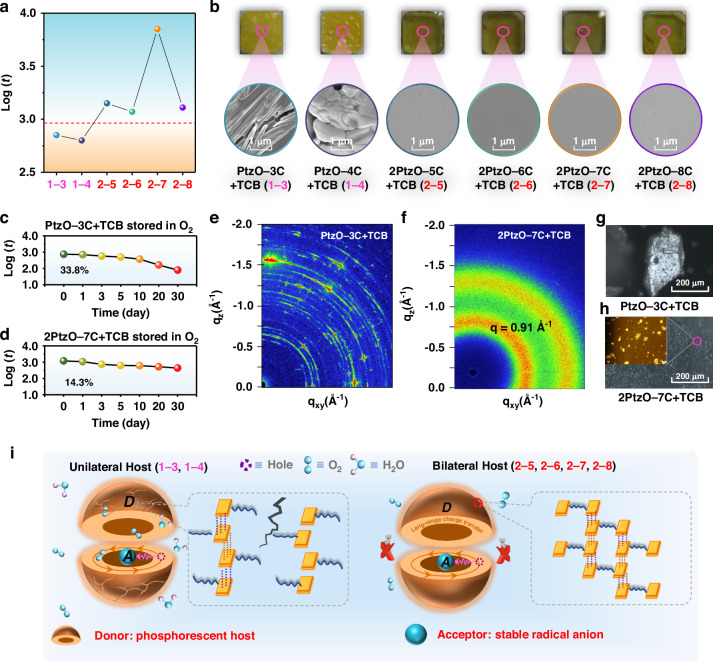
Mechanism of air-stable OLPL effect. **a** Duration times of PtzO-3C + TCB (1-3), PtzO-4C + TCB (1-4), 2PtzO-5C + TCB (2-5), 2PtzO-6C + TCB (2-6), 2PtzO-7C + TCB (2-7), 2PtzO-8C + TCB (2-8). **b** Photographs of 1-3, 1-4, 2-5, 2-6, 2-7, 2-8 taken at daylight (up) and corresponding scanning electron microscopy (SEM) diagrams (down). **c** Variation of OLPL duration times of PtzO-3C + TCB stored under oxygen for different times. **d** Variation of duration times of 2PtzO-7C + TCB stored under oxygen for different times. **e** Two-dimension (2D) small angle X-ray scatting spectra of PtzO-3C + TCB. **f** 2D small angle X-ray scatting spectra of 2PtzO-7C + TCB. **g** Polarized optical microscopic (POM) image of PtzO-3C + TCB. **h** POM image of 2PtzO-7C + TCB. Inset: partial enlarged drawing. **i** Schematic illustration of unilateral and bilateral host packing in the molten state

Then, the OLPL performance of mechanically ground 2PtzO-7C + TCB powder was studied to verify the process of long-range charge diffusion. As shown in Fig. S15e, after grinding, the duration time of 2PtzO-7C + TCB decreased from 10^3 ^s to 10^2 ^s while PLQY increased from 23.01% to 30.75%. This indicated that the reduced size could shorten the charge diffusion distance, thus increasing charge recombination probability. In comparison with photoreaction with oxygen, the suppression of energy transfer from triplet excitons to oxygen should be more important to realize air-stable OLPL. Therefore, we conducted a comprehensive study on how various hosts influence the air stability of OLPL emission (Fig. 3). As shown in Fig. 3a, the duration times of molten mixtures with bilateral phenothiazine 5,5-dioxide derivatives as host (2PtzO-5C + TCB (2-5) (1412.5 s), 2PtzO-6C + TCB (2-6) (1174.9 s), 2PtzO-7C + TCB (2-7) (7154.3 s), 2PtzO-8C + TCB (2-7) (1288.5 s)) were all longer than unilateral host (PtzO-3C + TCB (1-3) (707.9 s), PtzO-4C + TCB (1-4) (630.1 s)) (Fig. S22 and Table S3).

Based on scanning electron microscope (SEM) images, it was easy to find that 1-3 and 1-4 had numerous cracks, while 2-5, 2-6, 2-7 and 2-8 presented dense and uniform surfaces (Figs. 3b and S23–S28). According to the long-range charge diffusion mechanism, the presence of cracks in 1-3 and 1-4 would obstruct the path of charge diffusion and increase the quenching probability of oxygen through energy transfer, thus consequently shortening the OLPL duration and lowering the air-stability. Taking 1-3 as an example, its OLPL duration time decreased by 33.8% after being exposed to oxygen for a month, 35.1% in air, and 14.9% in nitrogen, indeed indicating poorer air stability of OLPL compared to the molten mixture of 2-7 (Figs. 3c, d and S29). Nevertheless, their PLQY showed negligible change when stored under different conditions for different days, since the quenching effect of oxygen on short-lived excitons was much weaker (Table S4).

Simultaneously, the water stability of 1-3 was also found to be inferior compared to 2-7 (Fig. S30). Powder X-ray diffraction (PXRD) patterns showed that the incorporation of TCB has a minimal impact on the molecular packing of PtzO-3C host (Fig. S31). And their molten mixtures were still dominated by crystalline phase, which would induce grain boundaries and cracks easily (Fig. S32). On the contrary, molten mixtures based on a bilateral host were amorphous (Fig. S33). Using 2PtzO-7C + TCB as an example, neither a sharp peak in the PXRD pattern nor a pronounced scattering point in small-angle X-ray scattering (SAXS) was found (Fig. 3e, f), suggesting the presence of a long-range disordered structure. Interestingly, short-range ordered grain crystals with size of about 1–40 μm (luminous yellow) could be observed through polarized optical microscopic (POM) (Figs. 3g, h and S34, S35). The formation of grains in 2PtzO-7C + TCB might be attributed to the intense interaction between donor and acceptor, which further increased the rigidity of the OLPL system, thus enhanced the air-stability. View from the single crystal structure of 2PtzO-7C in Fig. S36, there were bilateral π-π stacking within adjacent molecules, which could facilitate the formation of dense and rigid quasi-polymer structure without grain boundary. This should be the main origin for the much better OLPL performance in the molten mixture with a bilateral host. Besides, glass-transition and cold crystallization peaks were detected in all of the mixtures with bilateral host, further certifying their polymer-like characteristics (Figs. S37 and S38). Notably, the melting points (235–246 °C) of the doping systems based on bilateral phenothiazine 5,5-dioxide derivatives were obviously higher than those (149–197 °C) based on unilateral host, indicating their better rigidity. With it, the non-radiative transition would be largely restricted, thereby promoting the OLPL performance. In addition, the absorption and excitation spectra for the single-component and doping systems were carefully compared (Figs. S39–S41). The doping systems of 2PtzO-nC+TCB (*n* = 5–8) were found to show more obvious CT absorption than PtzO-nC (*n* = 3–4) in UV-Vis absorption spectra, while the corresponding excitation spectra were similarly red-shifted. This indicated more efficient donor-acceptor interactions with bilateral π-units for host molecules at the ground state.

### Tunable OLPL lifetime by enhancing the electron-withdrawing ability of guest molecules

To study the influence of guests on OLPL performance, photophysical properties of other acceptor molecules like BTCN, TPN, TFTPN, TCTPN were systematically studied (Figs. S42–S46 and Table S5). Energy level measurements revealed that the lowest unoccupied molecular orbital (LUMO) levels of the other four acceptor molecules (–3.76 to –3.86 eV) were very close to that of TCB (–3.88 eV), as shown in Fig. S20 and Table S2. When they were doped into 2PtzO-7C through melting, red-shifted emission could be observed for all of them, indicating the formation of an exciplex (Fig. S44). However, these five molten mixtures exhibited different OLPL duration times from 1.6 to 7154.3 s under an atmosphere (Figs. S45 and S46 and Table S5). A similar changing trend could also be found for the corresponding acceptors doped in PMMA at 77 K (Figs. S47 and S48). Thus, regardless of the similar molecular structure and energy level, the change of acceptor could still affect the resultant OLPL performance in doping systems.

**Fig. 4:**
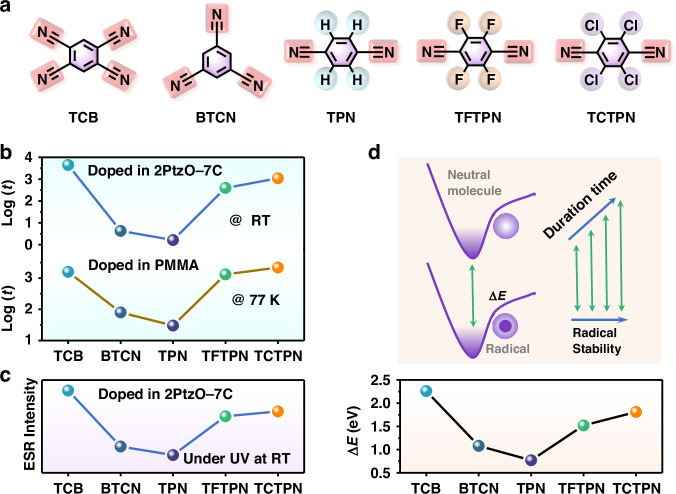
Tunable OLPL lifetimes with the regulation of guests. **a** Chemical structures of five acceptor molecules. **b** OLPL duration times of five acceptors melted in 2PtzO-7C at room temperature (up) and doped in PMMA at 77 K (down). **c** ESR intensity of five molten mixtures under UV light at room temperature. **d** Energy gaps between neutral molecules and corresponding radicals

To make clear the internal mechanism, their ESR spectra were measured. Not surprisingly, variations in ESR intensity were found for these five different molten mixtures after UV irradiation, which corresponded well with the variation of OLPL duration time for the relevant acceptors doped in PMMA at 77 K, as well as melted in 2PtzO-7C (Figs. 4a–c and S49, S50). These results further proved there was a positive correlation between the radical stability of guest molecules and the OLPL duration of molten mixtures. Compared with the ESR spectra of guests doped in PMMA at 77 K, the signals of molten mixtures showed some differences in peak shape, indicating the significant influence of host on the corresponding radical anions of acceptor (Figs. S49 and S50). Besides, tetrabenzocarboxylic acid (PA), with an electronic structure similar to TCB, could also interact with the host to form an exciplex. However, due to its inability to form stable radicals, no OLPL was detected when it was co-melted with 2PtzO-7C (Fig. S51). On the other hand, more obvious CT absorption could be found in the UV-Vis absorption spectra of 2PtzO-7C + TCB/TFTPN/TCTPN than those of 2PtzO-7C + BTCN/TPN (Figs. 4d and S52), suggesting their stronger donor-acceptor interactions. This could also contribute much to the OLPL effect.

Also, the energy gap (Δ*E*) between neutral molecule and radical of acceptor was calculated to further explain the stability of radical anion (Fig. 4d). It was found that larger energy gap (Δ*E*) could lead to better radical stability. This, in turn, enhanced the ESR signal and extended the OLPL duration of molten mixtures. A stable radical anion is not merely a fundamental requirement for OLPL duration but also a critical prerequisite for the realization of air-stable OLPL.

### Tunable OLPL color with increasing the doping concentrations of guest molecule

**Fig. 5:**
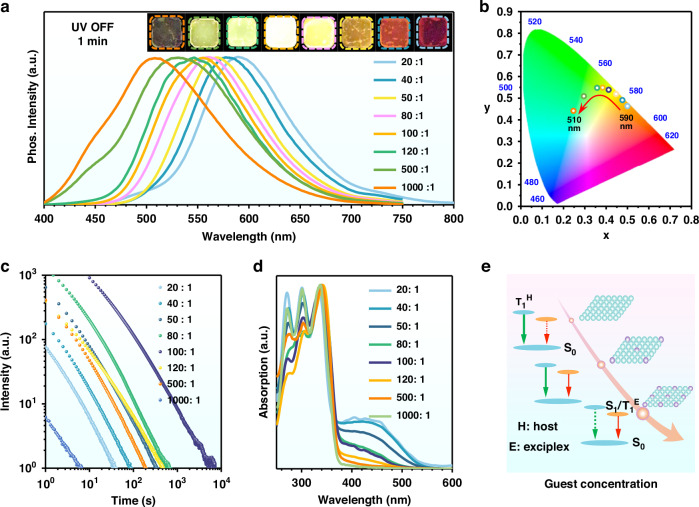
Tunable OLPL colors with doping concentrations of guest molecule. **a** Afterglow emission spectra of 2PtzO-7C + TCB with different ratios. Inset: photographs of 2PtzO-7C + TCB with different ratios after removing UV excitation. **b** Commission Internationale de L’Eclairage (CIE) coordinates over different concentrations. **c** Time-resolved OLPL-decay curves of 2PtzO-7C + TCB with different ratios. **d** UV-Vis absorption spectra of 2PtzO-7C + TCB with different ratios. **e** Schematic illustration of tunable OLPL colors for 2PtzO-7C + TCB with different guest ratios

Further on, color-tunable OLPL emissions were achieved through introducing different concentrations of guest molecules into doping system. Upon increasing the host-guest ratio from 1000:1 to 20:1, there was a notable red shift in OLPL emission, extending the wavelength from 500 nm to 680 nm (Fig. 5a). This change in OLPL emission color could be clearly evidenced by Commission Internationale de l’Eclairage (CIE) coordinates, as shown in Fig. 5b. Color-tunable OLPL emission could not only be excited by UV light but also by the flashlight of iPhone 13 (Figs. S53 and S54). As the doping concentration of the guest was elevated, the duration of OLPL was first extended and then gradually shortened (Fig. 5c). This phenomenon can be attributed to the variance in charge diffusion distances between the donor and acceptor. Mixtures that possessed a higher concentration of TCB exhibited shortened charge diffusion distances, facilitating more rapid charge recombination, and consequently, a shorter duration of OLPL.

**Fig. 6:**
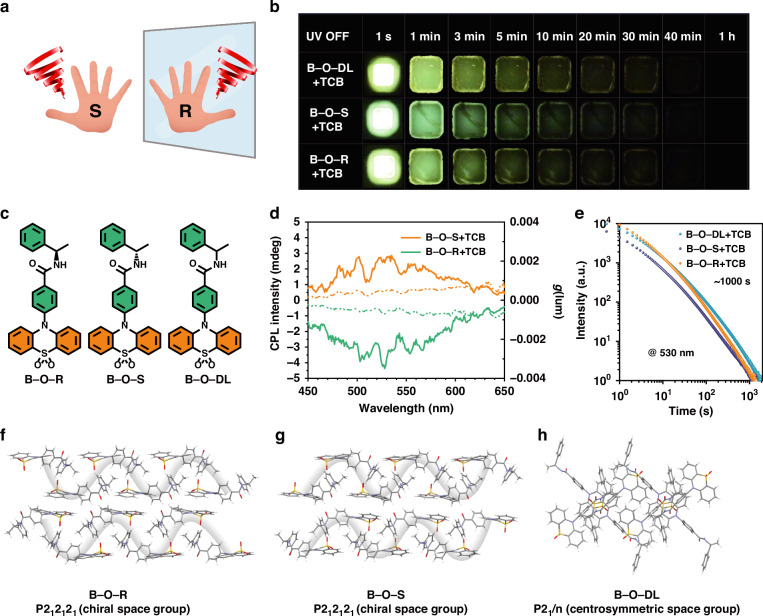
Air-stable circularly polarized OLPL. **a** Concept diagram of chiral molecules. **b** OLPL images of B-O-S, B-O-R, and B-O-DL with TCB complexes at different time intervals. **c** Chemical structures of B-O-R, B-O-S, and B-O-DL molecules. **d** CPL spectra of B-O-S + TCB and B-O-R + TCB. **e** Time-resolved luminescence decay curves of B-O-DL + TCB, B-O-S + TCB, and B-O-R + TCB at 530 nm. **f** Crystal structure of B-O-R. **g** Crystal structure of B-O-S. **h** Crystal structure of B-O-DL

As for the mixtures with a lower concentration of guest, too longer charge diffusion distance and fewer charge recombination sites were presented, which would also shorten duration time. Therefore, the appropriate concentration was also important for achieving OLPL. Besides, the influence of guest concentration on OLPL color was studied. As shown in Fig. 6d, enhanced absorption at 450 nm could be observed as the TCB doping concentration increased, which suggested a greater degree of charge transfer. And, OLPL excitation wavelength of 2PtzO-7C + TCB widened with increasing TCB doping concentration (Figs. S55–S58). Because of the different CT effects, 2PtzO-7C + TCB samples with different ratios of TCB were found to exhibit different ESR signals (Figs. S59 and S60). Further on, the emission ratios between host (~510 nm) and exciplex (~590 nm) were calculated for the doping systems with different guest concentrations. As shown in Fig. S61, with the increase of doping concentration for guest molecules, the ratio decreased from 2.72 (1000:1), to 1.58% (100:1), then to nearly 0 (20:1). Therefore, the changed emission ratio between host and exciplex should be mainly responsible to the tunable OLPL emission color. Such phenomena have seldom been reported in previous literature, which might open up a new way to regulate the OLPL emission color.

### Tunable OLPL chirality with the introduction of non-aromatic substituents to host molecules

Moreover, by introducing a non-aromatic chiral substituent into the phenothiazine 5,5’-dioxide core, we were able to induce chirality effectively, further enriching the emission properties of OLPL systems. As shown in Fig. 6, through altering the configurations and positions of the chiral center, another six phenothiazine 5,5’-dioxide hosts were designed and synthesized, namely A-O-S/R/DL and B-O-S/R/DL (Figs. S62–S65). When they were mixed with TCB guest to form doping systems, air stable circularly polarized organic long persistent luminescence (CP-OLPL) was successfully achieved for the first time (Figs. S66–S74 and Tables S6, S7). Interestingly, amorphous state could also be obtained for these doping films after thermal melting (Fig. S66), which should mainly originate from the asymmetric chiral configuration to inhibit crystallization and the stabilizing effect to amorphous aggregate state from intermolecular hydrogen bonding between amide groups.

Taking the systems of B-O-S/R/DL + TCB as examples, after excitation, hour-level OLPL emissions could be captured under atmospheric conditions (Fig. 6a–e). Correspondingly, the OLPL duration times were measured to be approximately 1200 s, while PLQYs were about 19%. After the introduction of chiral substituents with different configurations into host molecules, B-O-S/R + TCB were found to exhibit opposite circular dichroism (CD) signals (Fig. S73). In addition, their CPL spectra, in good accordance to the OLPL emission bands, showed mirror-image CPL signals with |*g*_lum_| values of about 0.58 × 10^−3^ at 540 nm, which was rarely reported in OLPL systems (Fig. S74). Similar CPL behaviors were observed in the systems of A-D-S/R + TCB, while their OLPL duration times could be further prolonged to about 2000 s under atmospheric conditions (Fig. S68). These results validated the strategy of introducing chiral groups into host molecules to achieve CPL emission in the OLPL doping systems. It should be noted that the |*g*_lum_| values for OLPL systems are not very high, no matter for this work or others without air stability^40^. Two main reasons should account for it. On the one hand, the asymmetric configurations of host molecules were induced by the chiral carbon atom, rather than the chiral axis. On the other hand, the CPL emissions were from the assemblies of host and guest molecules, while the amorphous state restricted the formation of ordered aggregates. Therefore, to realize CP-OLPL with high |*g*_lum_| values, the introduction of host and/or guest molecules with a chiral axis characteristic might be an efficient way.

To make a deep insight into the mechanism, the single crystal structures of host molecules were analyzed (Figs. 6f–h and S75, S76 and Table S8). Due to the introduction of non-aromatic chiral substituents, B-O-S/R crystals both presented a chiral space group of P2_1_2_1_2_1_, while the racemic B-O-DL crystal gave a centrosymmetric space group of P2_1_/n. These indicated that the introduction of non-aromatic chiral substituents into phenothiazine 5,5’-dioxide could effectively induce the chiral arrangement of host molecules, thus leading to the CPL emissions in OLPL systems.

Besides, obvious intermolecular π-π interactions within adjacent phenothiazine 5,5’-dioxide groups were observed in the host crystals, in which the π-π distance (*d*) and corresponding dihedral angle (*θ*) were both 3.87 Å and 11.3° for B-O-S/R crystals. Similarly, chiral arrangement and intermolecular π-π stacking were presented in A-O-S/R crystals, regardless of the changed positions for the chiral center. Specifically, bilateral π-π stackings based on adjacent phenothiazine 5,5’-dioxide and benzamide groups were found for them, whose π-π distances were measured to be as short as 3.07 Å. This should be the main reason for the longer OLPL duration times of A-O-S/R than those of B-O-S/R.

### Applications

To showcase the practical potential of these OLPL materials, we fabricated a wearable ring by embedding the 2PtzO-7C + TCB system into stone-plastic clay, a common hand-moldable, air-dried material. As illustrated in Fig. S77, the ring can be effectively excited not only by laboratory UV sources but also by a standard smartphone flashlight. This enables convenient and portable activation of long afterglow emission without specialized equipment. The bright and persistent luminescence in ambient conditions allows this material to serve as a visible wearable ornament during nighttime outdoor activities. Such proof-of-concept wearable devices underscore the feasibility of deploying OLPL materials in decorative, safety, or emergency indication applications, thus highlighting their functional advantages over conventional short-lived luminescent materials^41–46^.

## Discussion

In this work, we developed an air-stable OLPL system through combining the advantages of both polymers and crystals. Specifically, the OLPL emission of 2PtzO-7C + TCB could last for at least 2 h in air and even 1 h after being exposed to air for 1 month. Careful studies indicated three factors were mainly responsible for the excellent air-stability of OLPL: (1) Flexible phosphorescent host could form dense and rigid film to cut off contact with oxygen. (2) Donor host and acceptor guest went through p-type charge transfer process where the charge diffused on the HOMO of host. At this time, a deeper HOMO of host than the reduction potential of oxygen (−3.5 eV) would prevent the photoreaction with oxygen. (3) Acceptor guest showed the ability to form an air-stable radical anion, which needed to recombine with the obtained radical cations after long-range charge diffusion. Based on this newly developed air-stable OLPL platform, the regulations of OLPL duration times and emission colors were realized with enhancing the electron-withdrawing ability and increasing the doping concentration of guest molecules, respectively, which further enriched the air-stable OLPL systems. At last, by incorporating non-aromatic chiral substituents into the phenothiazine 5,5’-dioxide core, the chiral arrangements of host molecules were effectively induced, which then led to the air-stable CP-OLPL for the first time.

## Materials and methods

### Characterization

High-performance liquid chromatogram (HPLC) spectra were recorded on an Agilent 1100 HPLC. Differential scanning calorimeter (DSC) curves were recorded on the Differential Scanning Calorimeter DSC 214 polyma. UV-Vis spectra were measured on a Shimadzu UV-2600. Photoluminescence (PL) spectra and time-resolved OLPL decay curves were performed on a Hitachi F-4600 fluorescence spectrophotometer (Excitation/emission slit widths: For phosphorescence spectra and lifetimes: 20 nm/20 nm; For PL measurements: 5 nm/5 nm). Photoluminescence quantum yields and temperature-dependent PL spectra were determined with the FLS1000 spectrometer. Powder X-ray diffraction (PXRD) patterns were recorded by Rigaku Smartlab9KW. Ultraviolet Photoelectron Spectroscopy (UPS) was detected by Thermo Fischer ESCALAB 250Xi from America. Electron-spin resonance (ESR) spectra were recorded on a JEOL JES-FA200 spectrometer. Cyclic voltammetry (CV) curves were determined using a CHI660E electrochemical workstation. Scanning electron microscope (SEM) pictures were recorded on Thermo Fisher Scientific Apreo S LoVac. Small-angle X-ray scatting (SAXS) spectra were detected by Anton Paar SASess MC2. Polarized optical microscopic (POM) images were recorded by an Olympus GX71 polarizing microscope. Circular dichroism (CD) spectra were measured using a JASCO J-1700, MOS-200M, and SFM-4000 setup. Circularly polarized luminescence (CPL) measurements were performed at steady-state mode using a JASCO CPL-300 spectrometer. OLPL photographs were acquired with a Canon EOS 80D digital camera. The camera was set to ISO 25600 and aperture f/5.6. The exposure time was adjusted according to the duration after UV irradiation: For the first image: 5 s/1–10 min: 10 s/20 min to 1 h: 30 s/>1 h: 40 s. Sample were excited by 365 nm UV lamp (power density: 8 mW cm^-2^) for about 30 s.

### Materials

TCB, BTCN, TPN, TFTPN, and TCTPN were purchased from Bide Pharmatech Ltd. and purified by column chromatography. 2PtzO-nC (*n* = 5, 6, 7, 8) and PtzO-nC (*n* = 3, 4) were synthesized according to literature^28,47^.

### Film fabrication

2PtzO-7C + TCB was a molten mixture with a mass ratio of 100:1 (2PtzO-7C: TCB), fabricated by melt-casting method, where 2PtzO-7C and TCB powder mixtures were heated up to 300 °C (over the melting point) under air and then cooled to room temperature naturally. Other molten mixtures were obtained by the same preparation method as 2PtzO-7C + TCB. The sample films were prepared with dimensions of 0.5 × 0.5 cm and a thickness of approximately 1.3 mm.

### Theoretical calculations

The energies of neutral guest molecules and corresponding radicals were calculated with the Gaussian 09 program at the level of M062X/6-31 g*.

## Supplementary information

Supplemental figures and tables

Cif file of A-O-DL crystal

Cif file of A-O-R crystal

Cif file of A-O-S crystal

Cif file of B-O-DL crystal

Cif file of B-O-R crystal

Cif file of B-O-S crystal

Checkcif file of A-O-DL crystal

Checkcif file of A-O-R crystal

Checkcif file of A-O-S crystal

Checkcif file of B-O-DL crystal

Checkcif file of B-O-R crystal

Checkcif file of B-O-S crystal

## Data Availability

The authors declare that the data supporting the findings of this study are provided in the Information file. All data are available from the authors upon request.
