# Supplementary material for: A new platform for achieving long-term air-stable long persistent luminescence in amorphous organic system
Source: Light Sci Appl. 2026 Aug 18;15:344. doi: 10.1038/s41377-026-02367-6 (PMC13487193; doi:10.1038/s41377-026-02367-6)

checkCIF/PLATON report

Structure factors have been supplied for datablock(s) B-O-DL

THIS REPORT IS FOR GUIDANCE ONLY. IF USED AS PART OF A REVIEW PROCEDURE FOR PUBLICATION, IT SHOULD NOT REPLACE THE EXPERTISE OF AN EXPERIENCED CRYSTALLOGRAPHIC REFEREE.

No syntax errors found. CIF dictionary Interpreting this report

Datablock: B-O-DL

```
Bond precision:   C-C = 0.0055 A            Wavelength=0.71073

Cell:             a=12.5614(8)     b=9.5106(5)        c=18.4350(12)
                  alpha=90         beta=96.976(2)     gamma=90
Temperature:      296 K

                 Calculated                   Reported
Volume           2186.1(2)                    2186.1(2)
Space group      P 21/n                       P 1 21/n 1
Hall group       -P 2yn                       -P 2yn
Moiety formula   C27 H22 N2 O3 S              C27 H22 N2 O3 S
Sum formula      C27 H22 N2 O3 S              C27 H22 N2 O3 S
Mr               454.53                       454.52
Dx,g cm-3        1.381                        1.381
Z                4                            4
Mu (mm-1)        0.182                        0.182
F000             952.0                        952.0
F000'            952.89
h,k,lmax         15,11,22                     15,11,22
Nref             4175                         4169
Tmin,Tmax                                     0.614,0.745
Tmin'

Correction method= # Reported T Limits: Tmin=0.614 Tmax=0.745
AbsCorr = MULTI-SCAN

Data completeness= 0.999           Theta(max)= 25.705

R(reflections)= 0.0730( 3427)                          wR2(reflections)=
                                                       0.1885( 4169)
S = 1.065                    Npar= 299
```

The following ALERTS were generated. Each ALERT has the format
test-name_ALERT_alert-type_alert-level.
Click on the hyperlinks for more details of the test.

Alert level C

```
PLAT053_ALERT_1_C Minimum Crystal Dimension Missing (or Error) ...     Please Check
PLAT054_ALERT_1_C Medium  Crystal Dimension Missing (or Error) ...     Please Check
PLAT055_ALERT_1_C Maximum Crystal Dimension Missing (or Error) ...     Please Check
PLAT230_ALERT_2_C Hirshfeld Test Diff for    S01       --O004     .       5.2 s.u.
PLAT230_ALERT_2_C Hirshfeld Test Diff for    C00V      --C00W     .       5.2 s.u.
PLAT242_ALERT_2_C Low    'MainMol' Ueq as Compared to Neighbors of       C00K Check
PLAT340_ALERT_3_C Low Bond Precision on  C-C Bonds ...............    0.00552 Ang.
PLAT906_ALERT_3_C Large K Value in the Analysis of Variance ......      6.896 Check
PLAT976_ALERT_2_C Check Calcd Resid. Dens.  0.67Ang From O004    .      -0.41 eA-3
```

Alert level G

```
PLAT003_ALERT_2_G Number of Uiso or U(i,j)  Restrained non-H-Atoms         33 Report
PLAT007_ALERT_5_G Number of Unrefined Donor-H Atoms ..............          1 Report
              H006
PLAT177_ALERT_4_G The CIF-Embedded .res File Contains DELU Records          1 Report
PLAT720_ALERT_4_G Number of Unusual/Non-Standard Labels ..........         55 Note
              S01     O002    N003    O004    O005    N006    H006    C007
              C008    H008    C009    C00A    C00B    H00B    C00C    H00C
              C00D    H00D    C00E    H00E    C00F    C00G    C00H    C00I
              C00J    H00J    C00K    C00L    H00L    C00M    H00M    C00N
              H00N    C00O    H00O    C00P    H00P    C00Q    H00Q    C00R
              H00R    C00S    H00S    C00T    H00T    C00U    H00A    H00F
              H00G    C00V    H00V    C00W    H00W    C00X    H00X
PLAT793_ALERT_4_G Model has Chirality at C00S         (Centro SpGr)          R Verify
PLAT860_ALERT_3_G Number of Least-Squares Restraints .............         91 Note
PLAT910_ALERT_3_G Missing # of FCF Reflection(s) Below Theta(Min).          1 Note
               -1   0   1,
PLAT912_ALERT_4_G Missing # of FCF Reflections Above STh/L=  0.600          5 Note
PLAT969_ALERT_5_G The 'Henn et al.' R-Factor-gap value ...........      4.197 Note
              Predicted wR2: Based on SigI**2  4.49 or SHELX Weight 17.70
PLAT978_ALERT_2_G Number C-C Bonds with Positive Residual Density.          5 Info
```

```
   0 ALERT level A = Most likely a serious problem - resolve or explain
   0 ALERT level B = A potentially serious problem, consider carefully
   9 ALERT level C = Check. Ensure it is not caused by an omission or oversight
  10 ALERT level G = General information/check it is not something unexpected

   3 ALERT type 1 CIF construction/syntax error, inconsistent or missing data
   6 ALERT type 2 Indicator that the structure model may be wrong or deficient
   4 ALERT type 3 Indicator that the structure quality may be low
   4 ALERT type 4 Improvement, methodology, query or suggestion
   2 ALERT type 5 Informative message, check
```

It is advisable to attempt to resolve as many as possible of the alerts in all categories. Often the minor alerts point to easily fixed oversights, errors and omissions in your CIF or refinement strategy, so attention to these fine details can be worthwhile. In order to resolve some of the more serious problems it may be necessary to carry out additional measurements or structure refinements. However, the purpose of your study may justify the reported deviations and the more serious of these should normally be commented upon in the discussion or experimental section of a paper or in the "special_details" fields of the CIF. checkCIF was carefully designed to identify outliers and unusual parameters, but every test has its limitations and alerts that are not important in a particular case may appear. Conversely, the absence of alerts does not guarantee there are no aspects of the results needing attention. It is up to the individual to critically assess their own results and, if necessary, seek expert advice.

Publication of your CIF in IUCr journals

A basic structural check has been run on your CIF. These basic checks will be run on all CIFs submitted for publication in IUCr journals (*Acta Crystallographica*, *Journal of Applied Crystallography*, *Journal of Synchrotron Radiation*); however, if you intend to submit to *Acta Crystallographica Section C* or *E* or *IUCrData*, you should make sure that full publication checks are run on the final version of your CIF prior to submission.

Publication of your CIF in other journals

Please refer to the *Notes for Authors* of the relevant journal for any special instructions relating to CIF submission.

PLATON version of 19/12/2024; check.def file version of 19/12/2024

Datablock B-O-DL - ellipsoid plot

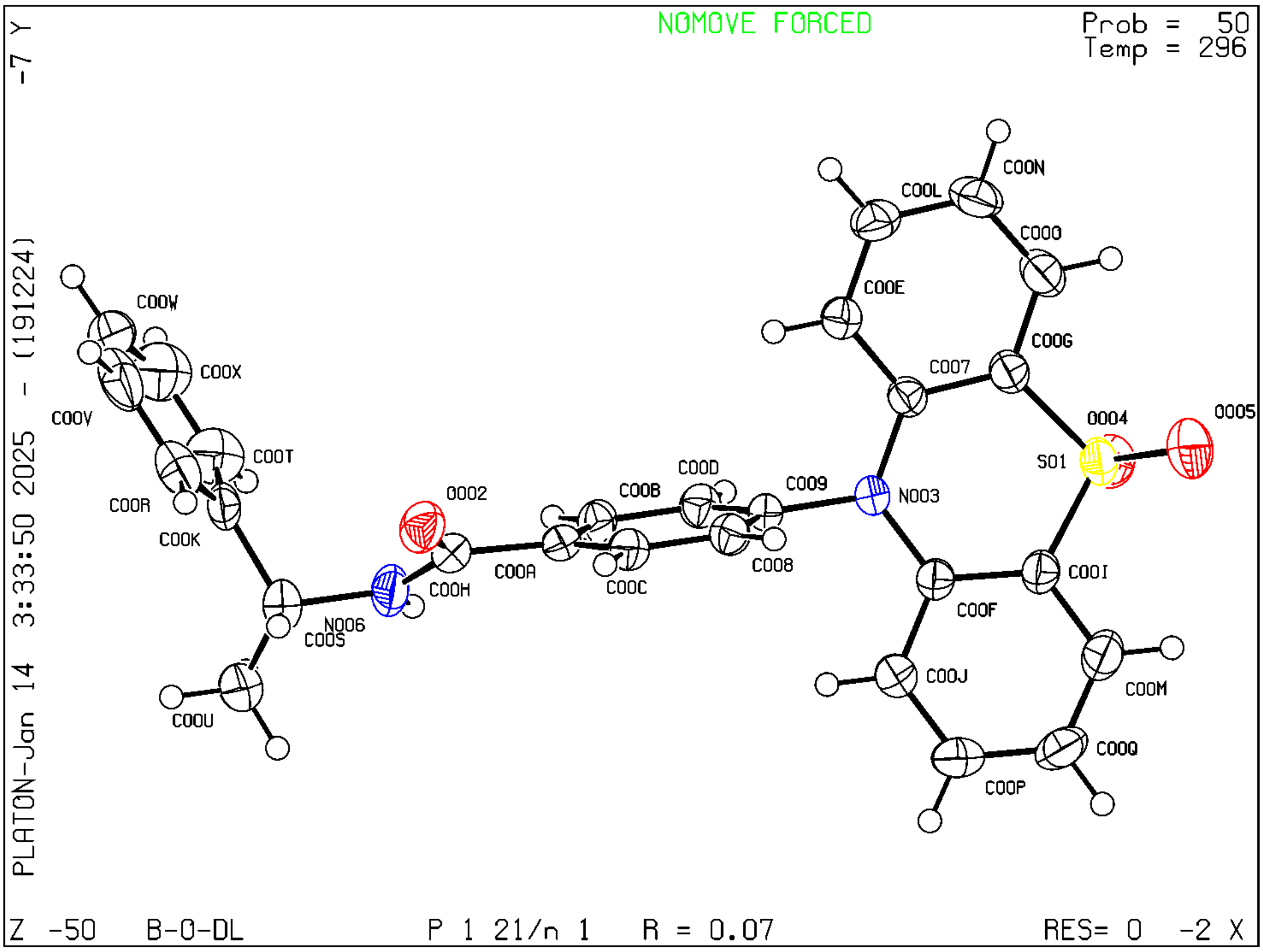

Supplement: Supplementary file 2 [file 41377_2026_2367_MOESM11_ESM.pdf]
