# Supplementary material for: A new platform for achieving long-term air-stable long persistent luminescence in amorphous organic system
Source: Light Sci Appl. 2026 Aug 18;15:344. doi: 10.1038/s41377-026-02367-6 (PMC13487193; doi:10.1038/s41377-026-02367-6)

checkCIF/PLATON report

Structure factors have been supplied for datablock(s) B-O-S

THIS REPORT IS FOR GUIDANCE ONLY. IF USED AS PART OF A REVIEW PROCEDURE FOR PUBLICATION, IT SHOULD NOT REPLACE THE EXPERTISE OF AN EXPERIENCED CRYSTALLOGRAPHIC REFEREE.

No syntax errors found.   CIF dictionary   Interpreting this report

Datablock: B-O-S

Bond precision:	C-C = 0.0054 A		Wavelength=0.71073
Cell:	a=9.0981(10)	b=12.1380(16)	c=20.349(3)
	alpha=90	beta=90	gamma=90
Temperature:	296 K		

	Calculated	Reported
Volume	2247.2(5)	2247.2(5)
Space group	P 21 21 21	P 21 21 21
Hall group	P 2ac 2ab	P 2ac 2ab
Moiety formula	C27 H22 N2 O3 S	C27 H22 N2 O3 S
Sum formula	C27 H22 N2 O3 S	C27 H22 N2 O3 S
Mr	454.53	454.52
Dx,g cm-3	1.344	1.343
Z	4	4
Mu (mm-1)	0.177	0.177
F000	952.0	952.0
F000’	952.89	
h,k,lmax	11,15,25	11,15,25
Nref	4660[2653]	4609
Tmin,Tmax		0.607,0.745
Tmin’		

Correction method= # Reported T Limits: Tmin=0.607 Tmax=0.745
AbsCorr = MULTI-SCAN

Data completeness= 1.74/0.99   Theta(max)= 26.479

R(reflections)= 0.0512(3886)   wR2(reflections)= 0.1158(4609)

S = 1.069   Npar= 299

The following ALERTS were generated. Each ALERT has the format
test-name_ALERT_alert-type_alert-level.
Click on the hyperlinks for more details of the test.

Alert level C

```
PLAT053_ALERT_1_C Minimum Crystal Dimension Missing (or Error) ...     Please Check
PLAT054_ALERT_1_C Medium  Crystal Dimension Missing (or Error) ...     Please Check
PLAT055_ALERT_1_C Maximum Crystal Dimension Missing (or Error) ...     Please Check
PLAT340_ALERT_3_C Low Bond Precision on  C-C Bonds ...............    0.00544 Ang.
PLAT911_ALERT_3_C Missing FCF Refl Between Thmin & STh/L=    0.600         15 Report
                   2  0  0,   2  1  0,   0  2  0,   1  2  0,   2  2  0,   1  3  0,
                   1  0  2,   1  1  2,   0  3  2,   1  0  3,   1  1  3,   0  0  4,
                   0  1  4,   0  1  5,   0  0  6,
PLAT913_ALERT_3_C Missing # of Very Strong Reflections in FCF ....          8 Note
                   0  2  0,   2  2  0,   0  3  2,   1  1  3,   0  0  4,   0  1  4,
                   0  1  5,   0  0  6,
```

Alert level G

```
PLAT007_ALERT_5_G Number of Unrefined Donor-H Atoms .............           1 Report
              H006
PLAT720_ALERT_4_G Number of Unusual/Non-Standard Labels ..........         55 Note
              S001    O002    O003    N004    O005    N006    H006    C007
              H007    C008    H008    C009    H009    C00A    H00A    C00B
              C00C    C00D    C00E    C00F    C00G    C00H    H00H    C00I
              H00I    C00J    H00J    C00K    H00K    C00L    H00L    C00M
              H00M    C00N    C00O    H00O    C00P    H00P    C00Q    C00R
              H00R    C00S    H00S    C00T    H00T    C00U    H00U    C00V
              H00V    C00W    H00B    H00C    H00D    C00X    H00X
PLAT791_ALERT_4_G Model has Chirality at C00P       (Sohncke SpGr)          S Verify
PLAT910_ALERT_3_G Missing # of FCF Reflection(s) Below Theta(Min).          2 Note
                   0  1  1,   0  0  2,
PLAT912_ALERT_4_G Missing # of FCF Reflections Above STh/L=  0.600          9 Note
PLAT969_ALERT_5_G The 'Henn et al.' R-Factor-gap value ..........       2.074 Note
              Predicted wR2: Based on SigI**2  5.58 or SHELX Weight 10.83
PLAT978_ALERT_2_G Number C-C Bonds with Positive Residual Density.          3 Info
```

---

0 **ALERT level A** = Most likely a serious problem - resolve or explain
0 **ALERT level B** = A potentially serious problem, consider carefully
6 **ALERT level C** = Check. Ensure it is not caused by an omission or oversight
7 **ALERT level G** = General information/check it is not something unexpected

3 ALERT type 1 CIF construction/syntax error, inconsistent or missing data
1 ALERT type 2 Indicator that the structure model may be wrong or deficient
4 ALERT type 3 Indicator that the structure quality may be low
3 ALERT type 4 Improvement, methodology, query or suggestion
2 ALERT type 5 Informative message, check

It is advisable to attempt to resolve as many as possible of the alerts in all categories. Often the minor alerts point to easily fixed oversights, errors and omissions in your CIF or refinement strategy, so attention to these fine details can be worthwhile. In order to resolve some of the more serious problems it may be necessary to carry out additional measurements or structure refinements. However, the purpose of your study may justify the reported deviations and the more serious of these should normally be commented upon in the discussion or experimental section of a paper or in the "special_details" fields of the CIF. checkCIF was carefully designed to identify outliers and unusual parameters, but every test has its limitations and alerts that are not important in a particular case may appear. Conversely, the absence of alerts does not guarantee there are no aspects of the results needing attention. It is up to the individual to critically assess their own results and, if necessary, seek expert advice.

Publication of your CIF in IUCr journals

A basic structural check has been run on your CIF. These basic checks will be run on all CIFs submitted for publication in IUCr journals (*Acta Crystallographica*, *Journal of Applied Crystallography*, *Journal of Synchrotron Radiation*); however, if you intend to submit to *Acta Crystallographica Section C* or *E* or *IUCrData*, you should make sure that full publication checks are run on the final version of your CIF prior to submission.

Publication of your CIF in other journals

Please refer to the *Notes for Authors* of the relevant journal for any special instructions relating to CIF submission.

PLATON version of 19/12/2024; check.def file version of 19/12/2024

Datablock B-O-S - ellipsoid plot

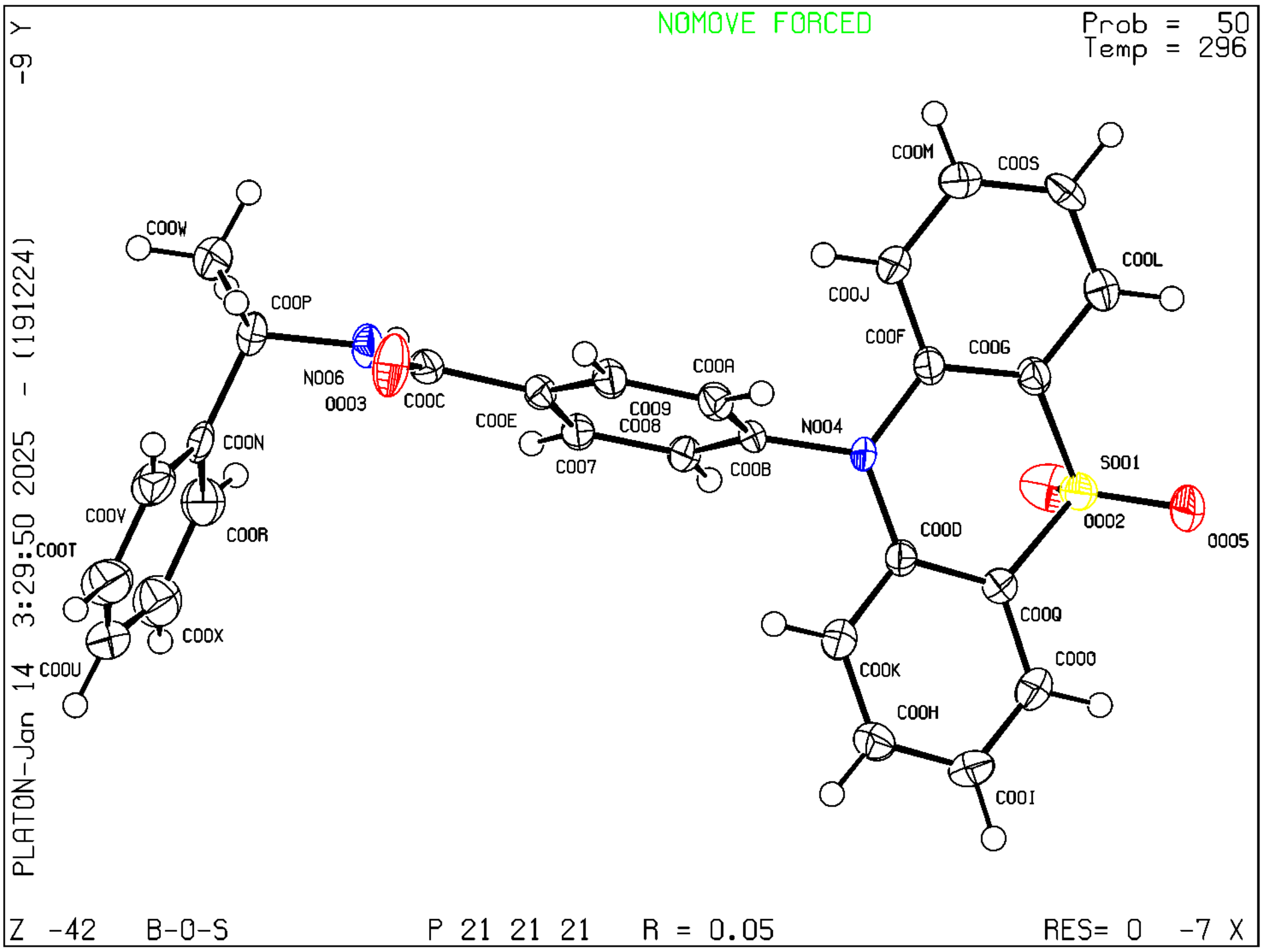

Supplement: Supplementary file 4 [file 41377_2026_2367_MOESM13_ESM.pdf]
