# Supplementary material for: A new platform for achieving long-term air-stable long persistent luminescence in amorphous organic system
Source: Light Sci Appl. 2026 Aug 18;15:344. doi: 10.1038/s41377-026-02367-6 (PMC13487193; doi:10.1038/s41377-026-02367-6)
Supplement: Supplementary file 5 [file 41377_2026_2367_MOESM1_ESM.pdf]

Supplementary Information for

A New Platform for Achieving Long-Term Air-Stable Long Persistent Luminescence in Amorphous Organic System

Zhenjiang Liu[1,+], Yunshu Meng[1,+], Zhihe Chi[1,2], Jia Ren[1], Ziang Song[1], Hui Zhang[1], Yu Tian[1], Yunsheng Wang[1], Jiaqiang Wang[1], Dan Li[1], Manman Fang[1], Zheng Zhao[3], Jie Yang[1,2,*], Ben Zhong Tang[1,3,*], Zhen Li[1,2,4,*]

[1]Institute of Molecular Aggregation Science, Tianjin University, Tianjin 300072, China.
[2]The International Joint Institute of Tianjin University, Fuzhou, Tianjin University, Tianjin 300072, China
[3]Guangdong Basic Research Center of Excellence for Aggregate Science, School of Science and Engineering, The Chinese University of Hong Kong, Shenzhen, Guangdong, 518172, China.
[4]Hubei Key Lab on Organic and Polymeric Opto-Electronic Materials, Department of Chemistry, Wuhan University, Wuhan, 430072, China.
*Correspondence: Jie Yang (jieyang2018@tju.edu.cn) or Ben Zhong Tang (tangbenz@cuhk.edu.cn) or Zhen Li (lizhen@whu.edu.cn)
[+]These authors contributed equally to this work.

Experimental section
Materials
TCB, BTCN, TPN, TFTPN, TCTPN were purchased from Bide Pharmatech Ltd. and purified by column chromatography. 2PtzO-nC (n = 5, 6, 7, 8) and PtzO-nC (n = 3, 4) were synthesized according to literatures of *Light Sci. Appl.* **11**, 142 (2022) and *Mater. Chem. Front.* **3**, 1391-1397 (2019).

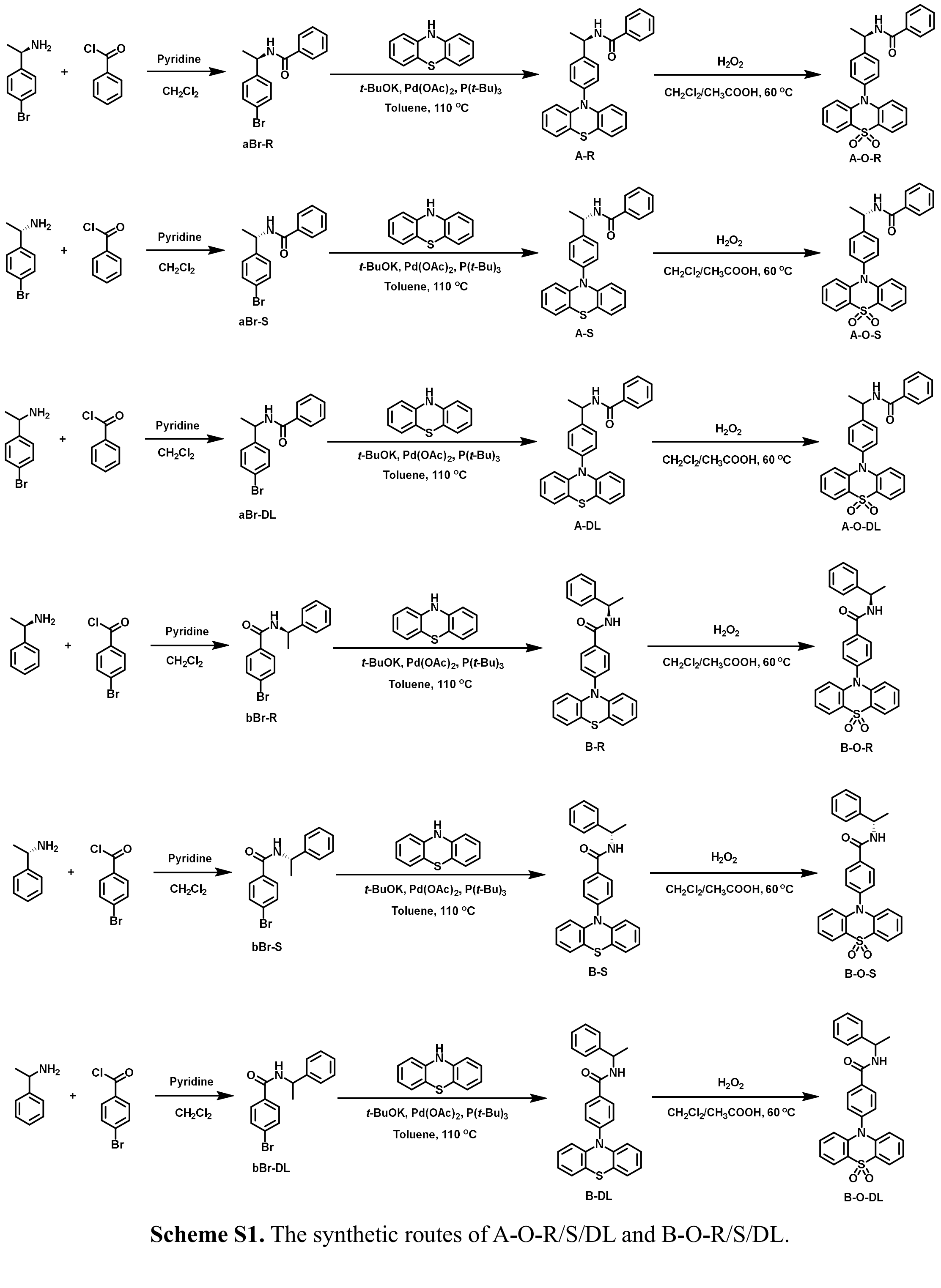

Scheme S1. The synthetic routes of A-O-R/S/DL and B-O-R/S/DL.

A-O-DL

1-(4-Bromophenyl)ethylamine (10 mmol, 2.00 g) was dissolved in DCM (50 mL), and benzoyl chloride (12 mmol, 1.68 g) was added dropwise at 0 °C. Then, add 1-2 mL of pyridine and allow the reaction to proceed at room temperature for 4 hours. Concentrate the reaction mixture to obtain a crude product, which is then purified by silica gel column chromatography to yield a white solid, aBr-DL.

^{1}H NMR (600 MHz, $CDCl_3$) δ 7.81 – 7.76 (m, 2H), 7.55 – 7.42 (m, 5H), 7.30 – 7.27 (m, 2H), 6.40 – 6.28 (m, 1H), 5.30 (p, J = 7.1 Hz, 1H), 1.60 (d, J = 6.9 Hz, 3H).

aBr-DL (2.53 g, 8 mmol), phenothiazine (1.50 g, 7.5 mmol), potassium tert-butoxide (1.12 g, 10 mmol), palladium acetate (0.11 g, 0.5 mmol), and a toluene solution of tri-tert-butylphosphine (0.5 mL, 0.25 mmol) were dissolved in 80 mL of anhydrous and oxygen-free toluene. Then, stir and reflux the mixtures under N_2 atmosphere for 12 hours. Cool the reaction mixture to room temperature and concentrate under reduced pressure. Purify the crude product by silica gel column chromatography (PE/DCM, 3:1, v/v) to obtain a white solid, A-DL.

^{1}H NMR (600 MHz, DMSO) δ 8.91 (d, *J* = 8.1 Hz, 1H), 7.95 (dd, *J* = 7.2, 1.7 Hz, 2H), 7.68 (d, *J* = 8.2 Hz, 2H), 7.56 (t, *J* = 7.3 Hz, 1H), 7.50 (t, *J* = 7.6 Hz, 2H), 7.39 (d, *J* = 8.3 Hz, 2H), 7.07 (dd, *J* = 7.6, 1.5 Hz, 2H), 6.94 (td, *J* = 8.3, 7.8, 1.6 Hz, 2H), 6.86 (td, *J* = 7.5, 1.2 Hz, 2H), 6.16 (dd, *J* = 8.3, 1.2 Hz, 2H), 5.33 (t, *J* = 7.3 Hz, 1H), 1.58 (d, *J* = 7.1 Hz, 3H).

The collected A-DL was dissolved in the mixtures of DCM (90 mL), acetic acid (20 mL), and H_2O_2 (5 mL). React at 60 °C for 24 hours. Extract the reaction mixture with DCM, then purify by column chromatography (PE/DCM, 2:1, v/v) to yield a white solid product, A-O-DL (2.21 g), with a final yield of 48.7%.

^{1}H NMR (600 MHz, $CDCl_3$): δ 8.12 (dd, J = 6.0, 1.8Hz, 2H), 7.85 (d, J = 8.0 Hz, 2H), 7.68 (d, J = 8.0, 2H), 7.53 (t, J = 6.4, J = 5.4, 1H), 7.46 (t, J = 7.6, 2H), 7.35 (m, 4H), 7.22 (t, J = 8.2, 2H), 6.60 (d, J = 8.4, 2H), 6.55 (d, J = 6.0 Hz, 2H), 5.48 (m, 1H), 1.71 (d, J = 6.4 Hz, 3H). ^{13}C NMR (151 MHz, $CDCl_3$): δ 166.88, 140.80, 137.84, 134.22, 132.89, 131.94, 130.74, 129.12, 128.82, 127.09, 123.45, 122.57, 122.17, 117.36, 100.00, 48.98, 22.36. HRMS (ESI), m/z: $[M+H]^+$ calcd. for $C_{27}H_{22}N_2O_3S_2$, 454.14239; found, 454.14199.

A-O-S

(S)-(-)-1-(4-bromophenyl)ethylamine (10 mmol, 2.00 g) was dissolved in DCM (50 mL), and benzoyl chloride (12 mmol, 1.68 g) was added dropwise at 0 °C. Then, add 1-2 mL of pyridine and allow the reaction to proceed at room temperature for 4 hours. Concentrate the reaction mixture to obtain a crude product, which is then purified by silica gel column chromatography to yield a white solid, aBr-S.

^{1}H NMR (600 MHz, $CDCl_3$) δ 7.58 – 7.54 (m, 2H), 7.32 – 7.18 (m, 5H), 7.05 (d, J = 8.2 Hz, 2H), 6.28 (d, J = 7.6 Hz, 1H), 5.06 (p, J = 7.1 Hz, 1H), 1.36 (d, J = 6.9 Hz, 3H).

aBr-S (2.53 g, 8 mmol), phenothiazine (1.50 g, 7.5 mmol), potassium tert-butoxide (1.12 g, 10 mmol), palladium acetate (0.11 g, 0.5 mmol), and a toluene solution of tri-tert-butylphosphine (0.5 mL, 0.25 mmol) were dissolved in 80 mL of anhydrous and oxygen-free toluene. Then, stir and reflux the mixtures under N_2 atmosphere for 12 hours. Cool the reaction mixture to room temperature and concentrate under reduced pressure. Purify the crude product by silica gel column chromatography (PE/DCM, 3:1, v/v) to obtain a white solid, A-S.

^{1}H NMR (600 MHz, DMSO) δ 8.96 (d, *J* = 8.1 Hz, 1H), 8.00 (dt, *J* = 8.2, 1.2 Hz, 2H), 7.74 (d, *J* = 8.0 Hz, 2H), 7.64 – 7.59 (m, 1H), 7.57 – 7.53 (m, 2H), 7.44 (d, *J* = 7.9 Hz, 2H), 7.15 – 7.11 (m, 2H), 6.99 (t, *J* = 7.8 Hz, 2H), 6.91 (t, *J* = 7.4 Hz, 2H), 6.24 – 6.20 (m, 2H), 5.38 (q, *J* = 7.3 Hz, 1H), 1.63 (d, *J* = 7.0 Hz, 3H).

The collected A-S was dissolved in the mixtures of DCM (90 mL), acetic acid (20 mL), and H_2O_2 (5 mL). React at 60 °C for 24 hours. Extract the reaction mixture with DCM, then further purify by column chromatography (PE/DCM, 2:1, v/v) to yield a white solid product, A-O-DL (2.37 g), with a final yield of 52.36%.

^{1}H NMR (600 MHz, $CDCl_3$): δ 8.11 (dd, J = 6.8, 1.8Hz, 2H), 7.85 (d, J = 8.0 Hz, 2H), 7.64 (d, J = 8.0, 2H), 7.58 (t, J = 6.4, J = 5.4, 1H), 7.48 (t, J = 7.2, 2H), 7.33 (m, 4H), 7.24 (t, J = 6.8, 2H), 6.68

(d, J = 7.6, 2H), 6.50 (d, J = 6.0 Hz, 2H), 5.44 (m, 1H), 1.66 (d, J = 6.0 Hz, 3H). ^{13}C NMR (151 MHz, $CDCl_3$): δ 166.84, 141.80, 133.86, 134.49, 131.75, 130.84, 130.01, 129.09, 128.64, 127.35, 125.65, 123.80, 121.34, 116.36, 109.95, 48.98, 22.37. HRMS (ESI), m/z: $[M+H]^+$ calcd. for $C_{27}H_{22}N_2O_3S_2$, 454.14239; found, 454.14219.

A-O-R

(R)-(+)-1-(4-bromophenyl)ethylamine (10 mmol, 2.00 g) was dissolve in DCM (50 mL), and benzoyl chloride (12 mmol, 1.68 g) was added dropwise at 0 °C. Then add 1-2 mL of pyridine and allow the reaction to proceed at room temperature for 4 hours. Concentrate the reaction mixture to obtain a crude product, which is then purified by silica gel column chromatography to yield a white solid, aBr-R.

^{1}H NMR (600 MHz, $CDCl_3$) δ 7.55 – 7.51 (m, 2H), 7.30 – 7.16 (m, 5H), 7.05 – 7.00 (m, 2H), 6.17 (d, J = 7.7 Hz, 1H), 5.04 (p, J = 7.0 Hz, 1H), 1.34 (d, J = 6.9 Hz, 3H).

aBr-R (2.53 g, 8 mmol), phenothiazine (1.50 g, 7.5 mmol), potassium tert-butoxide (1.12 g, 10 mmol), palladium acetate (0.11 g, 0.5 mmol), and a toluene solution of tri-tert-butylphosphine (0.5 mL, 0.25 mmol) were dissolved in 80 mL of anhydrous and oxygen-free toluene. Then, stir and reflux the mixtures under N_2 atmosphere for 12 hours. Cool the reaction mixture to room temperature and concentrate under reduced pressure. Purify the crude product by silica gel column chromatography (PE/DCM, 3:1, v/v) to obtain a white solid A-R.

^{1}H NMR (600 MHz, $CDCl_3$) δ 7.83 (d, J = 7.6 Hz, 2H), 7.61 (d, J = 7.8 Hz, 2H), 7.52 (dd, J = 8.5, 6.3 Hz, 1H), 7.45 (td, J = 7.7, 3.0 Hz, 2H), 7.35 (d, J = 7.9 Hz, 2H), 7.02 (d, J = 7.4 Hz, 2H), 6.89 – 6.77 (m, 2H), 6.51 (dt, J = 29.6, 7.9 Hz, 1H), 6.21 (s, 2H), 5.46 (p, J = 7.2 Hz, 1H), 1.68 (dd, J = 7.2, 2.4 Hz, 3H).

The collected A-R was dissolved in the mixtures of DCM (90 mL), acetic acid (20 mL), and H_2O_2 (5 mL). React at 60 °C for 24 hours. Extract the reaction mixture with DCM, then further purify by column chromatography (PE/DCM, 2:1, v/v) to yield a white solid product, A-O-R (2.11 g) with a final yield of 46.88%.

^{1}H NMR (600 MHz, $CDCl_3$): δ 8.13 (dd, J = 6.4, 1.8Hz, 2H), 7.85 (d, J = 6.8 Hz, 2H), 7.68 (d, J = 8.0, 2H), 7.54 (t, J = 6.4, J = 5.4, 1H), 7.51 (t, J = 7.2, 2H), 7.35 (m, 4H), 7.24 (t, J = 6.4, 2H), 6.64 (d, J = 7.8, 2H), 6.52 (d, J = 6.4 Hz, 2H), 5.43 (m, 1H), 1.64 (d, J = 6.0 Hz, 3H). ^{13}C NMR (151 MHz, $CDCl_3$): δ 166.74, 141.65, 133.87, 134.49, 131.99, 130.80, 130.11, 129.09, 128.64, 127.35, 125.65, 123.82, 121.30, 116.33, 109.95, 48.97, 22.37. HRMS (ESI), m/z: $[M+H]^+$ calcd. for $C_{27}H_{22}N_2O_3S_2$, 454.14239; found, 454.14183.

B-O-DL

1-Phenylethan-1-amine (10 mmol, 1.21 g) was dissolved in DCM (50 mL) and the resultant solution was cooled to 0 °C. Gradually add 4-bromobenzoyl chloride (12 mmol, 2.63 g) dropwise, then add 1-2 mL of pyridine. Allow the reaction to proceed at room temperature for 4 hours. Concentrate the reaction mixture to obtain a crude product, which is then purified by silica gel column chromatography to yield a white solid, bBr-DL.

^{1}H NMR (600 MHz, $CDCl_3$) δ 7.66 (d, J = 8.4 Hz, 2H), 7.52 (ddd, J = 8.6, 7.3, 1.6 Hz, 2H), 7.38 – 7.32 (m, 4H), 7.29 (d, J = 2.2 Hz, 1H), 6.37 (d, J = 7.7 Hz, 1H), 5.03 (p, J = 7.1 Hz, 1H), 1.45 (d, J = 7.1 Hz, 3H).

bBr-DL (2.53 g, 8 mmol), phenothiazine (1.50 g, 7.5 mmol), potassium tert-butoxide (1.12 g, 10 mmol), palladium acetate (0.11 g, 0.5 mmol), and a toluene solution of tri-tert-butylphosphine (0.5 mL, 0.25 mmol) were dissolved in 80 mL of dry and oxygen-free toluene. Stir and reflux the mixtures under nitrogen for 12 hours. Cool the reaction mixture to room temperature and concentrate it under reduced pressure. Purify the crude product by silica gel column chromatography (DCM, 3:1, v/v) to obtain B-DL as a white solid.

^{1}H NMR (600 MHz, DMSO) δ 8.89 (d, J = 8.0 Hz, 1H), 8.10 (d, J = 8.2 Hz, 2H), 7.46 – 7.40 (m, 4H), 7.34 (td, J = 7.7, 1.6 Hz, 2H), 7.24 (td, J = 7.3, 1.5 Hz, 1H), 7.18 (dd, J = 7.7, 1.6 Hz, 2H), 7.03 (td, J = 7.7, 1.6 Hz, 2H), 6.98 – 6.94 (m, 2H), 6.41 (dd, J = 8.1, 1.5 Hz, 2H), 5.21 (p, J = 7.3 Hz, 1H), 1.51 (d, J = 7.1 Hz, 3H).

The collected B-DL was dissolved in the mixtures of DCM (90 mL), acetic acid (20 mL), and H_2O_2 (5 mL). React at 60 °C for 24 hours. Extract the reaction mixture with DCM and further purify by column chromatography (PE/DCM, 2:1, v/v) to obtain 2.368 g of white solid product B-O-DL, with a final yield of 65.18%.

^{1}H NMR (600 MHz, $CDCl_3$): δ 8.15 (dd, J = 6.0, 1.8Hz, 2H), 8.08 (d, J = 8.4 Hz, 2H), 7.44 (m, 4H), 7.40-7.35 (m, 4H), 7.31 (m, 1H), 7.25 (m, 2H), 6.55 (d, J = 8.4 Hz, 2H), 6.48 (d, J = 7.8 Hz, 1H), 5.39 (m, 1H), 1.67 (d, J = 6.6 Hz, 3H). ^{13}C NMR (150 MHz, $CDCl_3$) δ 142.78, 141.66, 140.48, 136.19, 132.97, 131.03, 130.19, 128.99, 127.84, 126.40, 123.63, 122.85, 122.45, 117.05, 49.72, 21.71. HRMS (ESI), m/z: $[M+H]^+$ calcd. for $C_{27}H_{22}N_2O_3S_2$, 454.14239; found, 454.14117.

B-O-S

(S)-(-)-1-phenylethan-1-amine (10 mmol, 1.21 g) was dissolved in DCM (50 mL) and the resultant solution was cooled to 0 °C. Gradually add 4-bromobenzoyl chloride (12 mmol, 2.63 g) dropwise, then add 1-2 mL of pyridine. Allow the reaction to proceed at room temperature for 4 hours. Concentrate the reaction mixture to obtain a crude product, which is then purified by silica gel column chromatography to yield a white solid, bBr-S.

^{1}H NMR (600 MHz, $CDCl_3$) δ 7.62 (d, J = 8.2 Hz, 2H), 7.53 (d, J = 8.1 Hz, 2H), 7.40 – 7.32 (m, 4H), 7.28 (t, J = 6.7 Hz, 1H), 6.43 (d, J = 7.5 Hz, 1H), 5.31 (q, J = 7.0 Hz, 1H), 1.59 (d, J = 6.8 Hz, 3H).

bBr-S (2.53 g, 8 mmol), phenothiazine (1.50 g, 7.5 mmol), potassium tert-butoxide (1.12 g, 10 mmol), palladium acetate (0.11 g, 0.5 mmol), and a toluene solution of tri-tert-butylphosphine (0.5 mL, 0.25 mmol) were dissolved in 80 mL of dry and oxygen-free toluene. Stir and reflux the mixtures under nitrogen for 12 hours. Cool the reaction mixture to room temperature and concentrate it under reduced pressure. Purify the crude product by silica gel column chromatography (DCM, 3:1, v/v) to obtain B-S as a white solid.

^{1}H NMR (600 MHz, DMSO) δ 8.89 (d, J = 8.0 Hz, 1H), 8.12 – 8.08 (m, 2H), 7.43 (dd, J = 8.6, 6.7 Hz, 4H), 7.34 (t, J = 7.7 Hz, 2H), 7.26 – 7.21 (m, 1H), 7.18 (dd, J = 7.6, 1.6 Hz, 2H), 7.03 (td, J = 7.7, 1.6 Hz, 2H), 6.96 (td, J = 7.4, 1.3 Hz, 2H), 6.41 (dd, J = 8.2, 1.2 Hz, 2H), 5.21 (q, J = 7.3 Hz, 1H), 1.51 (d, J = 7.1 Hz, 3H).

The collected B-S was dissolved in the mixtures of DCM (90 mL), acetic acid (20 mL), and H_2O_2 (5 mL). React at 60 °C for 24 hours. Extract the reaction mixture with DCM and further purify by column chromatography (PE/DCM, 2:1, v/v) to obtain 2.298 g of white solid product B-O-S, with a final yield of 63.18%. ^{1}H NMR (600 MHz, $CDCl_3$): δ 8.14 (dd, J = 6.4, 1.8Hz, 2H), 8.07 (d, J = 8.0 Hz, 2H), 7.44 (m, 4H), 7.40-7.36 (m, 4H), 7.31 (m, 1H), 7.24 (m, 2H), 6.54 (d, J = 8.4 Hz, 2H), 6.46 (d, J = 7.4 Hz, 1H), 5.39 (m, 1H), 1.66 (d, J = 6.6 Hz, 3H). ^{13}C NMR (150 MHz, $CDCl_3$) δ 160.80, 143.57, 141.58, 140.40, 136.20, 132.95, 130.55, 130.21, 128.93, 127.79, 126.21, 123.52, 122.84, 123.46, 117.08, 49.71, 21.74. HRMS (ESI), m/z: $[M+H]^+$ calcd. for $C_{27}H_{22}N_2O_3S_2$, 454.14239; found, 454.14157.

B-O-R

(R)-(+)-1-phenylethan-1-amine (10 mmol, 1.21 g) was dissolved in DCM (50 mL) and the resultant solution was cooled to 0 °C. Gradually add 4-bromobenzoyl chloride (12 mmol, 2.63 g) dropwise, then add 1-2 mL of pyridine. Allow the reaction to proceed at room temperature for 4 hours. Concentrate the reaction mixture to obtain a crude product, which is then purified by silica gel column chromatography to yield a white solid, bBr-R.

^{1}H NMR (600 MHz, $CDCl_3$) δ 7.56 – 7.52 (m, 2H), 7.46 – 7.42 (m, 2H), 7.32 – 7.25 (m, 4H), 7.22 – 7.19 (m, 1H), 6.51 (d, J = 7.8 Hz, 1H), 5.21 (p, J = 7.1 Hz, 1H), 1.51 (d, J = 6.9 Hz, 3H).

bBr-R (2.53 g, 8 mmol), phenothiazine (1.50 g, 7.5 mmol), potassium tert-butoxide (1.12 g, 10 mmol), palladium acetate (0.11 g, 0.5 mmol), and a toluene solution of tri-tert-butylphosphine (0.5 mL, 0.25 mmol) were dissolved in 80 mL of dry and oxygen-free toluene. Stir and reflux the mixtures under nitrogen for 12 hours. Cool the reaction mixture to room temperature and concentrate it under reduced pressure. Purify the crude product by silica gel column chromatography (DCM, 3:1, v/v) to obtain B-R as a white solid.

^{1}H NMR (600 MHz, $CDCl_3$) δ 8.89 (d, J = 8.0 Hz, 1H), 8.13 – 8.08 (m, 2H), 7.47 – 7.39 (m, 4H),

7.34 (t, J = 7.7 Hz, 2H), 7.26 – 7.23 (m, 1H), 7.18 (dd, J = 7.6, 1.6 Hz, 2H), 7.05 – 7.01 (m, 2H), 6.96 (td, J = 7.4, 1.2 Hz, 2H), 6.41 (dd, J = 8.2, 1.2 Hz, 2H), 5.23 – 5.19 (m, 1H), 1.51 (d, J = 7.1 Hz, 3H).

The collected B-R was dissolved in the mixtures of DCM (90 mL), acetic acid (20 mL), and H_2O_2 (5 mL). React at 60 °C for 24 hours. Extract the reaction mixture with DCM and further purify by column chromatography (PE/DCM, 2:1, v/v) to obtain 2.74 g of white solid product B-O-R, with a final yield of 60.26%.
^{1}H NMR (600 MHz, $CDCl_3$): δ 8.14 (dd, J = 7.9, 1.6 Hz, 2H), 8.06 (d, J = 8.4 Hz, 2H), 7.44-7.33 (m, 8H), 7.30 (m, 1H), 7.24 (m, 2H), 6.60 (d, J = 7.8 Hz, 1H), 6.55 (d, J = 8.4 Hz, 2H), 5.39 (m, 1H), 1.66 (d, J = 7.2 Hz, 3H).
^{13}C NMR (150 MHz, $CDCl_3$) δ 165.40, 142.87, 141.58, 140.47, 136.20, 132.99, 130.95, 130.24, 128.96, 127.79, 126.41, 123.59, 122.80, 122.46, 117.08, 49.71, 21.72. HRMS (ESI), m/z: $[M+H]^+$ calcd. for $C_{27}H_{22}N_2O_3S_2$, 454.1423; found, 454.14025.

Results and discussion

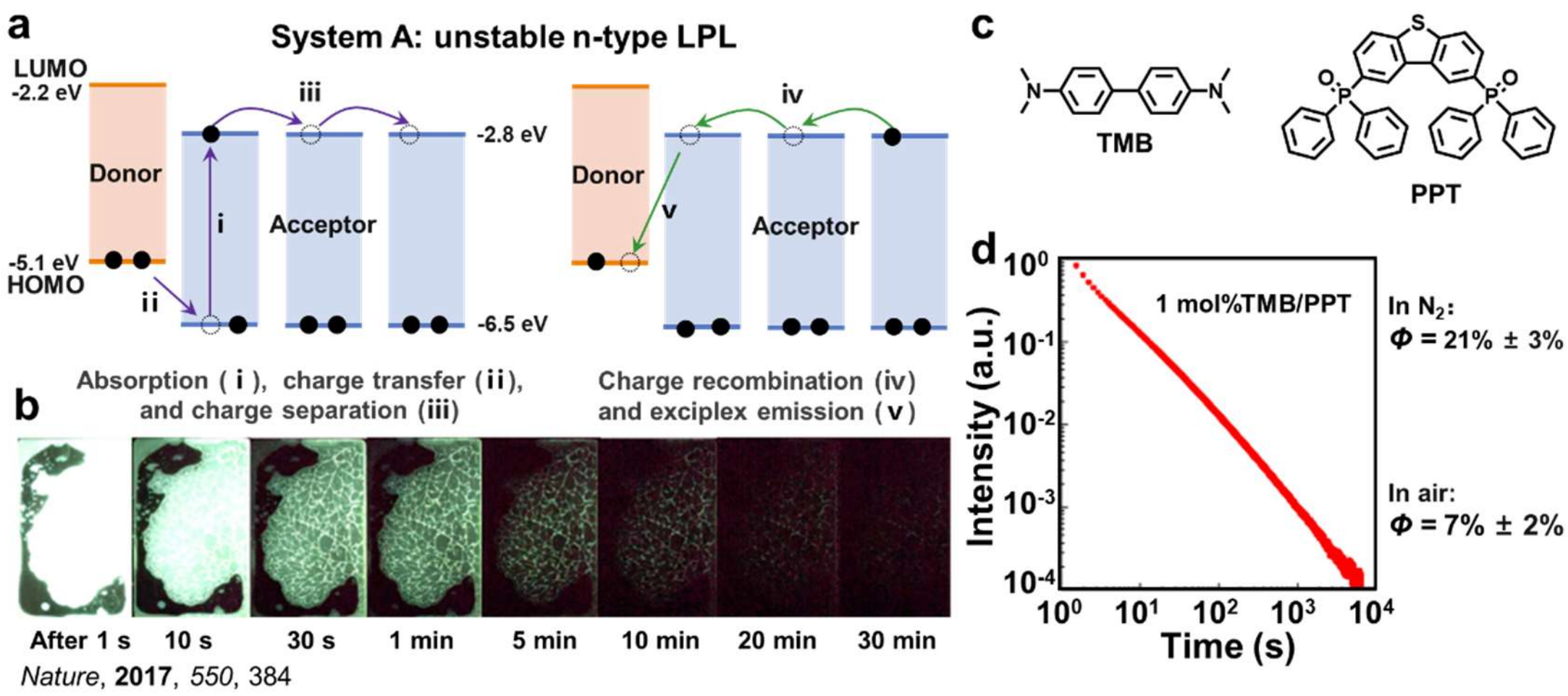

Chart S1. a) OLPL emission mechanism of n-type system. b) Photographs of OLPL from a 1 mol% TMB/PPT film. c) Chemical structure of TMB and PPT. d) Time-resolved PL decay curve of 1 mol% TMB/PPT film.

Note: TMB acted as electron-donor guest while PPT as electron-acceptor host. The PLQY of 1 mol% TMB/PPT film was 21% ± 3% in nitrogen and only 7% ± 2% in oxygen, exhibiting an unstable OLPL emission in air.

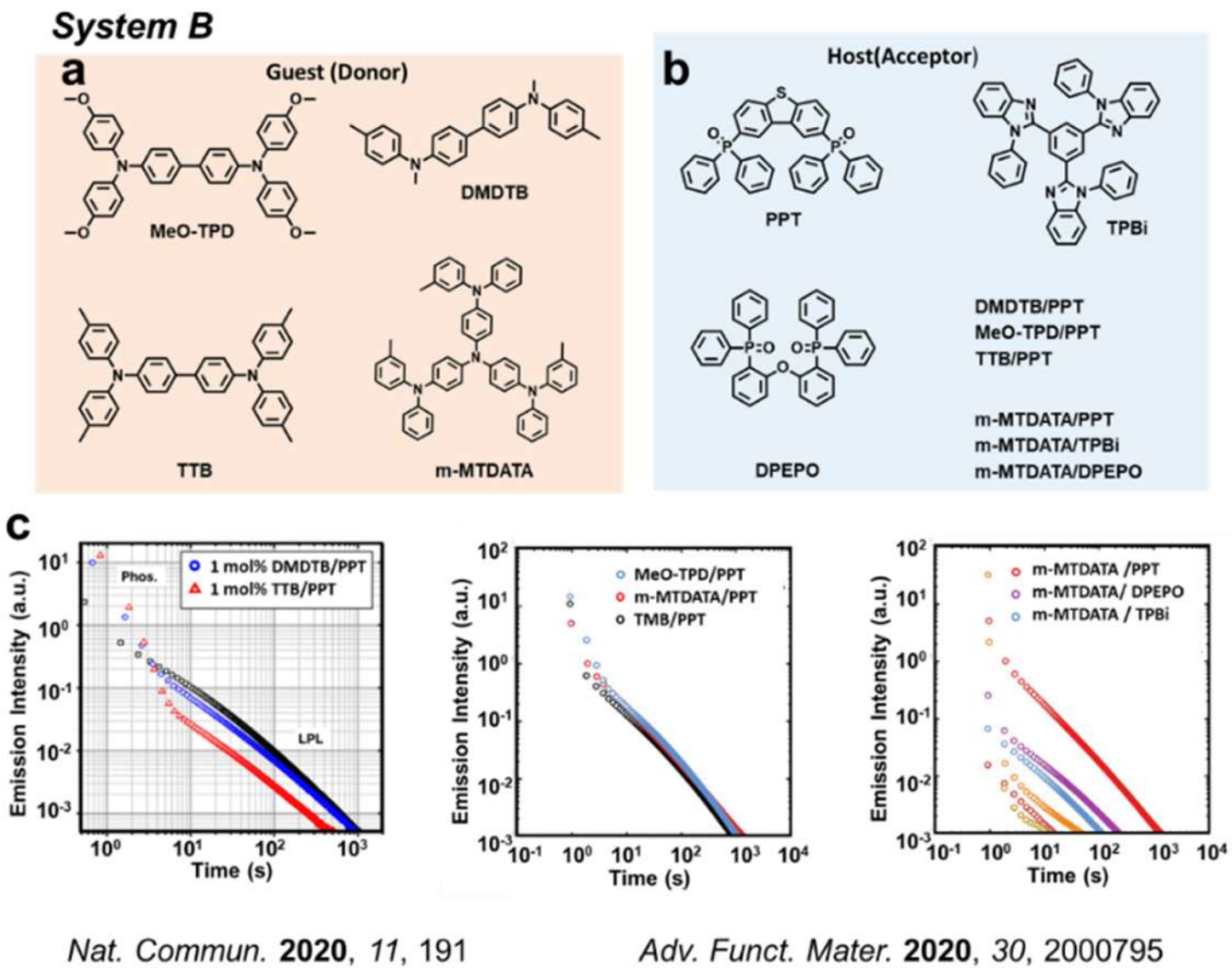

Chart S2. a) Chemical structure of guests. b) Chemical structure of hosts. c) Time-resolved OLPL decay curves of different exciplex.

Note: Exciplex formed by different donor and acceptor exhibited different OLPL emissions. For those systems, films were made and the corresponding optical properties were measured in nitrogen-filled glove.

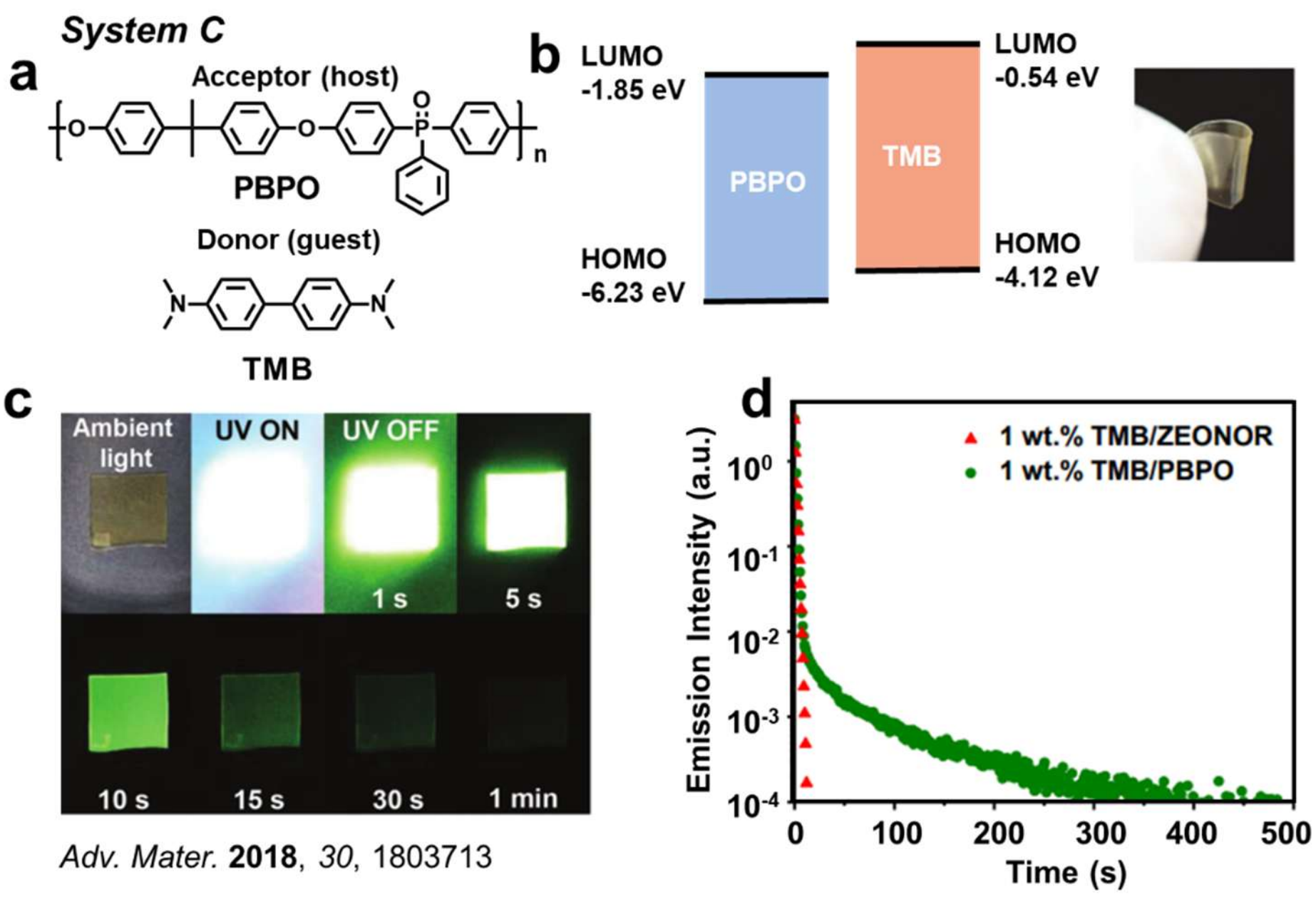

Chart S3. a) Chemical structures of guest and polymer host. b) The HOMO and LUMO of PBPO and TMB. c) Photographs of OLPL from this polymer system. d) Time-resolved PL decay curve of 1 mol% TMB/PBPO film.

Note: Similarly, film was made and corresponding optical properties were measured in nitrogen-filled glove for this system.

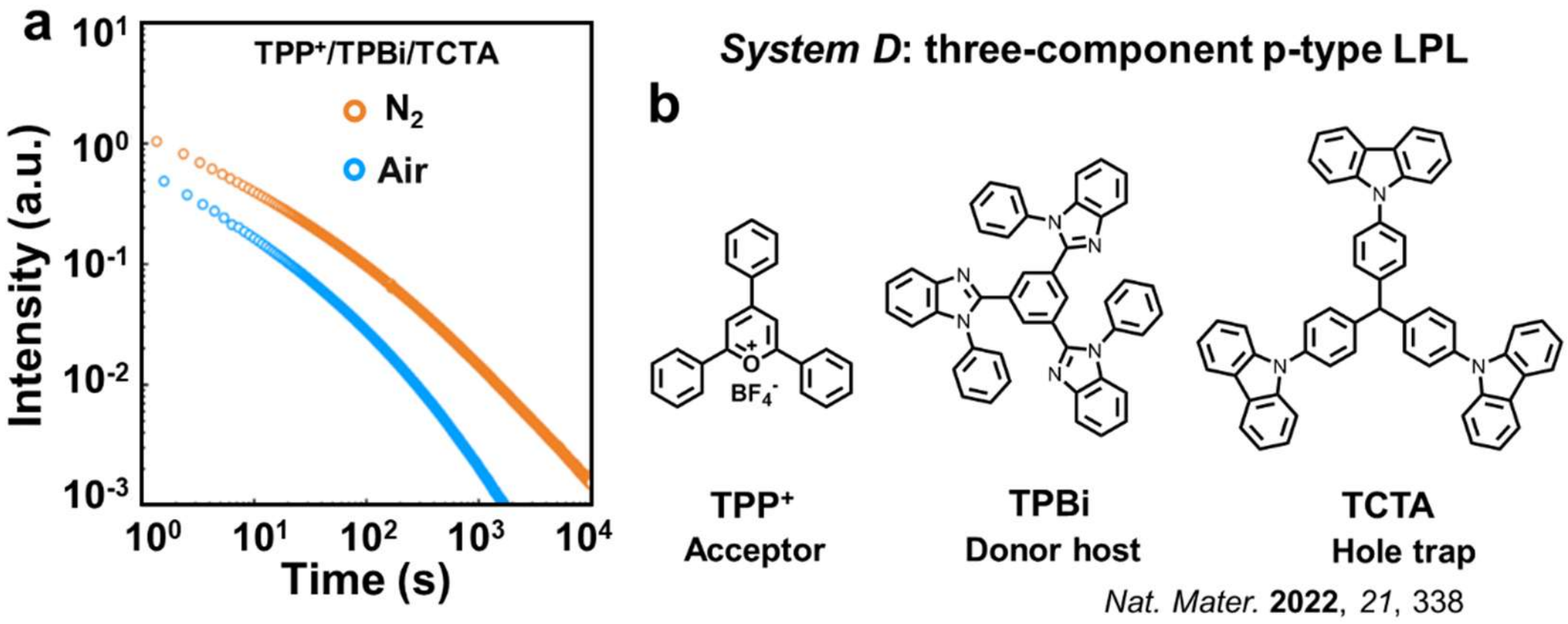

Chart S4. a) Time-resolved OLPL decay curves of TPP^+/TPBi/TCTA at different environments. b) Chemical structures of TPP^+, TPBi and TCTA.

Note: These results indicate that the p-type OLPL system with deep HOMO levels can emit OLPL in air but cannot prevent triplet quenching by oxygen. Future developments of low-oxygen-diffusion hosts or advanced encapsulation techniques to prevent oxygen are required to obtain efficient OLPL materials in air.

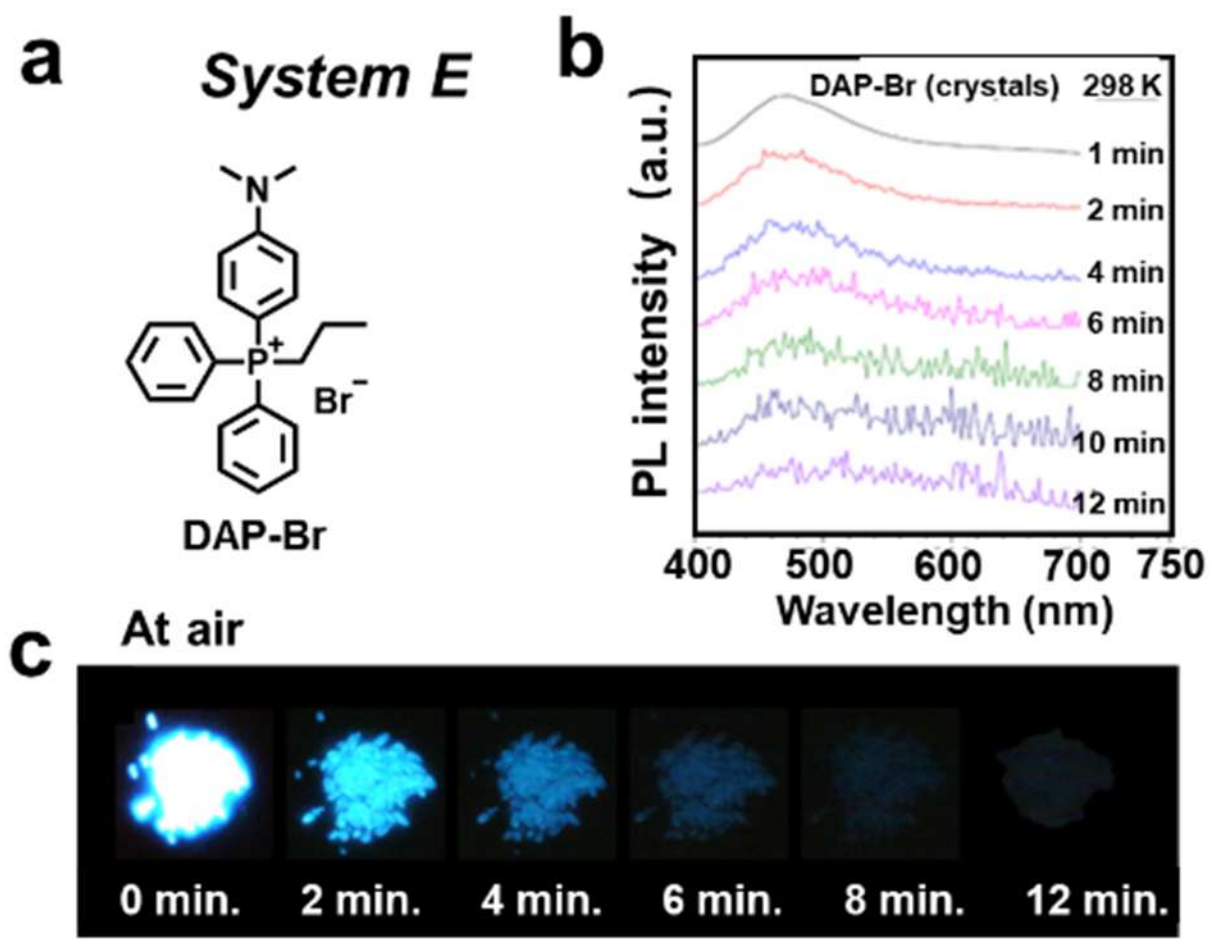

J. Am. Chem. Soc. **2022**, *144*, 3050

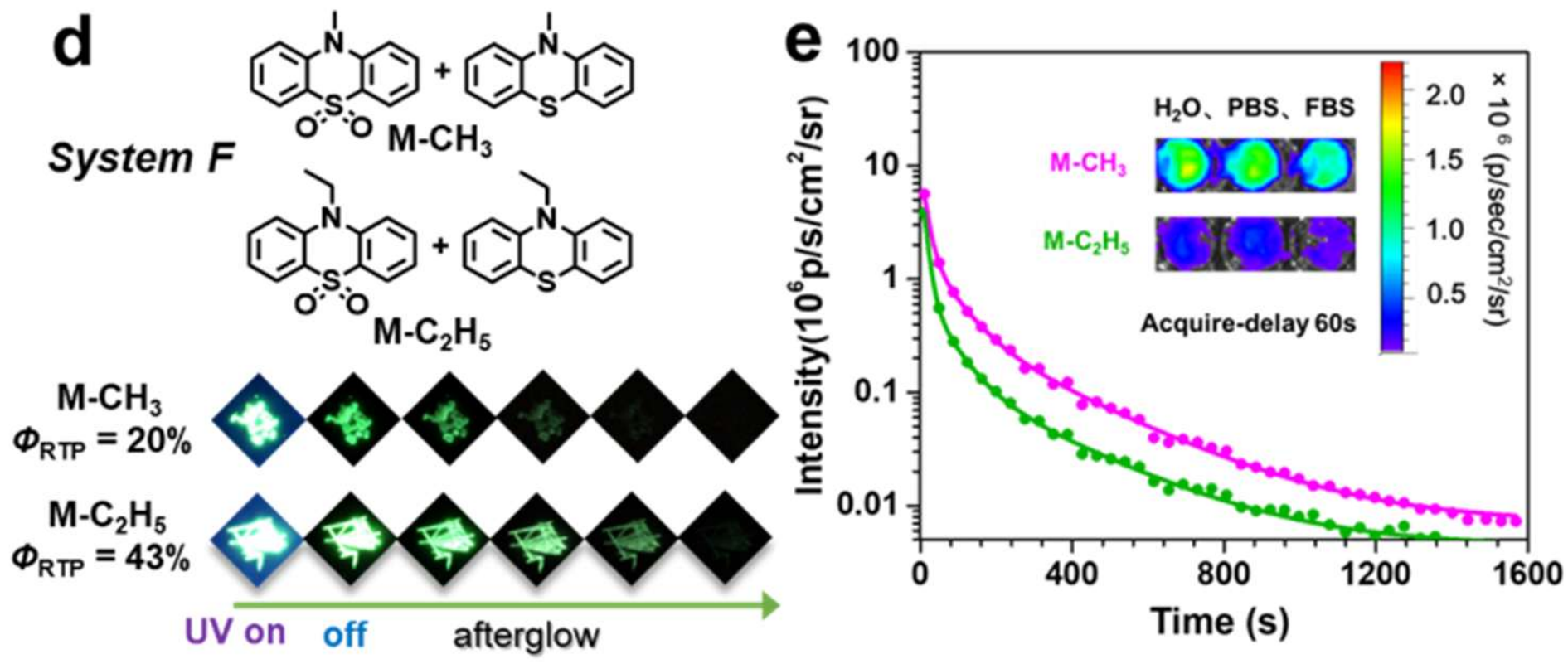

Adv. Mater. **2021**, *33*, 2007811

Chart S5. a) Chemical structure of DAP-Br. b) Emission spectra of DAP-Br crystals measured at different times (0−12 min) after the cessation of excitation at 365 nm. c) Photographs of DAP-Br crystals taken under 365 nm UV excitation and its subsequent afterglow. d) Chemical structures and photographs of M-CH_3 and M-C_2H_3. e) Time-resolved OLPL decay curves of M-CH_3 and M-C_2H_3 nanocrystals.

Notes: Although DAP-Br exhibited excellent OLPL performance at ambient condition, OLPL in the other phosphonium bromide derivatives was not observed. OLPL emission in System F was dependent on molecular packing of host. Thus, a systematic and complete model was urgently needed to reveal the relationship between molecular packing and air-stable OLPL.

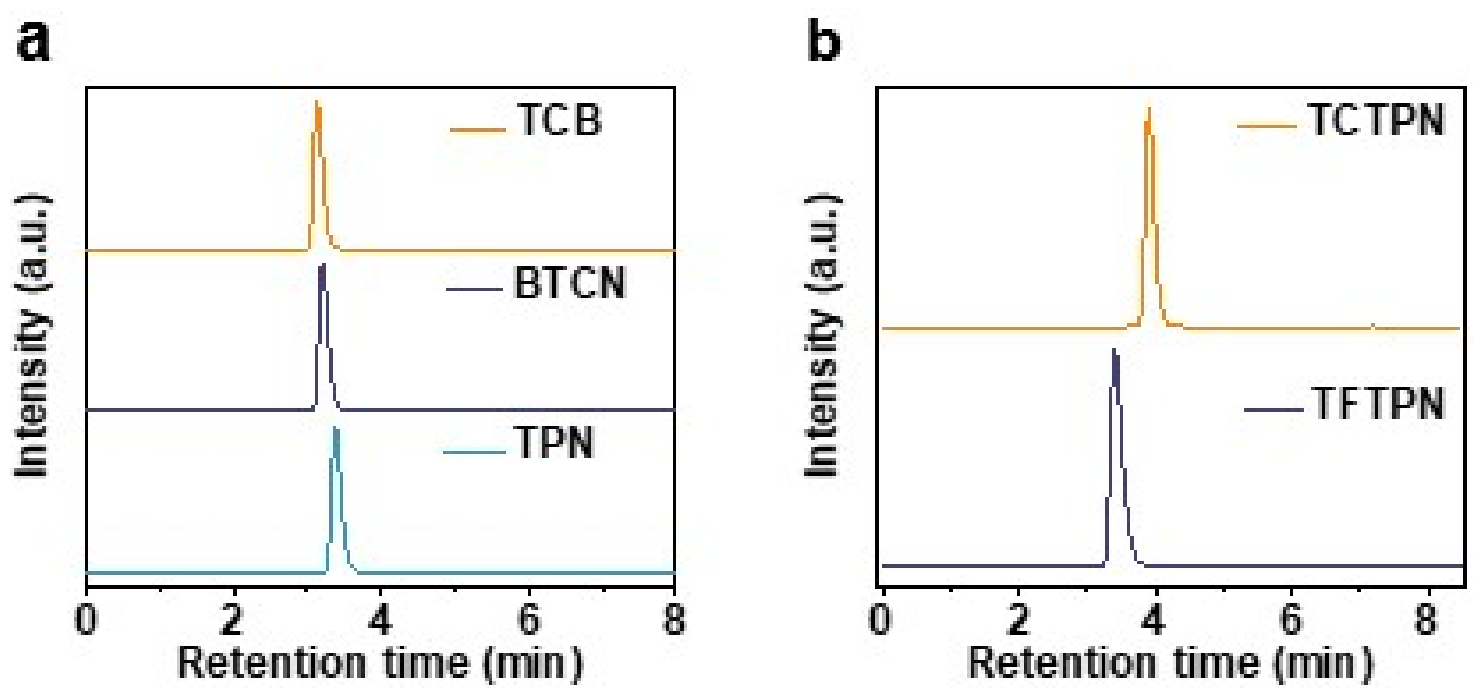

Fig. S1. High performance liquid chromatography diagrams of guest molecules.

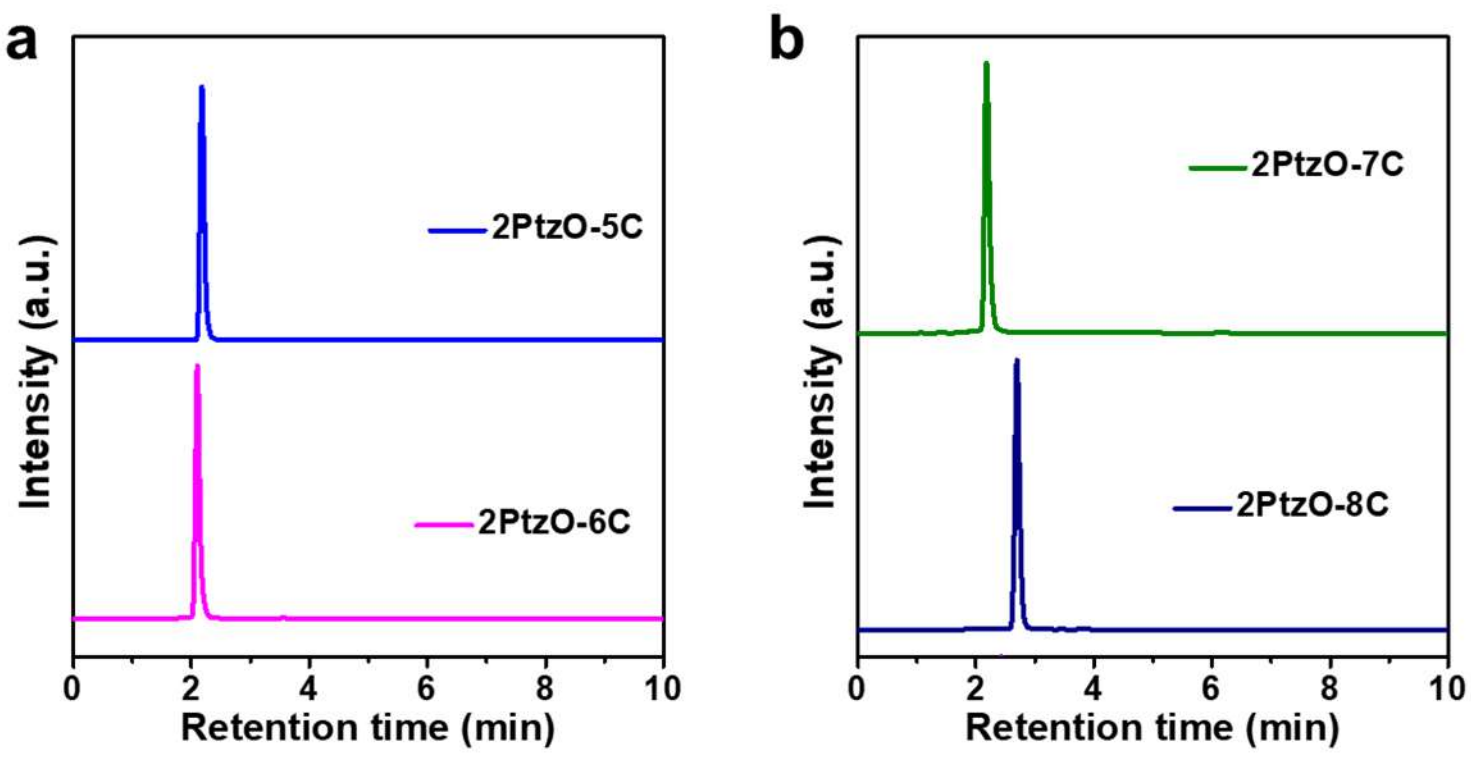

Fig. S2. a) High performance liquid chromatography diagrams of 2PtzO-5C and 2PtzO-6C. b) High performance liquid chromatography diagrams of 2PtzO-7C and 2PtzO-8C.

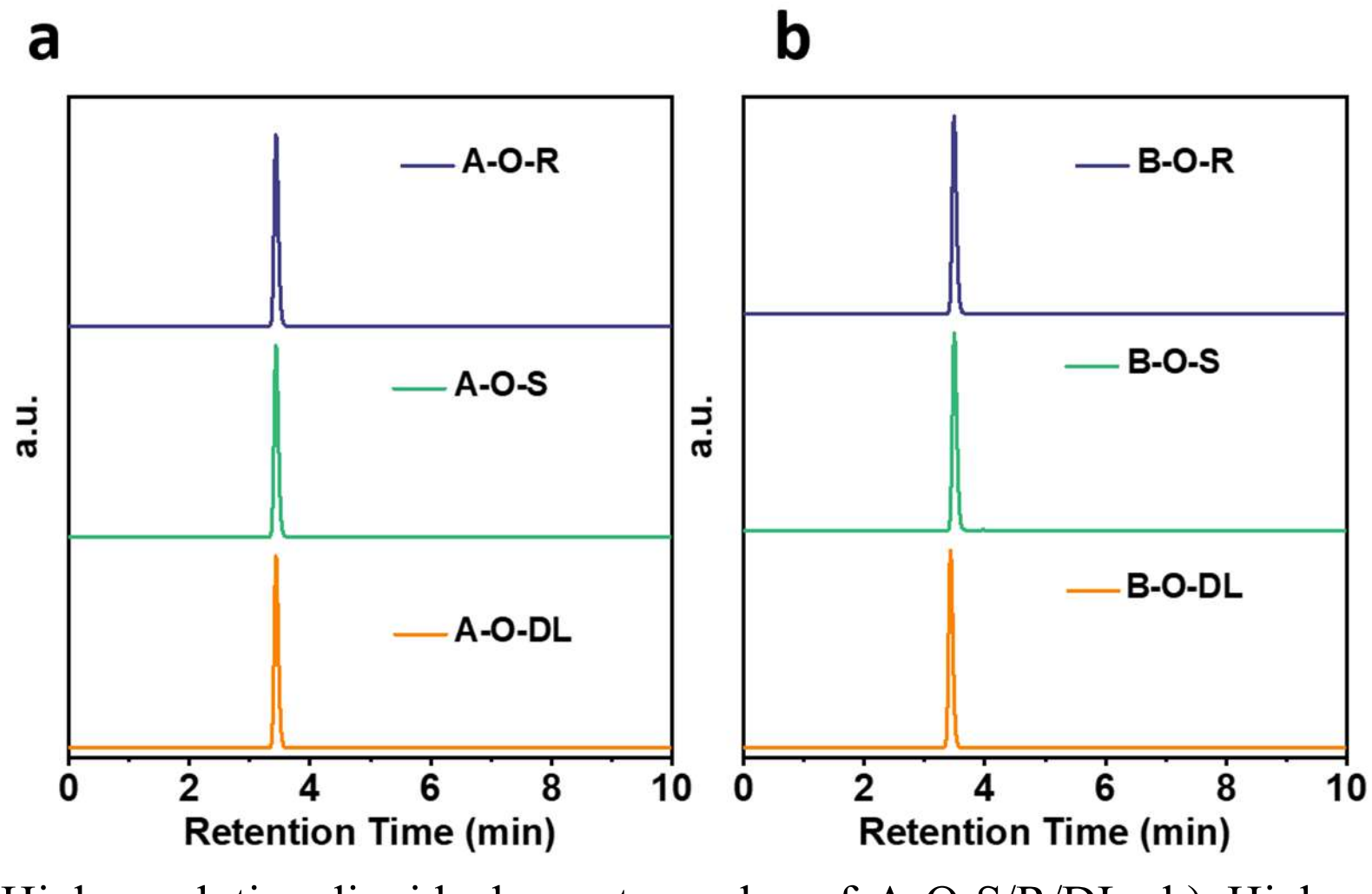

Fig. S3. a) High-resolution liquid chromatography of A-O-S/R/DL. b) High-resolution liquid chromatography of B-O-S/R/DL.

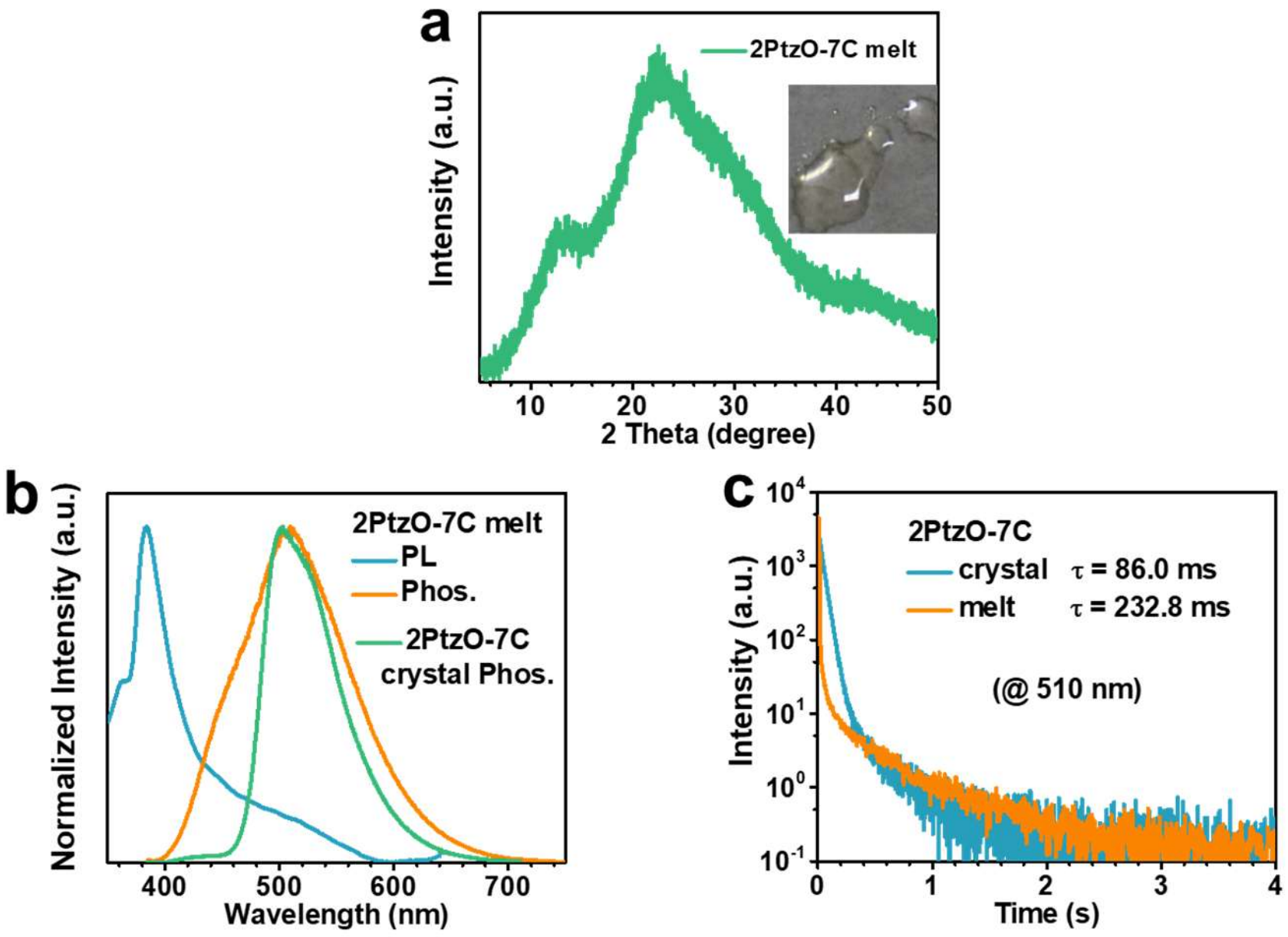

Fig. S4. a) PXRD pattern of 2PtzO-7C melt. Inset: corresponding picture under day light. b) Steady-state PL and phosphorescence spectra of 2PtzO-7C at different states. c) Time-resolved RTP-decay curves of 2PtzO-7C at different states.

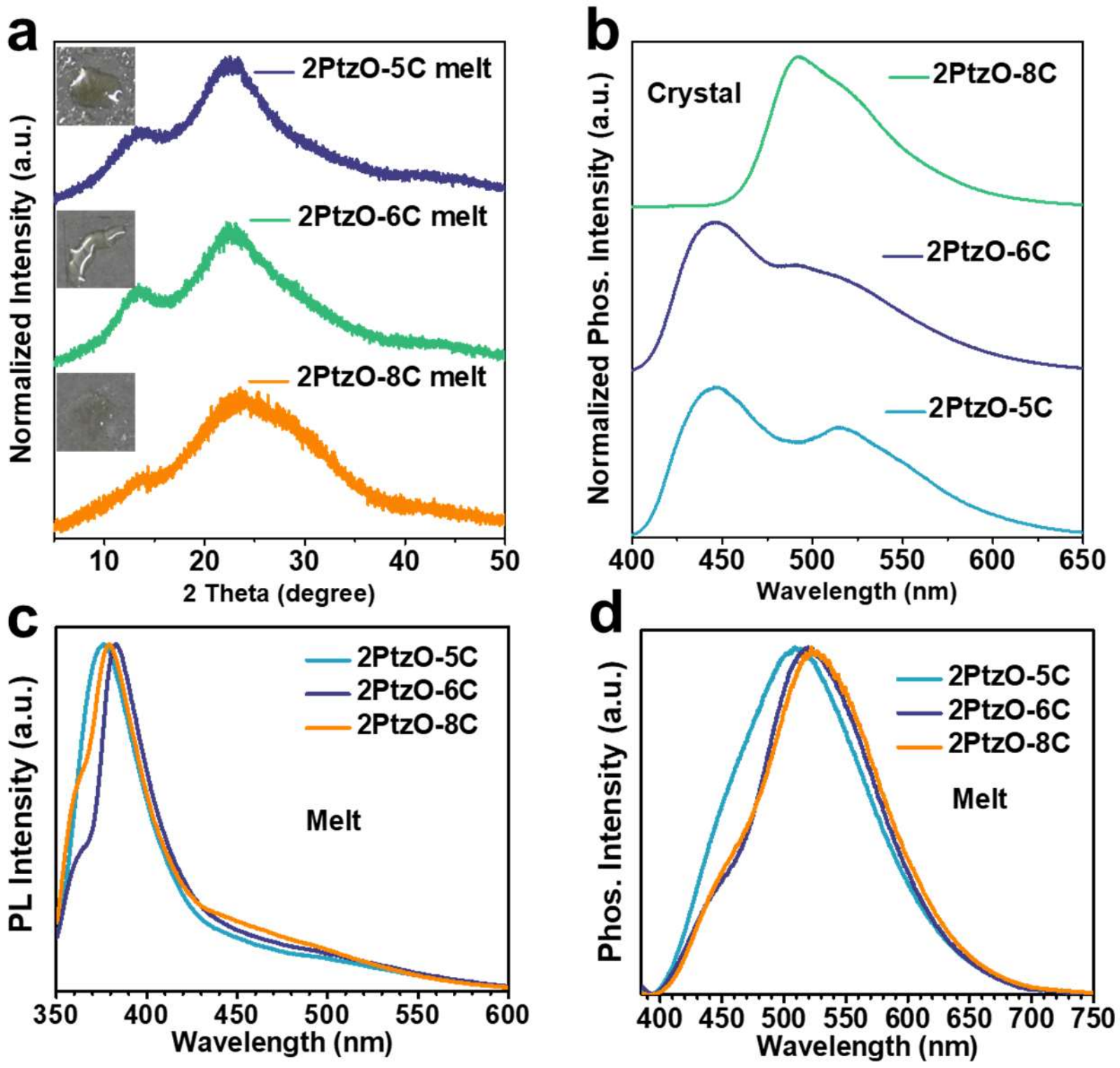

Fig. S5. a) PXRD patterns of 2PtzO-nC (n = 5, 6, 8) melts. Inset: corresponding pictures under day light. b) Phosphorescence spectra of 2PtzO-nC (n = 5, 6, 8) in crystal state (λ_{ex} = 365 nm). c) PL spectra of 2PtzO-nC (n = 5, 6, 8) in melting state (λ_{ex} = 310 nm). d) Phosphorescence spectra of 2PtzO-nC (n = 5, 6, 8) in melting state.

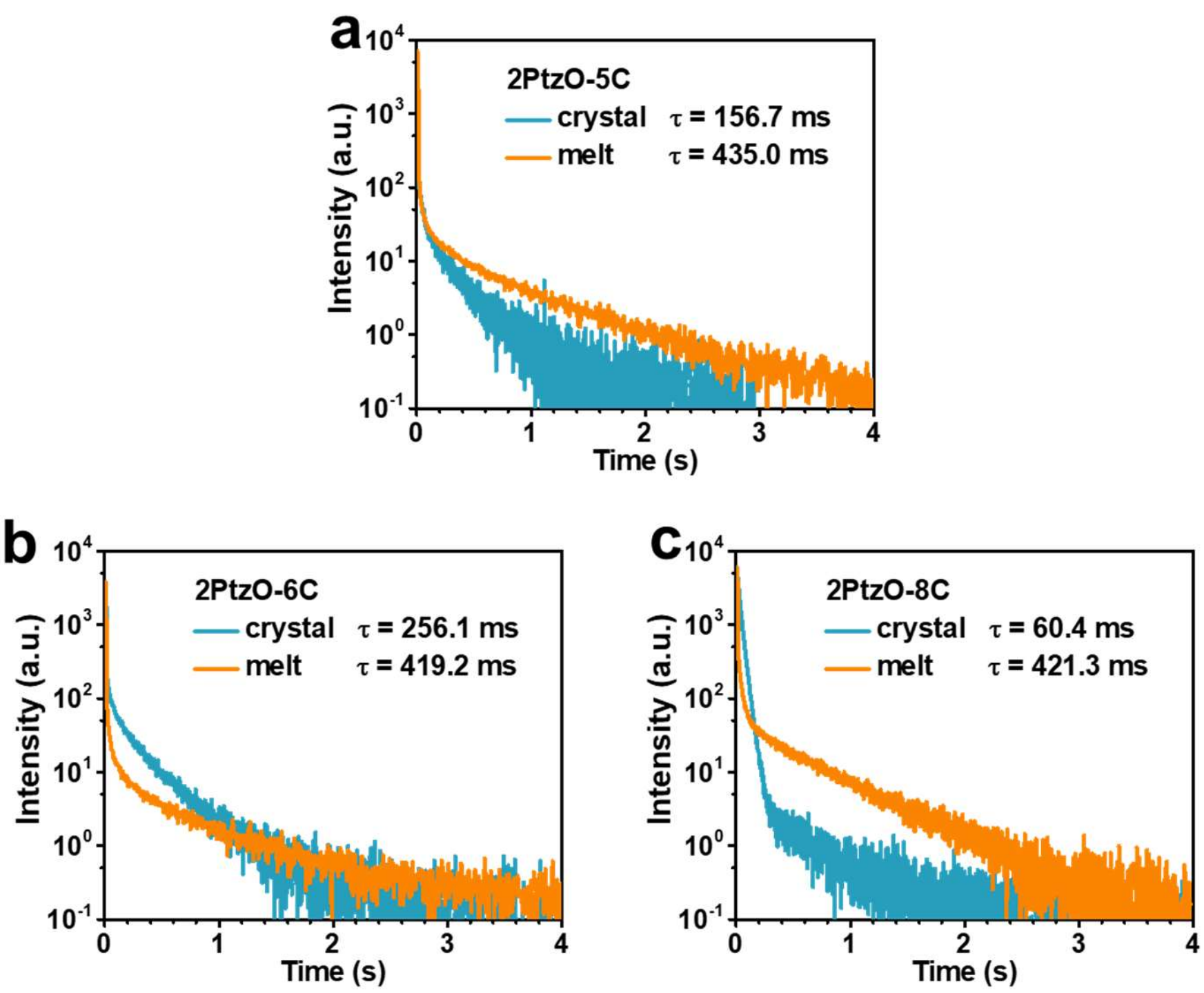

Fig. S6. Time-resolved RTP-decay curves of 2PtzO-nC (n = 5, 6, 8) at different states.

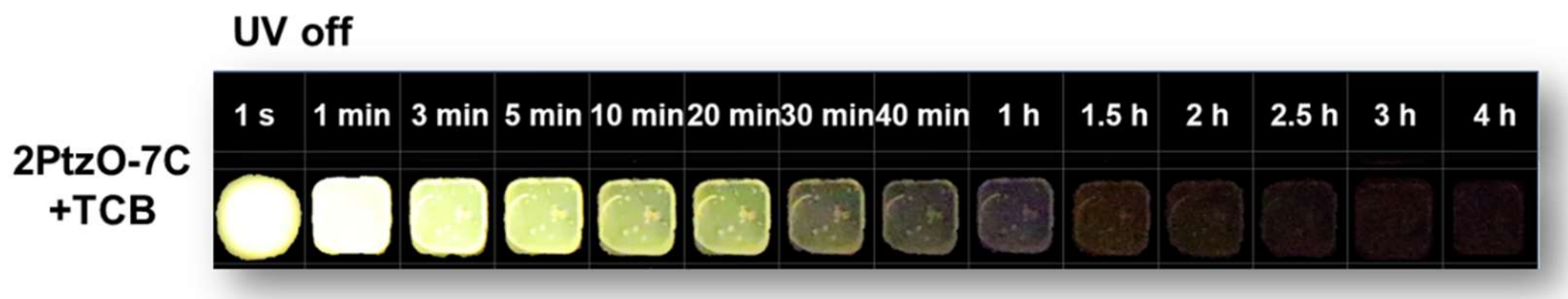

Fig. S7. Photographs of 2PtzO-7C+TCB taken under air by Canon camera after the removal of 365 nm UV lamp for different times.

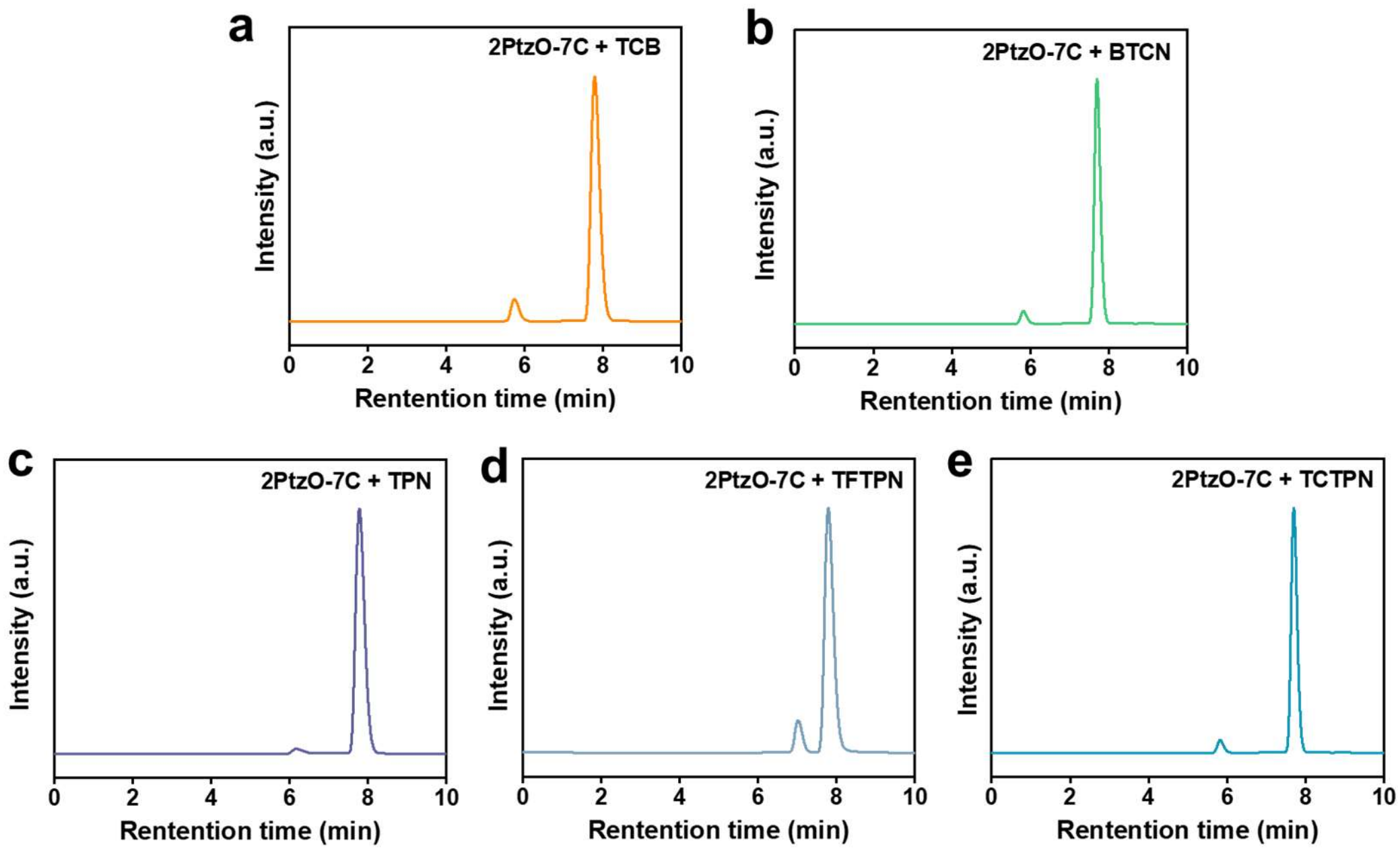

Fig. S8. HPLC curves of 2PtzO-7C doped with different guests (TCB, BTCN, TPN, TFTPN, TCTPN) after UV excitation.

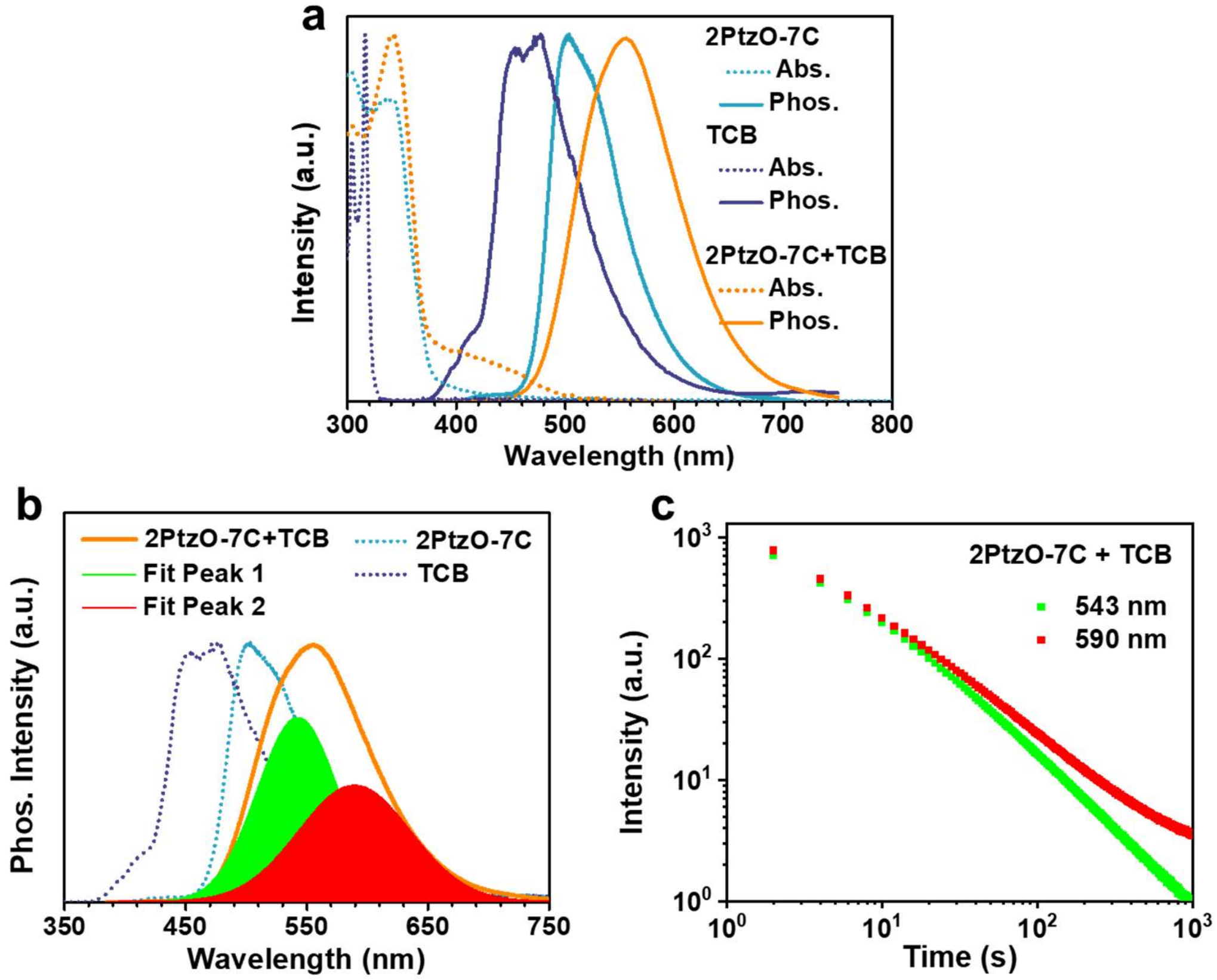

Fig. S9. a) Comparison of phosphorescence and absorption spectra of TCB, 2PtzO-7C, and 2PtzO-7C+TCB. b) Afterglow emission spectra of 2PtzO-7C+TCB (one original and two fitted), 2PtzO-7C in solid state at room temperature, and TCB in DCM solution at 77 K; c) Time-resolved OLPL-decay curves of 2PtzO-7C+TCB for different bands.

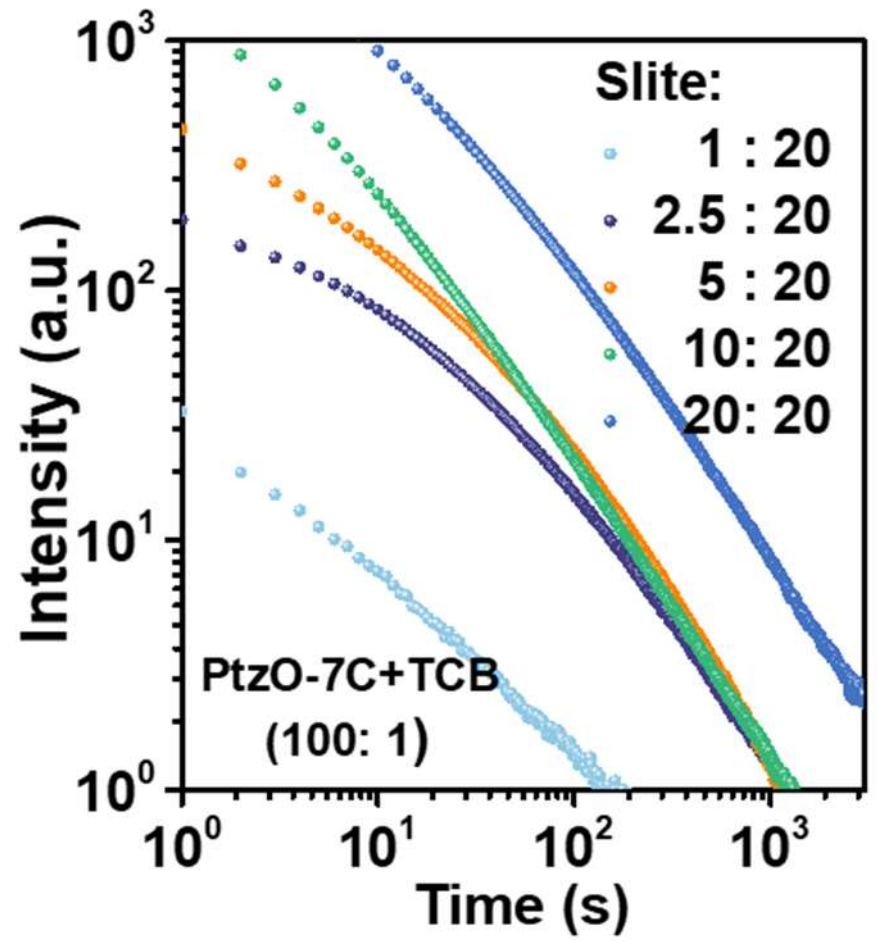

Fig. S10. Time-resolved OLPL-decay curves of 2PtzO-7C+TCB (100:1) excited through different slits at room temperature under atmosphere.

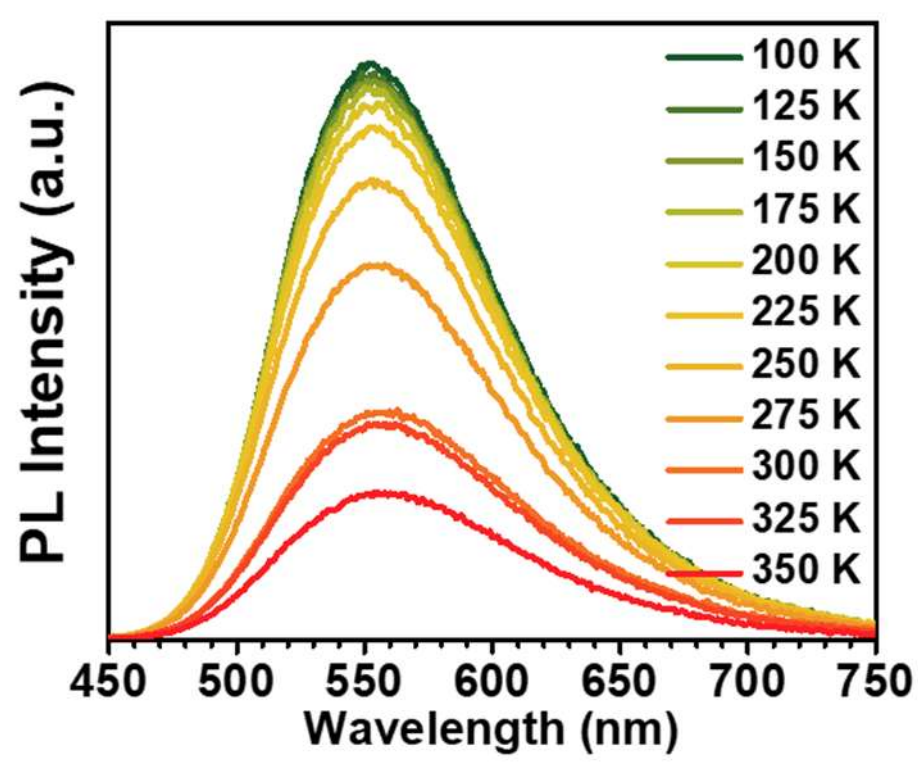

Fig. S11. Temperature-dependent PL spectra of 2PtzO-7C+TCB (100:1).

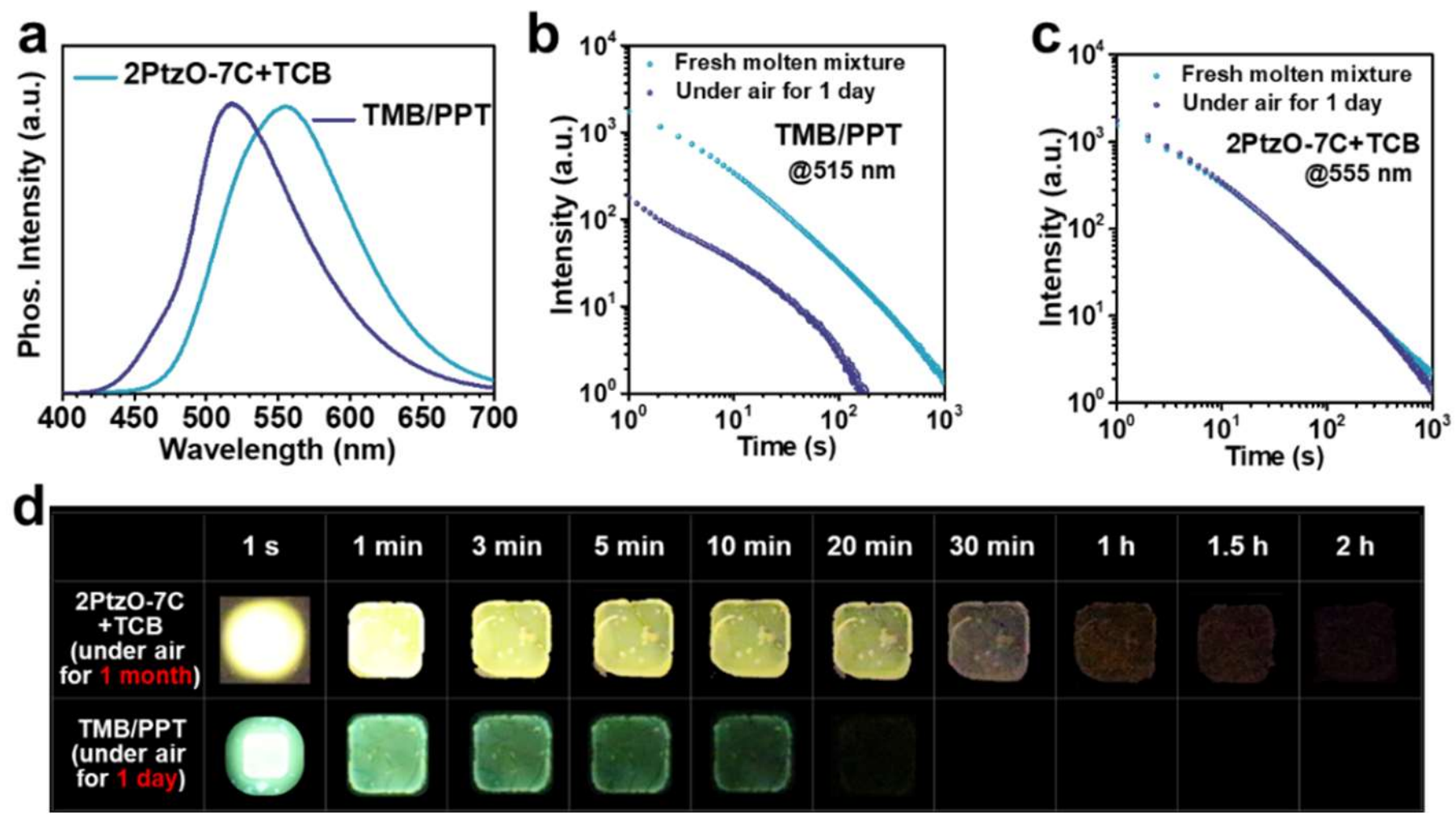

Fig. S12. a) OLPL spectra of 2PtzO-7C+TCB (100:1) and TMB/PPT. b) Time-resolved OLPL-decay curves of TMB/PPT at 515 nm in fresh state and after being stored at air for 1 day. c) Time-resolved OLPL-decay curves of 2PtzO-7C+TCB at 555 nm in fresh state and after being stored under air for 1 day. d) Photographs of 2PtzO-7C+TCB and TMB/PPT taken at different states.

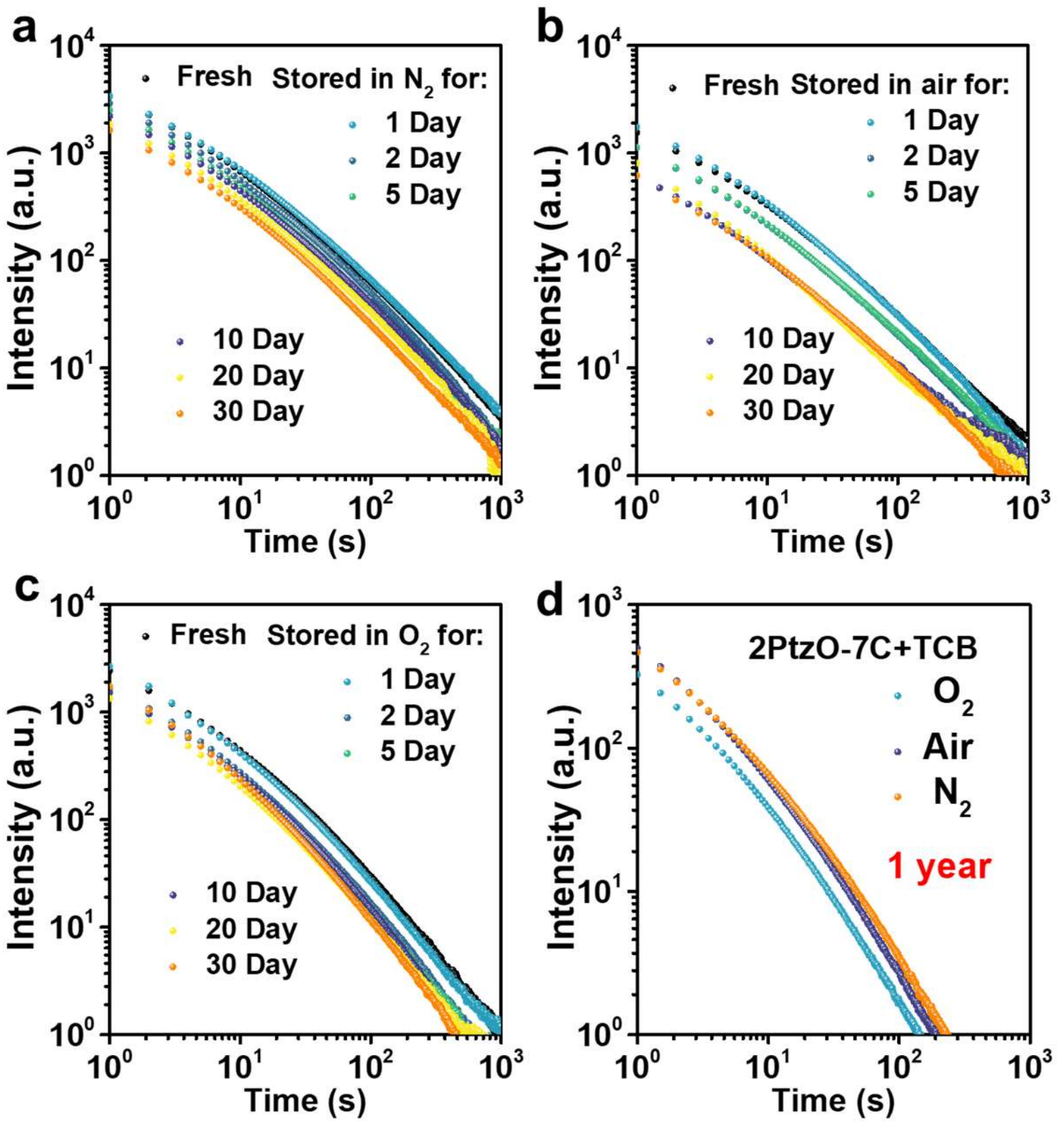

Fig. S13. a) Time-resolved OLPL-decay curves of 2PtzO-7C+TCB after being stored in nitrogen, b) in air and c) in oxygen for different times. d) Time-resolved OLPL-decay curves of 2PtzO-7C+TCB after being stored in different environment for 1 year.

Table S1. PLQY of 2PtzO-7C+TCB after being stored in different states for different times.

Storage time (day)		1	3	5	10	20	30
2PtzO-7C+TCB	$\Phi_{PL}{}^{a}$ (%)	22.79	22.67	21.27	20.74	19.56	20.16
	$\Phi_{PL}{}^{b}$ (%)	22.81	23.01	23.10	20.42	21.23	21.79
	$\Phi_{PL}{}^{c}$ (%)	22.06	22.74	21.74	22.22	22.03	20.86

[a] Under nitrogen; [b] Under air; [c] Under oxygen.

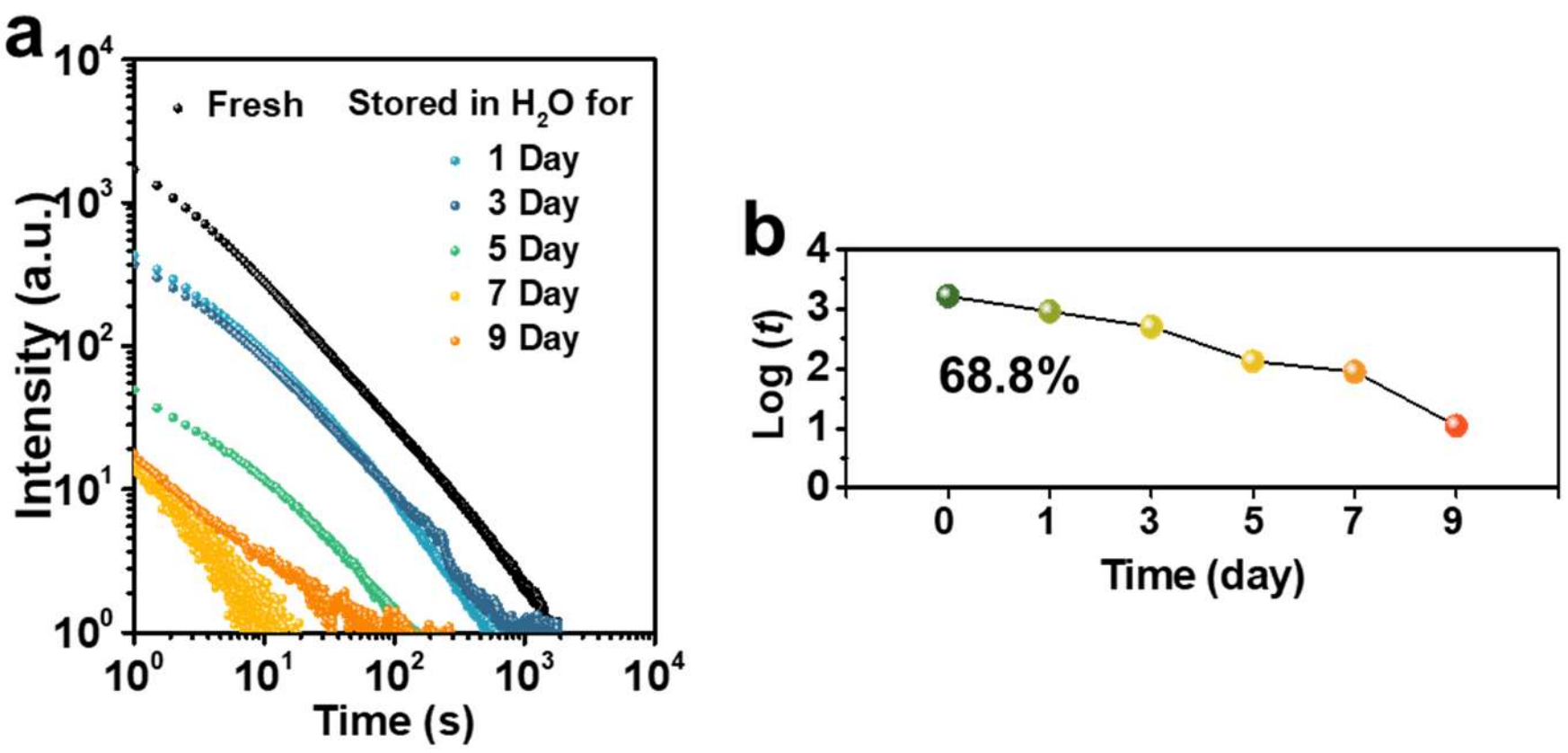

Fig. S14. a) Time-resolved OLPL-decay curves of 2PtzO-7C+TCB after being stored in water for different times. b) Variation of corresponding duration times.

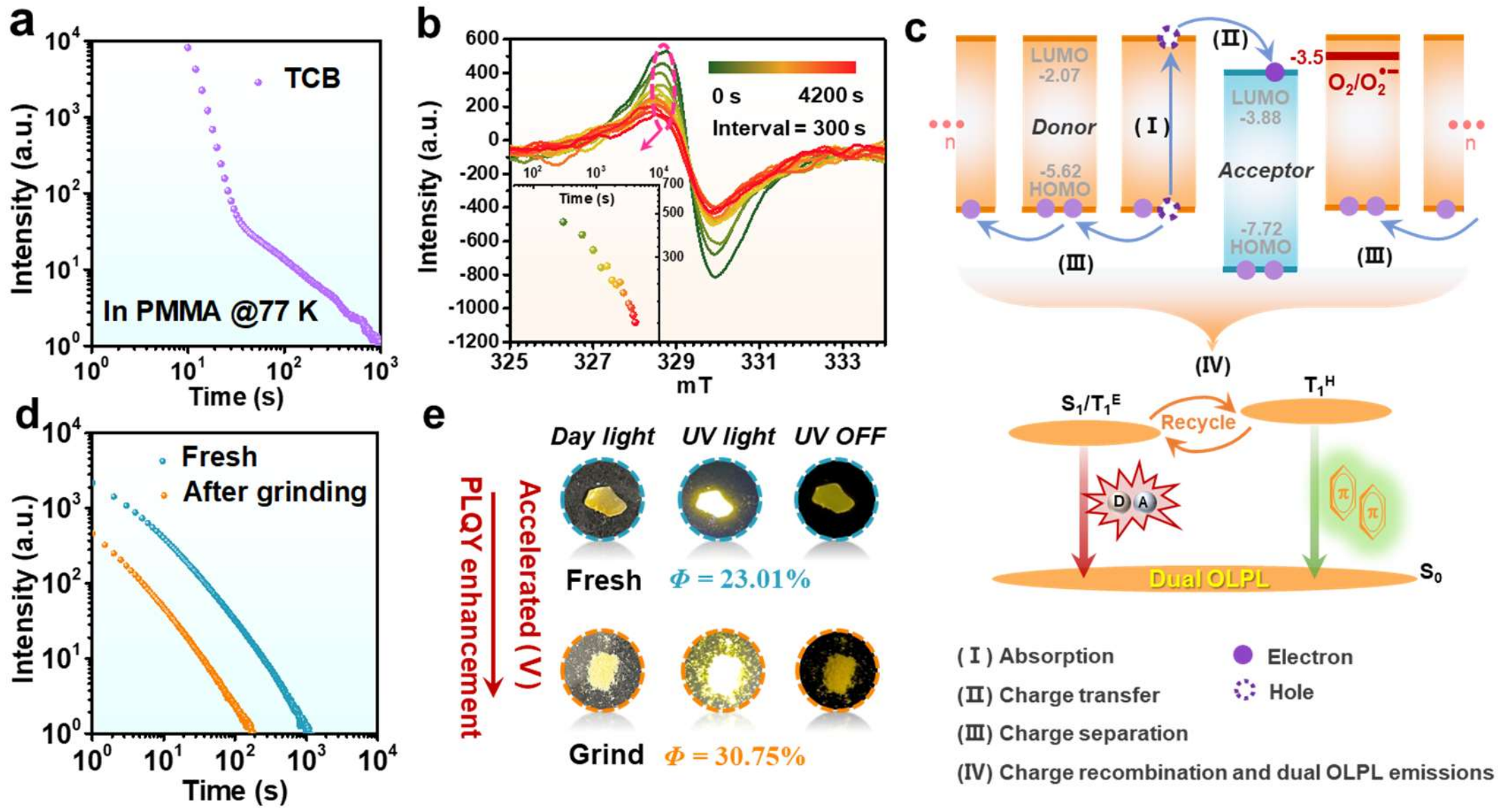

Fig. S15. (a) Time-resolved OLPL-decay curve of TCB doping in PMMA at 77 K. **(b)** Electron spin resonance (ESR) spectra of 2PtzO-7C+TCB under and after UV excitation for different times. Inset: corresponding time-resolved ESR intensity decay curve at 328.6 mT. **(c)** P-type dual OLPL emission mechanism: long-range charge diffusion on HOMO of donor host and the formed excitons through charge recombination could recycle between host (H) and exciplex (E) of donor-acceptor pair. **(d)** Time-resolved OLPL-decay curves of 2PtzO-7C+TCB before and after grinding at atmosphere. **(e)** Photographs of 2PtzO-7C+TCB taken under different light sources before and after grinding.

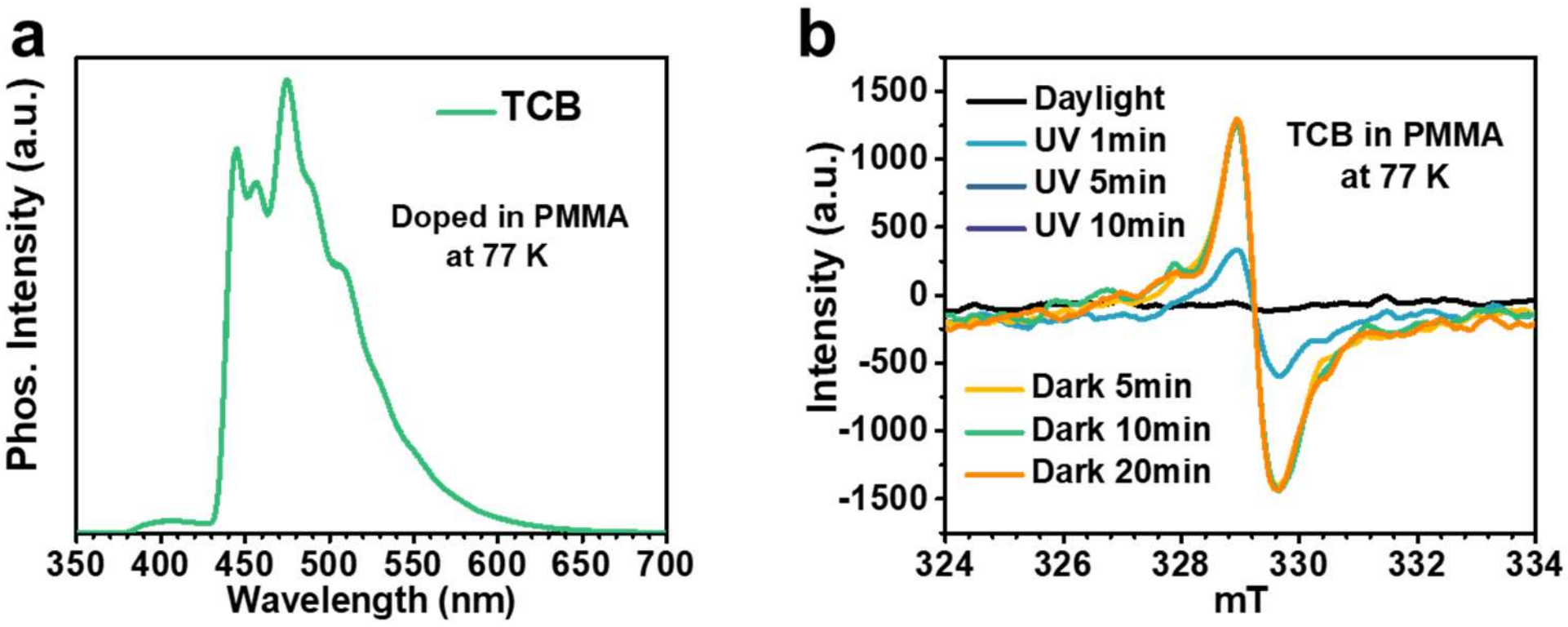

Fig. S16. a) Phosphorescence spectra of TCB doped in PMMA at 77 K (10^2: 1). b) Electron spin resonance (ESR) spectra of TCB doped in PMMA at 77 K before and after the removal of UV excitation.

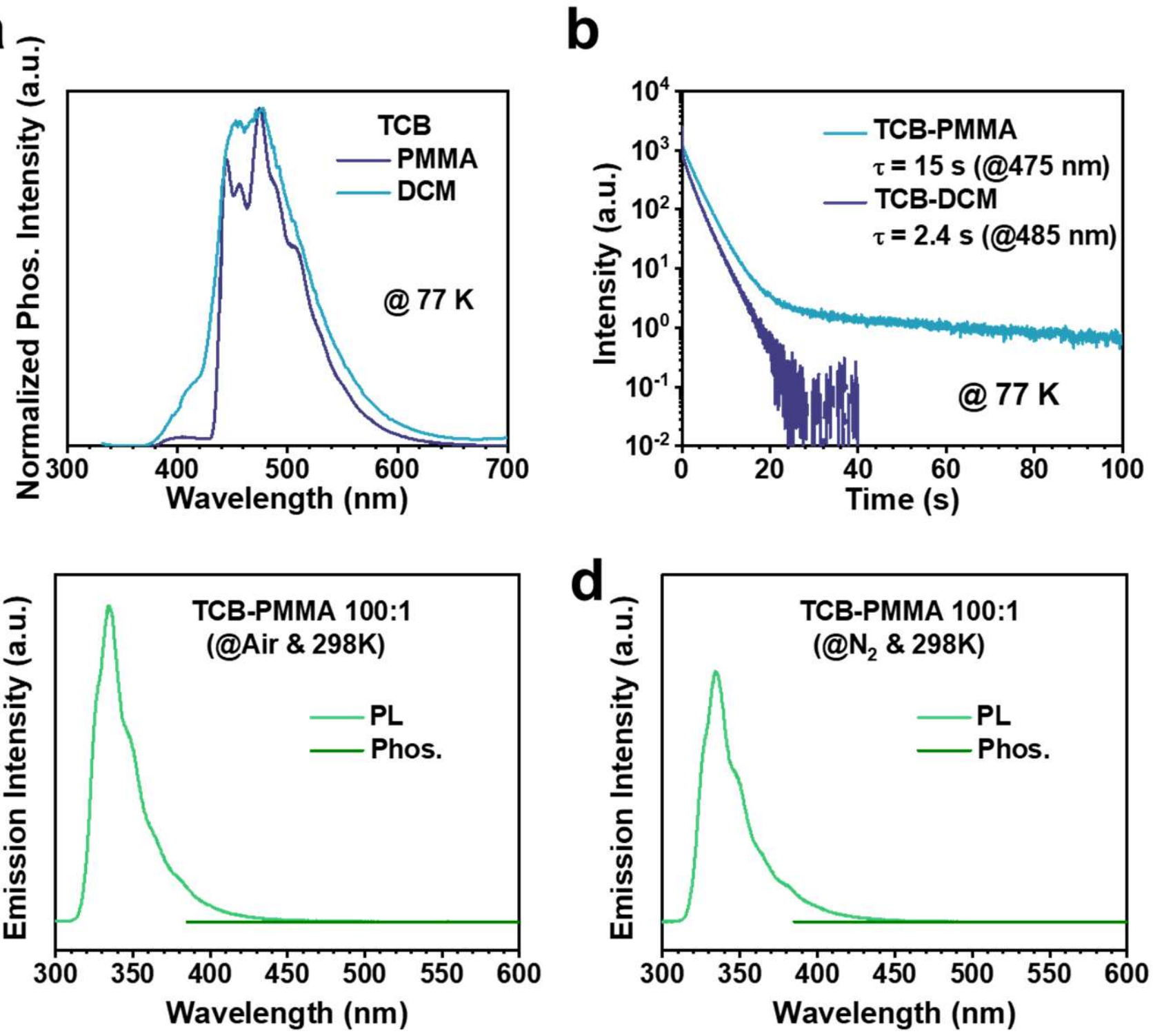

Fig. S17. a) Normalized phosphorescence spectra of TCB in PMMA matrix and DCM solution at 77 K. b) Time-resolved phosphorescence decay of TCB in PMMA film and DCM solution at 77 K, c) Steady-state PL and phosphorescence spectra of TCB in PMMA under air at room temperature. d) Steady-state PL and phosphorescence spectra of TCB in PMMA under N_2 at room temperature.

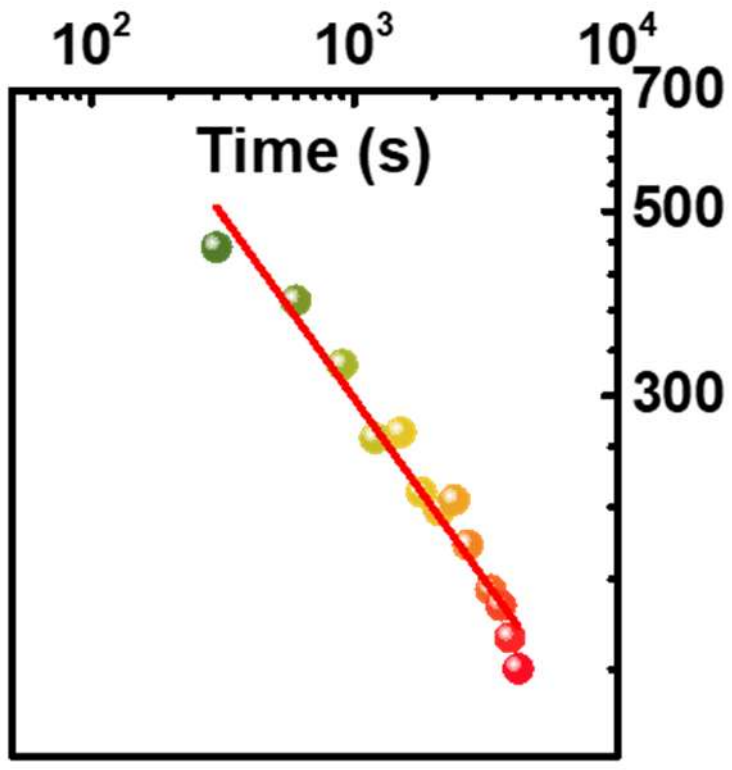

Fig. S18. Time-resolved ESR intensity decay curve of 2PtzO-7C+TCB at 328.6 mT.

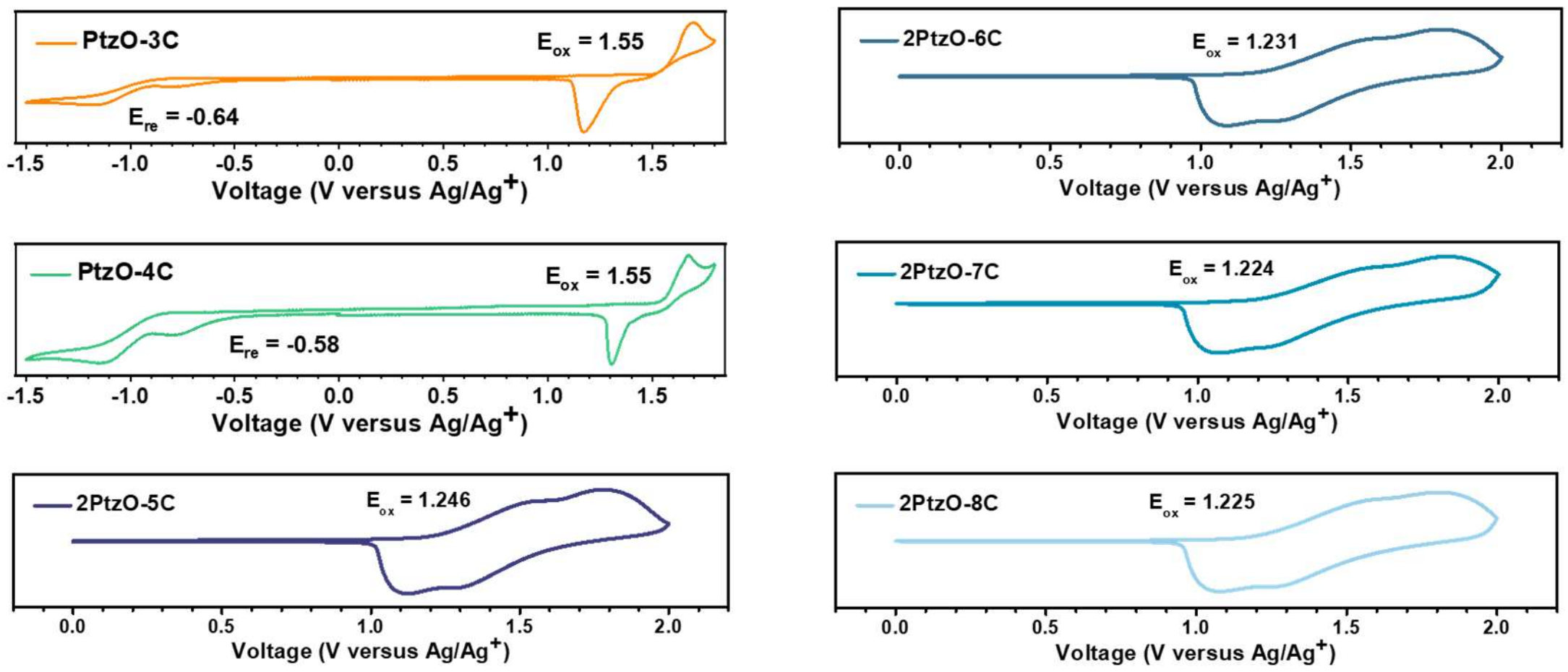

Fig. S19. CV curves of PtzO-3C, PtzO-4C, 2PtzO-5C, 2PtzO-6C, 2PtzO-7C and 2PtzO-8C.

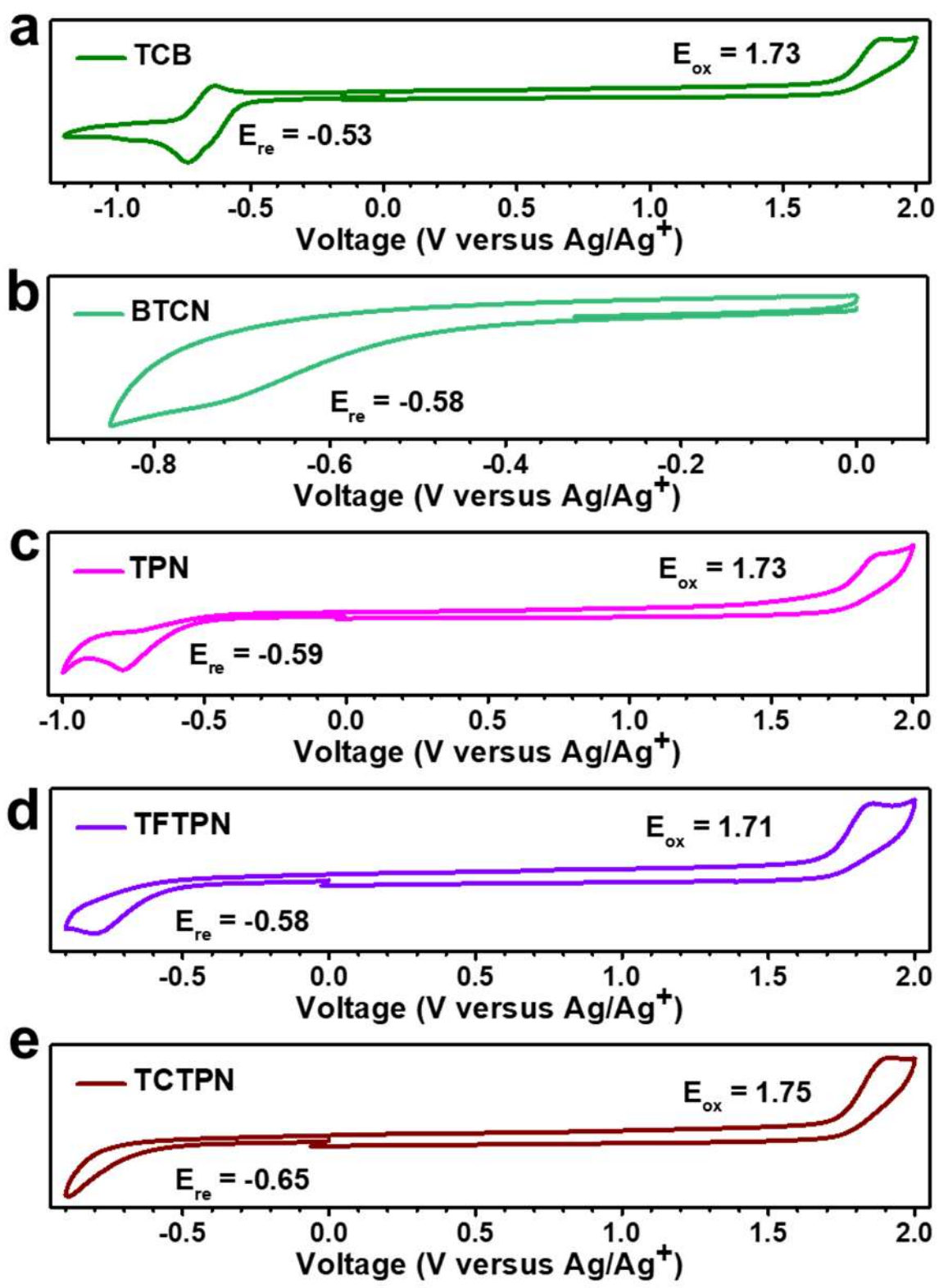

Fig. S20. a) CV curves of TCB, b) BTCN, c) TPN, d) TFTPN and e) TCTPN.

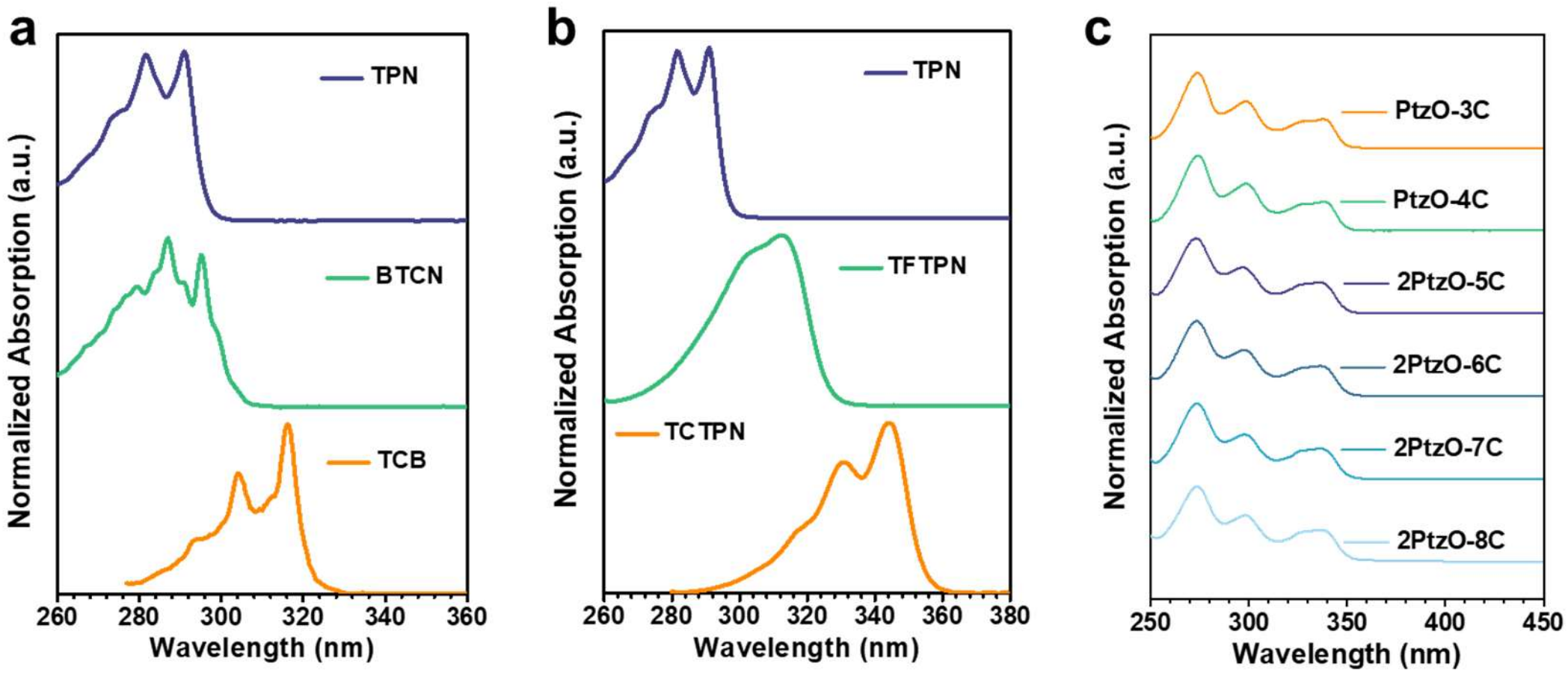

Fig. S21. a) UV-Vis absorption spectra of TPN, BTCN, TCB in DCM solution (10^{-5} M). b) UV-Vis absorption spectra of TPN, TFTPN, TCTPN in DCM solution (10^{-5} M). c) UV-Vis absorption spectra of PtzO-3C, PtzO-4C, 2PtzO-5C, 2PtzO-6C, 2PtzO-7C and 2PtzO-8C in DCM solution (10^{-5} M).

Table S2. Electrochemistry data of donor and acceptors.

	2PtzO-7C	TCB	TCTPN	TFTPN	TPN	BTCN
HOMO/eV	-5.62	-7.72	-7.25	-7.64	-8.00	-7.94
LUMO/eV	-2.07	-3.88	-3.76	-3.83	-3.82	-3.86
E_g /eV	3.55	3.84	3.49	3.81	4.18	4.08
E^{ox} /V	1.21		-	-	-	-
E^{red} /V	-	-0.53	-0.65	-0.58	-0.59	-0.55
λ_{onset}/nm	349.9	322.9	354.8	325.6	296.8	304.1
$E_{(Fc/Fc+)}$ /V	0.39					

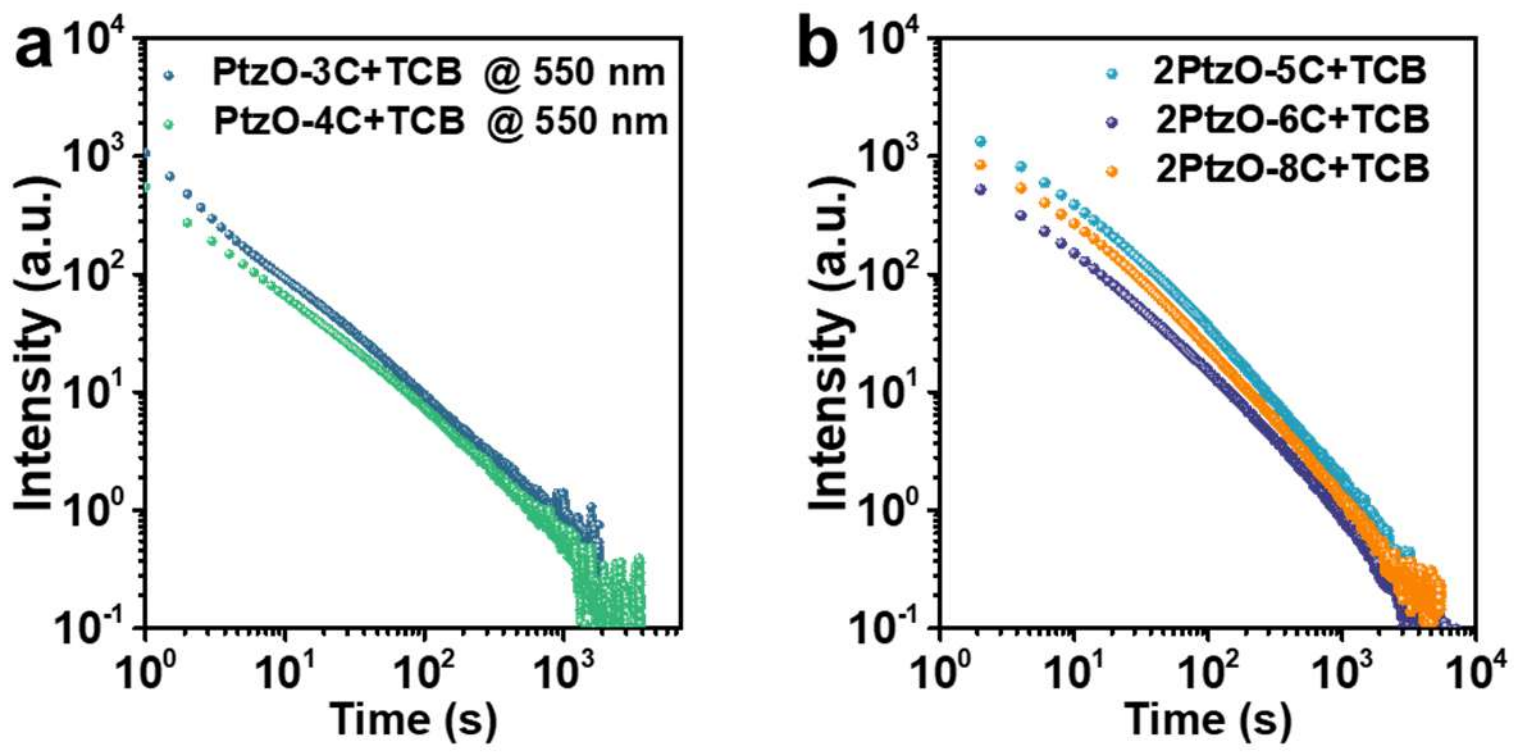

Fig. S22. a) Time-resolved OLPL-decay curves of PtzO-3C+TCB and PtzO-4C+TCB in air. b) Time-resolved OLPL-decay curves of 2PtzO-5C+TCB, 2PtzO-6C+TCB and 2PtzO-8C+TCB in air.

Table S3. OLPL data of six kinds of molten mixtures based on different hosts.

	PtzO-3C +TCB	PtzO-4C +TCB	2PtzO-5C +TCB	2PtzO-6C +TCB	2PtzO-7C +TCB	2PtzO-8C +TCB
Φ	20.74	21.21	14.58	16.54	23.01	16.03
t(s)	707.9	630.1	1412.5	1174.9	7154.3	1288.5

Φ = PLQY detected at room temperature;

t(s) = duration time detected at room temperature.

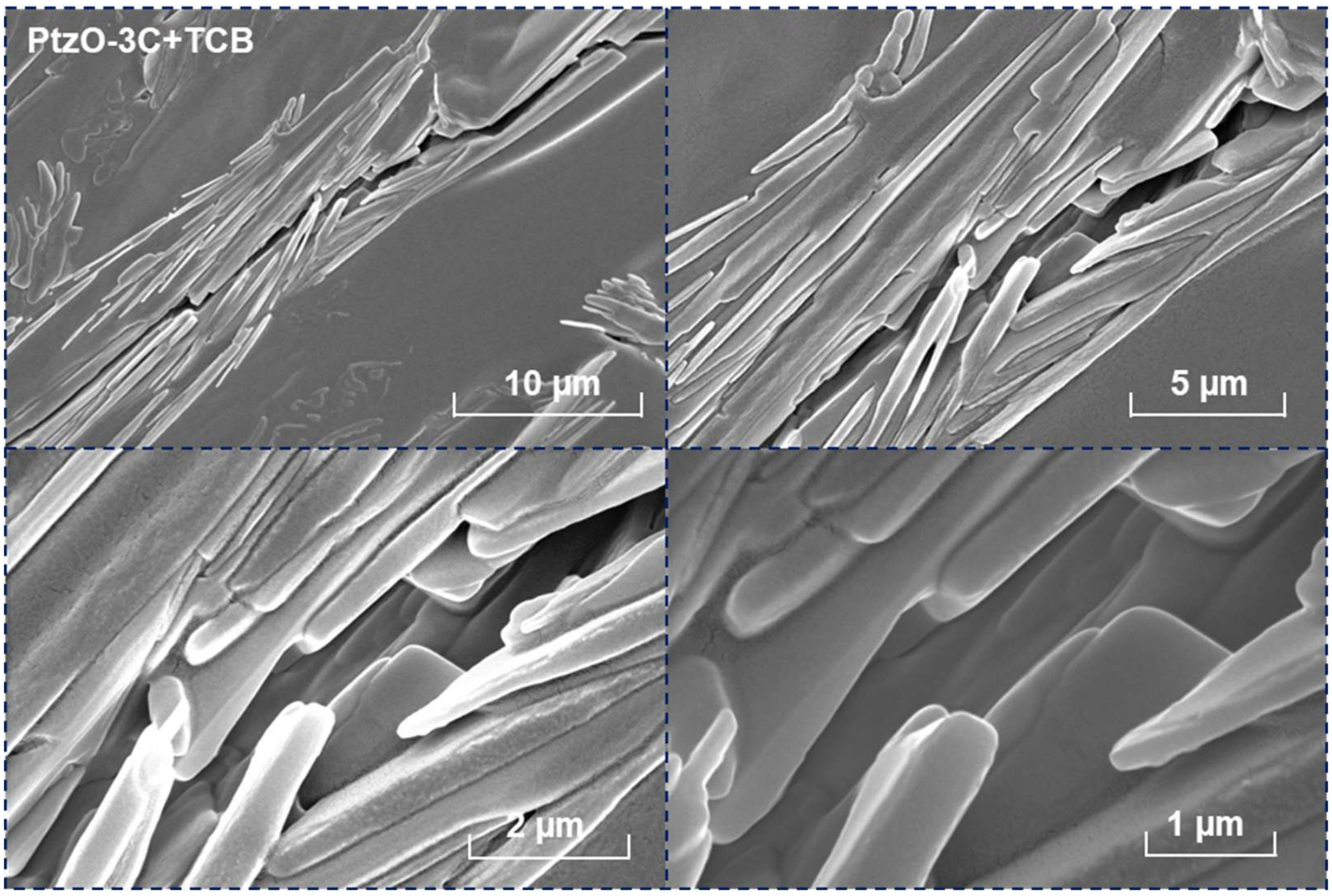

Fig. S23. Scanning electron microscope (SEM) diagram of PtzO-3C+TCB.

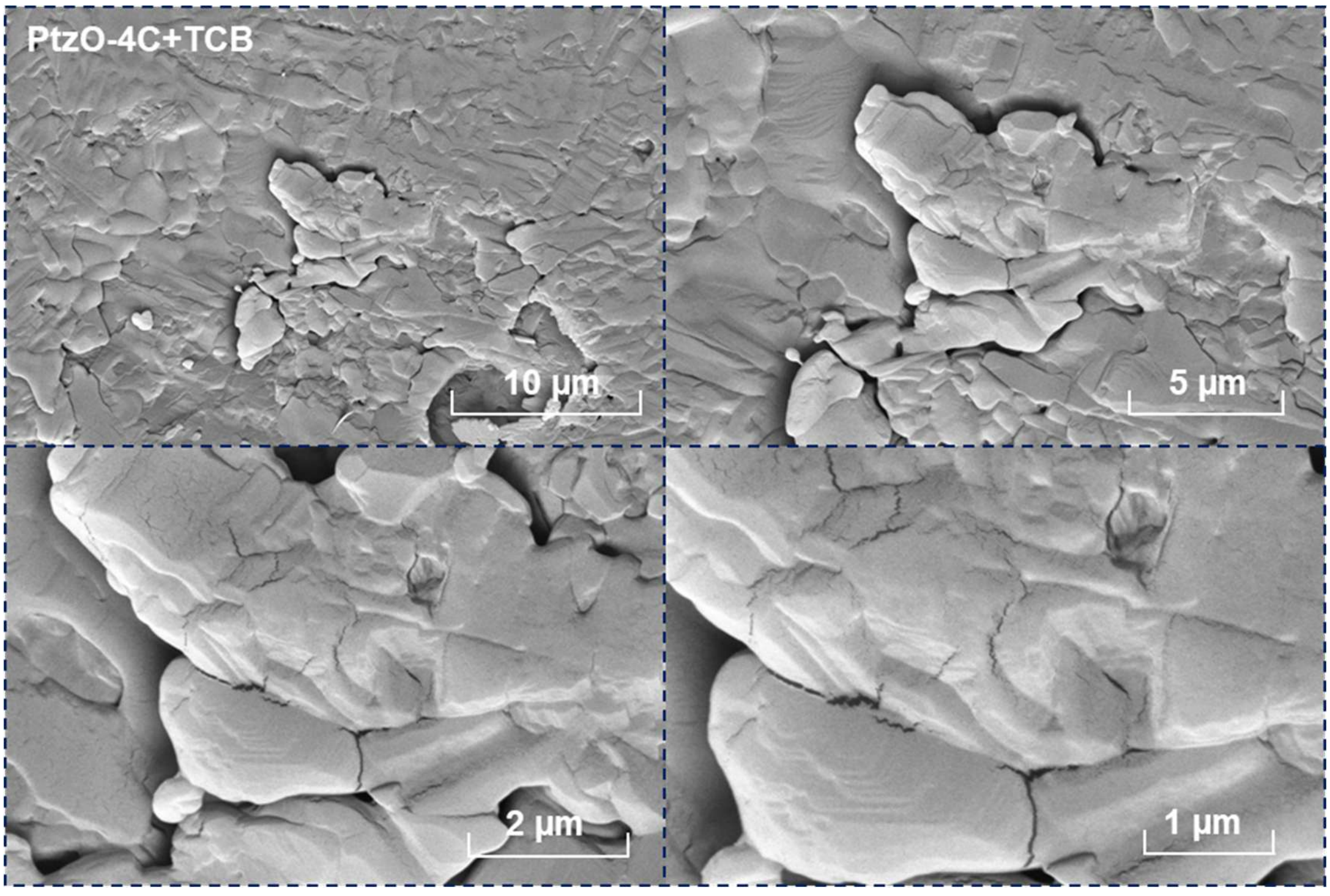

Fig. S24. Scanning electron microscope (SEM) diagram of PtzO-4C+TCB.

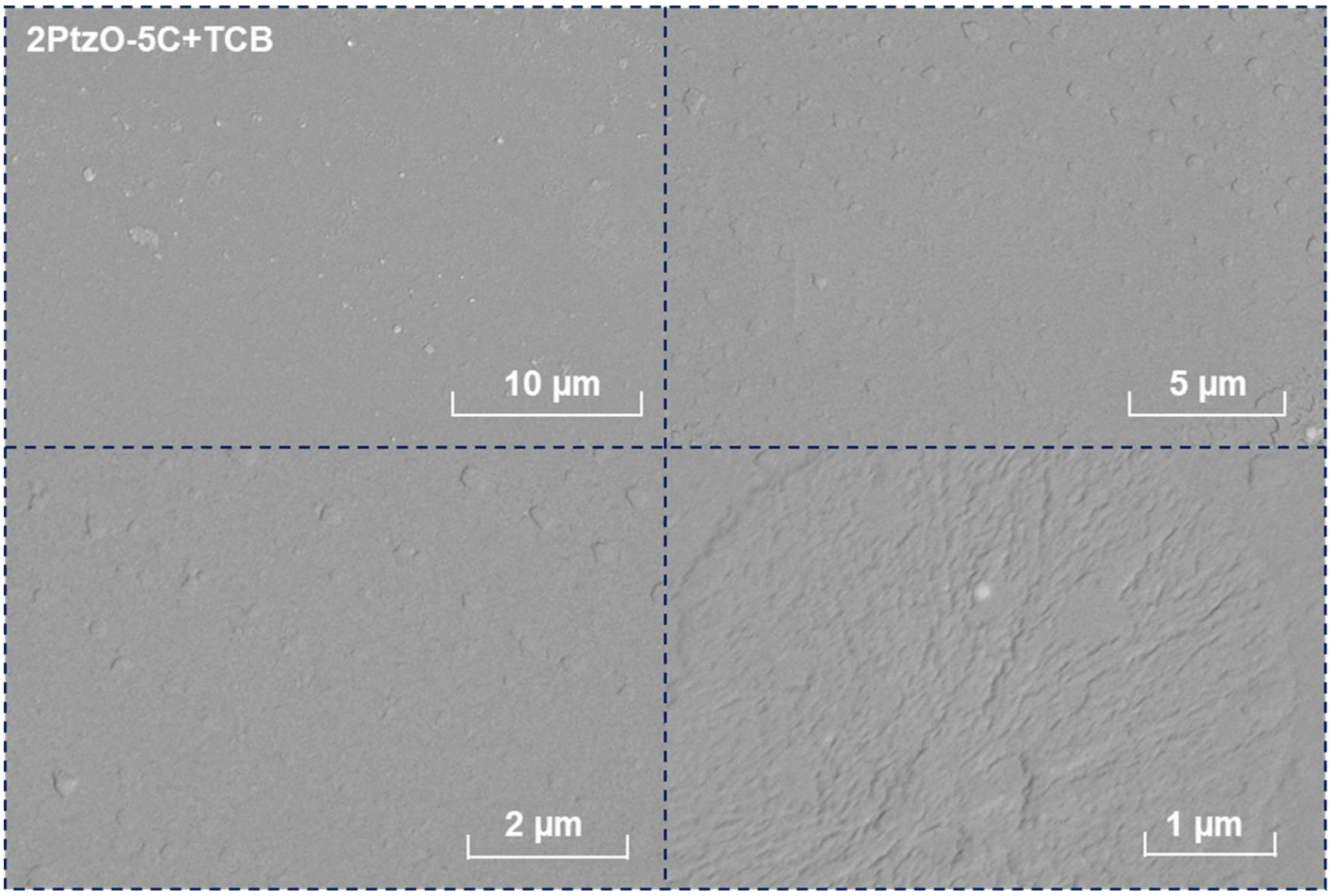

Fig. S25. Scanning electron microscope (SEM) diagram of 2PtzO-5C+TCB.

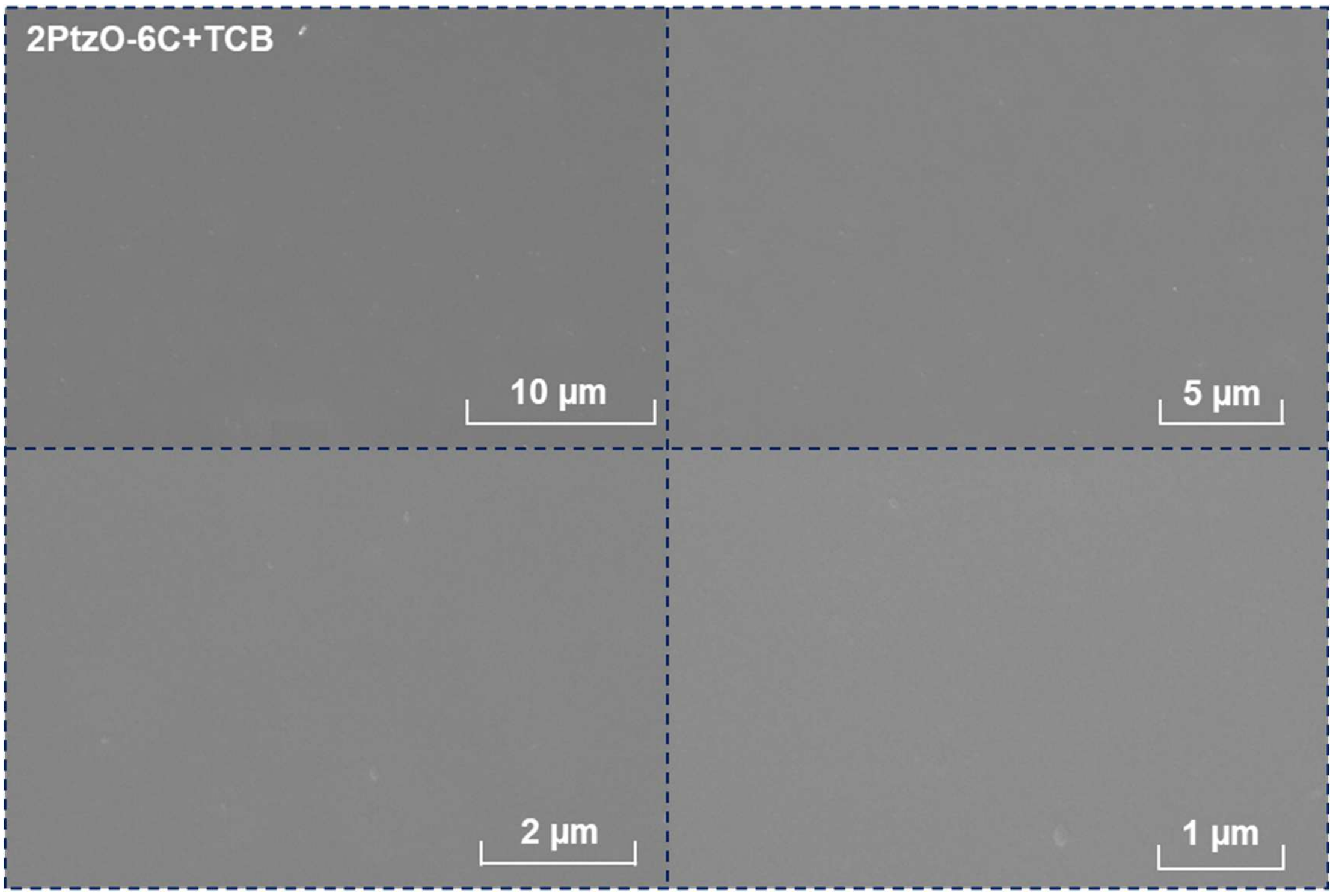

Fig. S26. Scanning electron microscope (SEM) diagram of 2PtzO-6C+TCB.

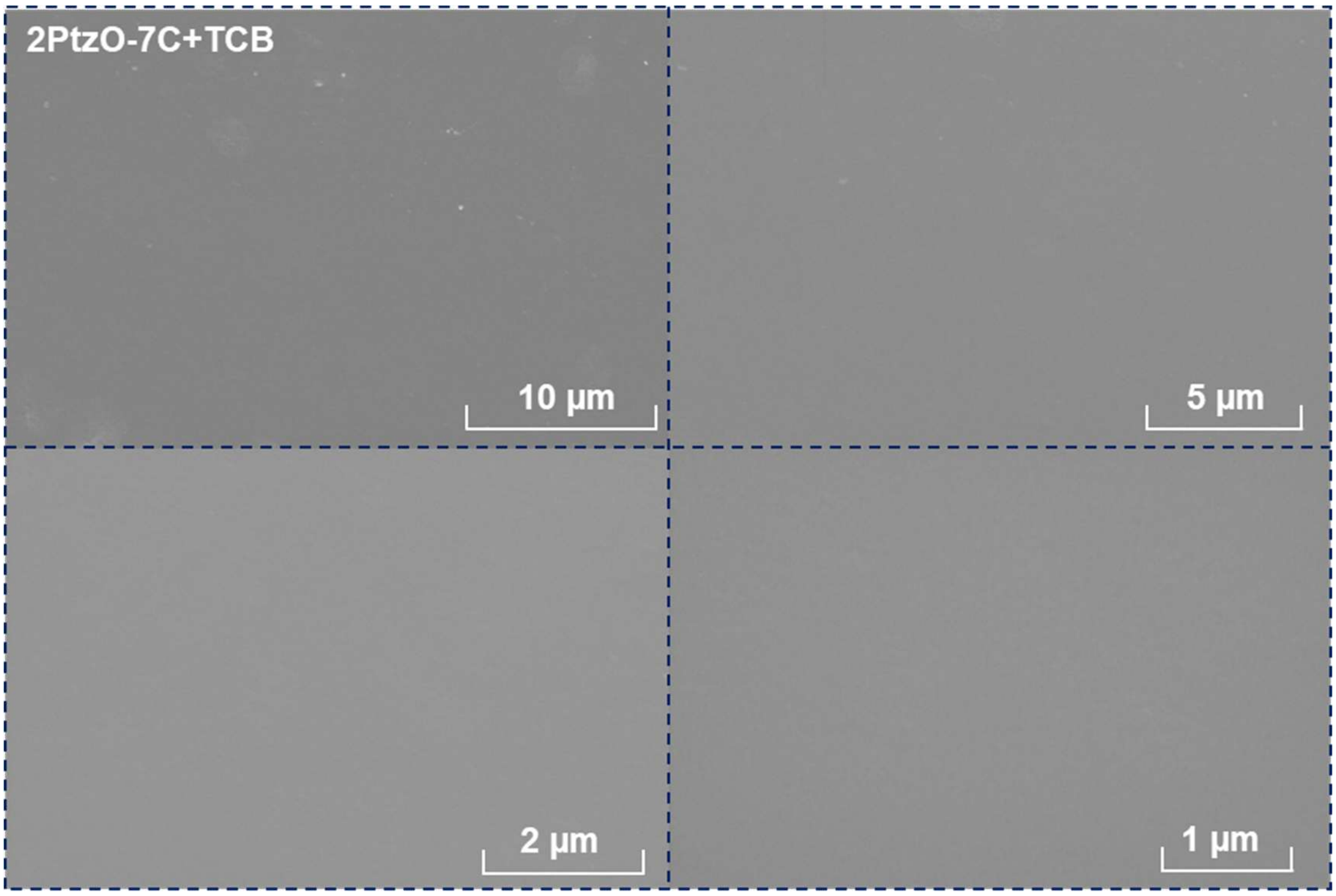

Fig. S27. Scanning electron microscope (SEM) diagram of 2PtzO-7C+TCB.

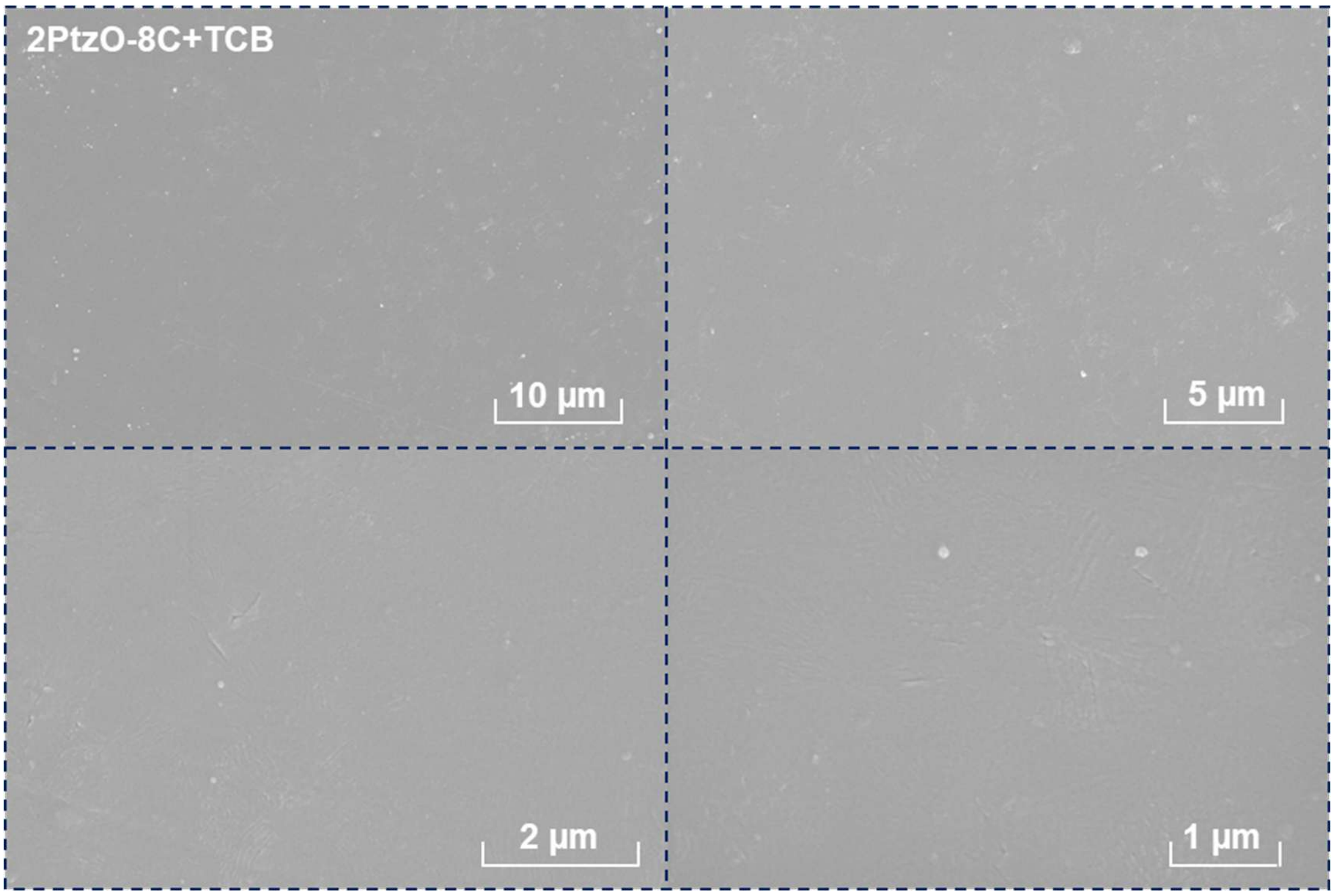

Fig. S28. Scanning electron microscope (SEM) diagram of 2PtzO-8C+TCB.

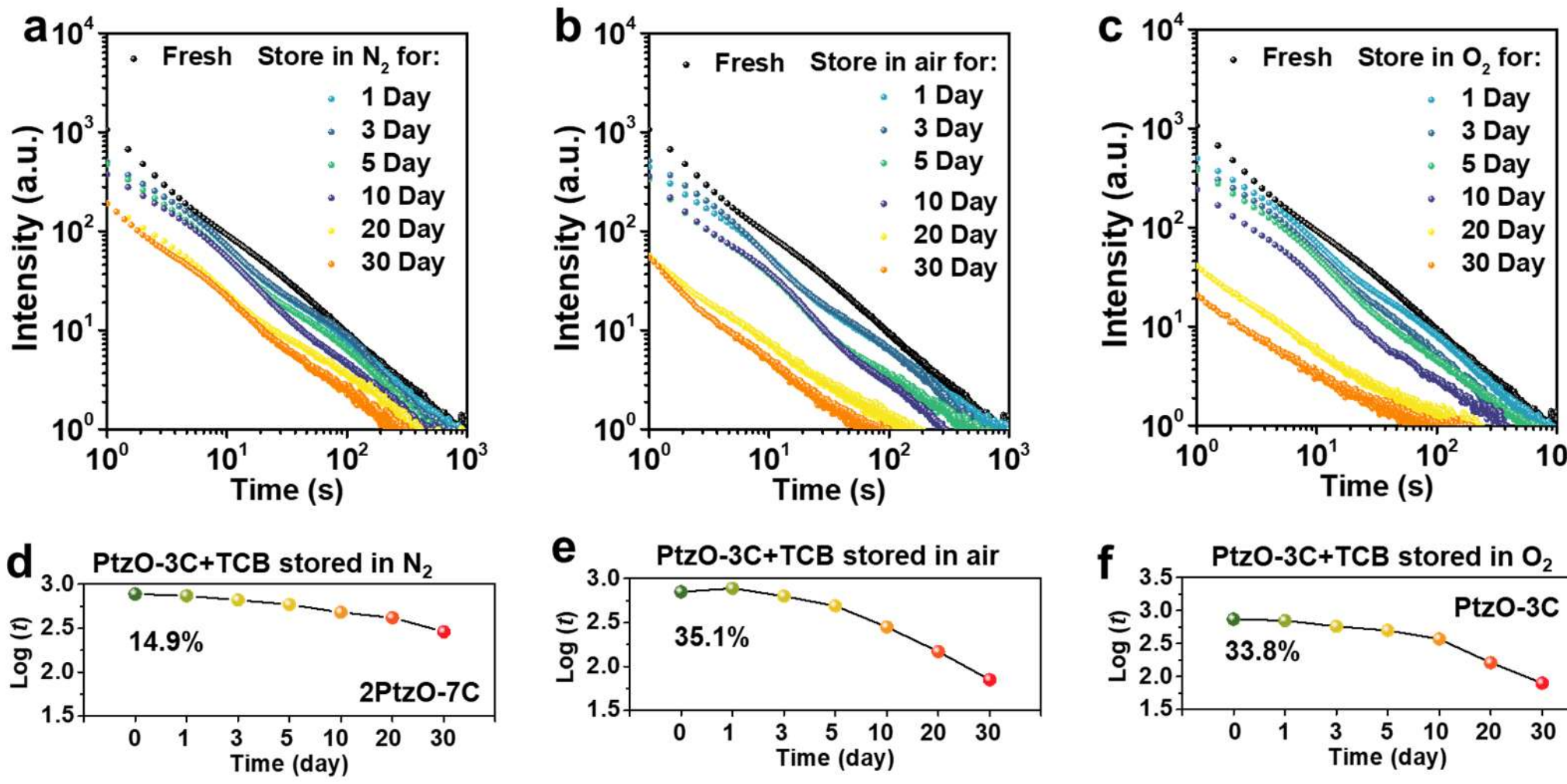

Fig. S29. a) Time-resolved OLPL-decay curves of PtzO-3C+TCB after being stored in nitrogen, b) in air and c) in oxygen for different times. d) Variation of OLPL duration times of PtzO-3C+TCB stored in nitrogen, e) in air and c) in oxygen for different times.

Table S4. PLQY of PtzO-3C+TCB after being stored in different states for different times.

Storage time (day)		1	3	5	10	20	30
PtzO-3C +TCB	Φ_{PL}^{a} (%)	21.60	22.53	23.13	21.03	22.76	22.06
	Φ_{PL}^{b} (%)	22.85	21.62	22.65	20.00	22.12	21.62
	Φ_{PL}^{c} (%)	19.62	20.24	22.09	19.31	20.03	19.41

[a] Under nitrogen; [b] Under air; [c] Under oxygen.

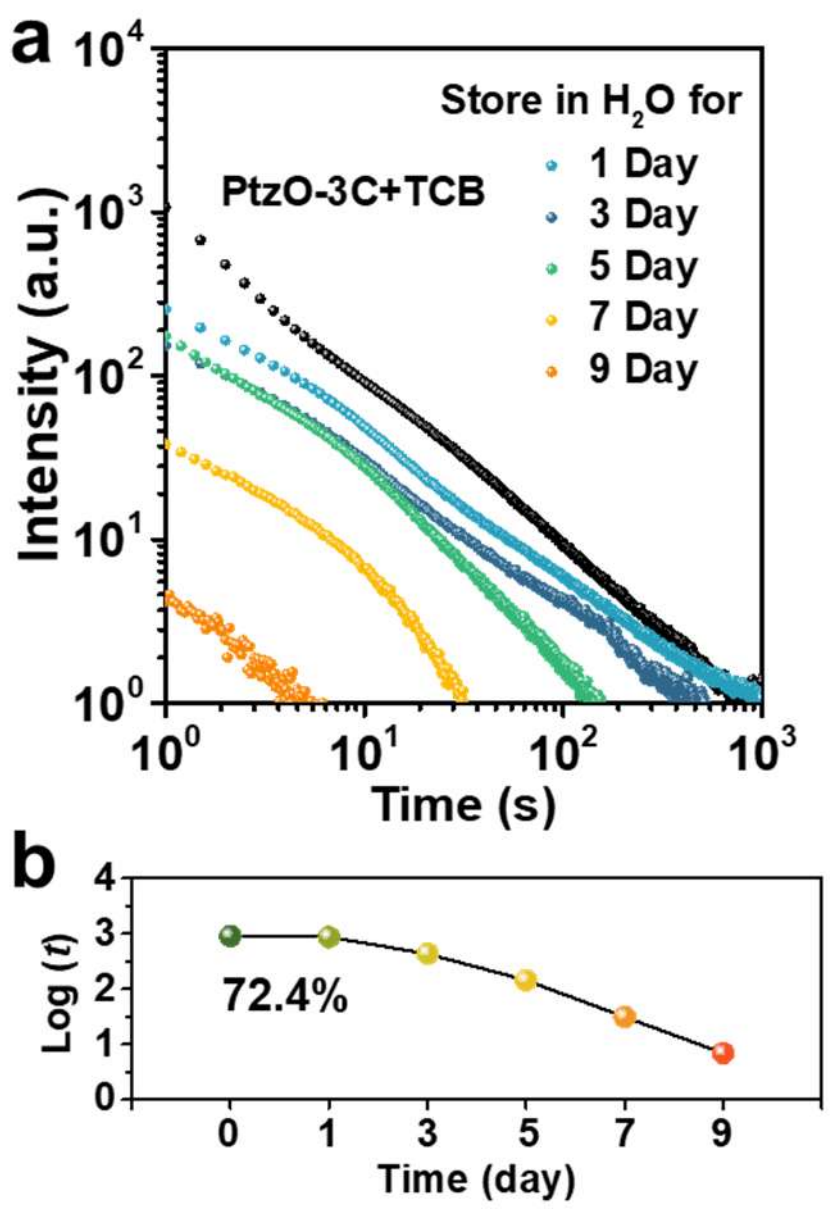

Fig. S30. a) Time-resolved OLPL-decay curves of PtzO-3C+TCB after being stored in water for different times. b) Variation of corresponding duration times.

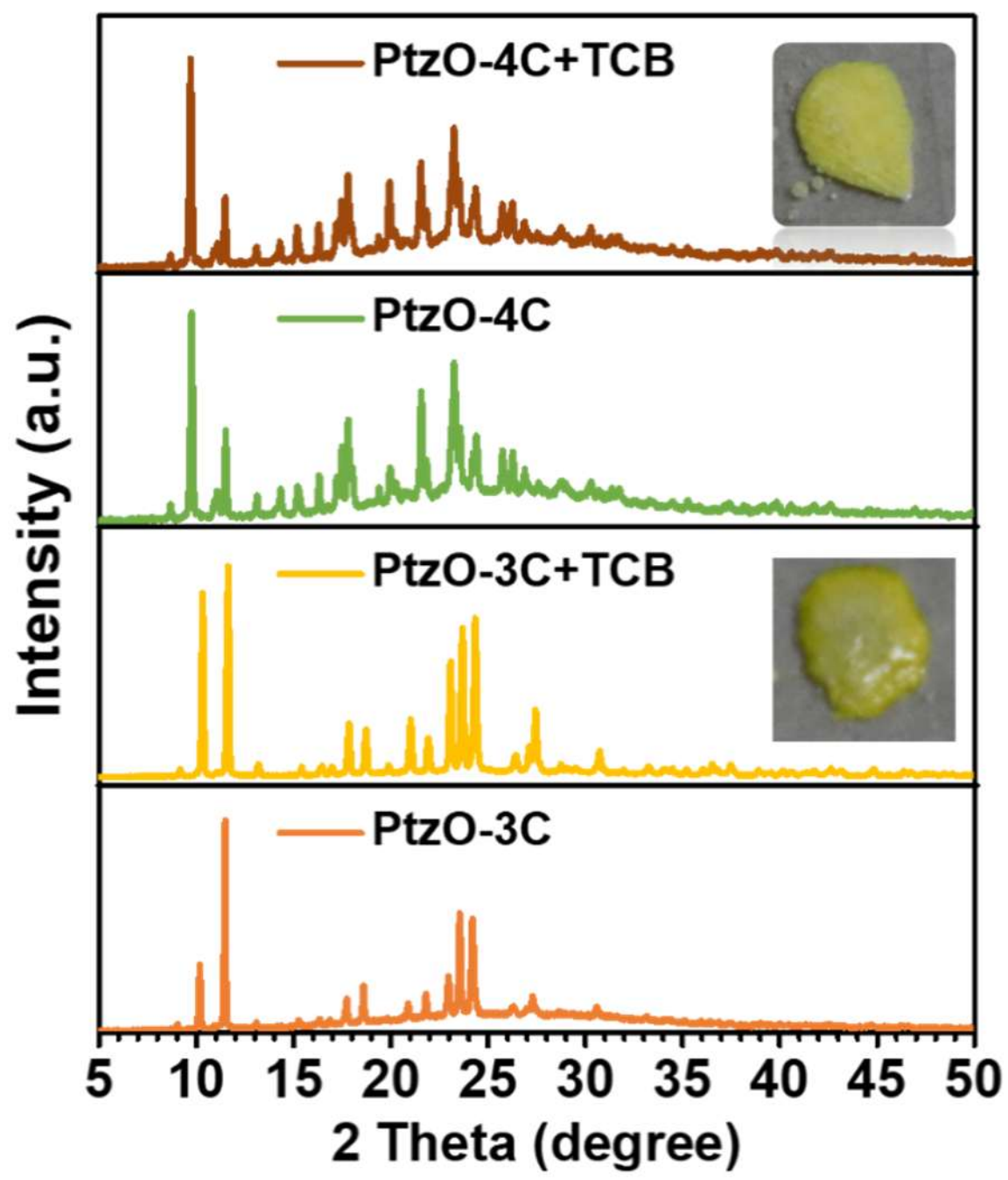

Fig. S31. PXRD patterns of PtzO-3C+TCB, PtzO-4C+TCB and corresponding host crystals.

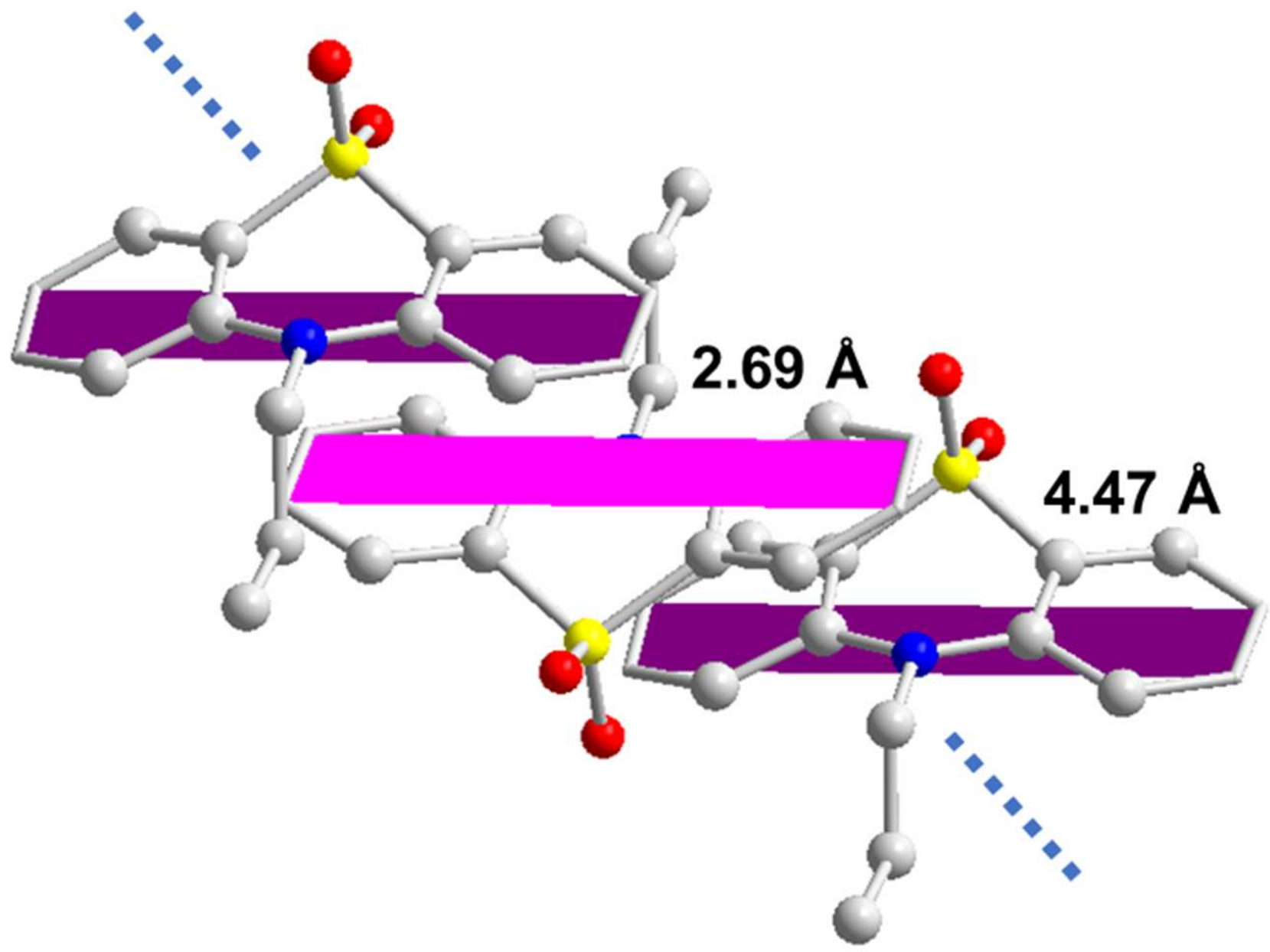

Fig. S32. Local packing modes of PtzO-3C.

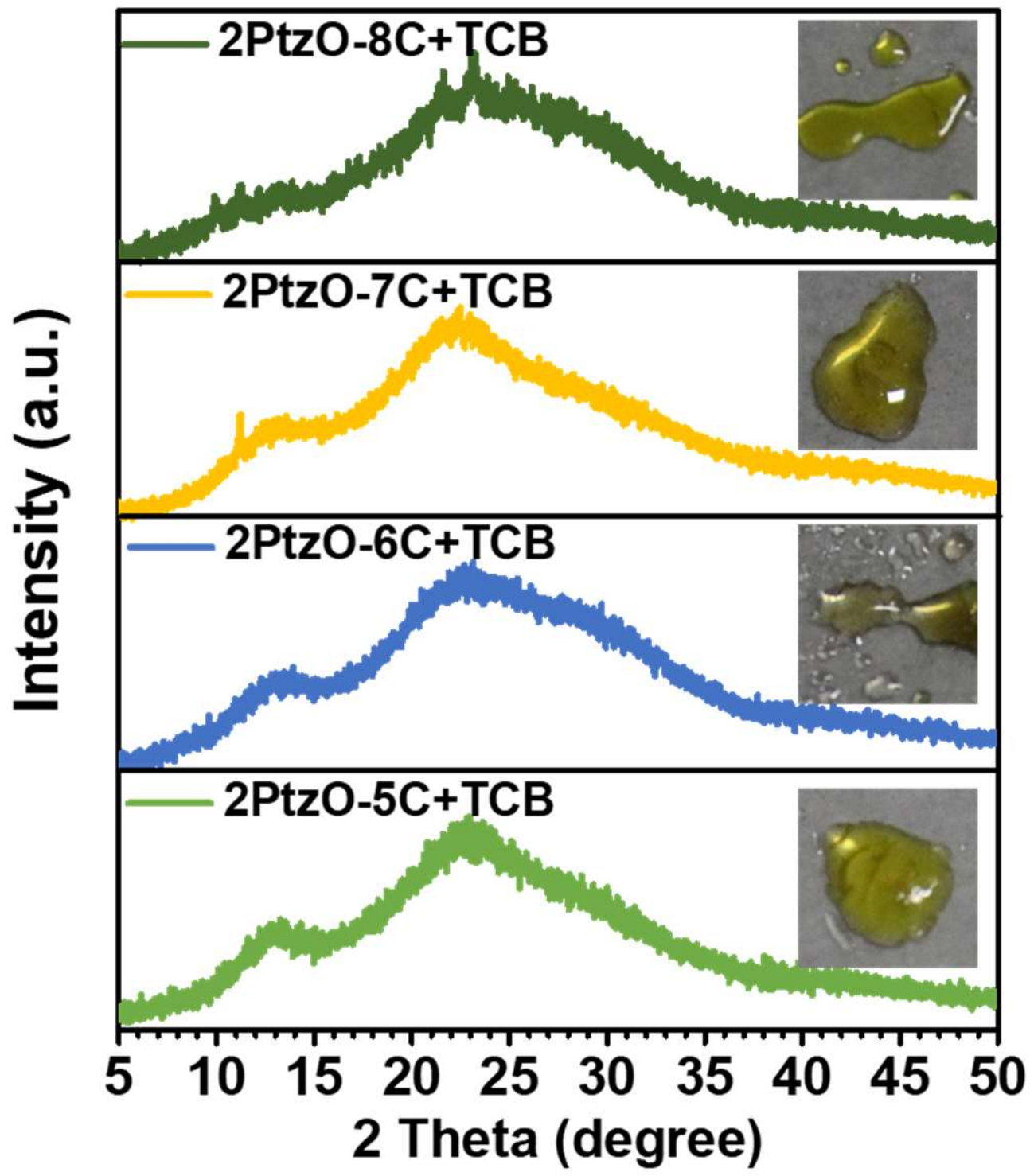

Fig. S33. PXRD patterns of 2PtzO-nC+TCB (n = 5-8).

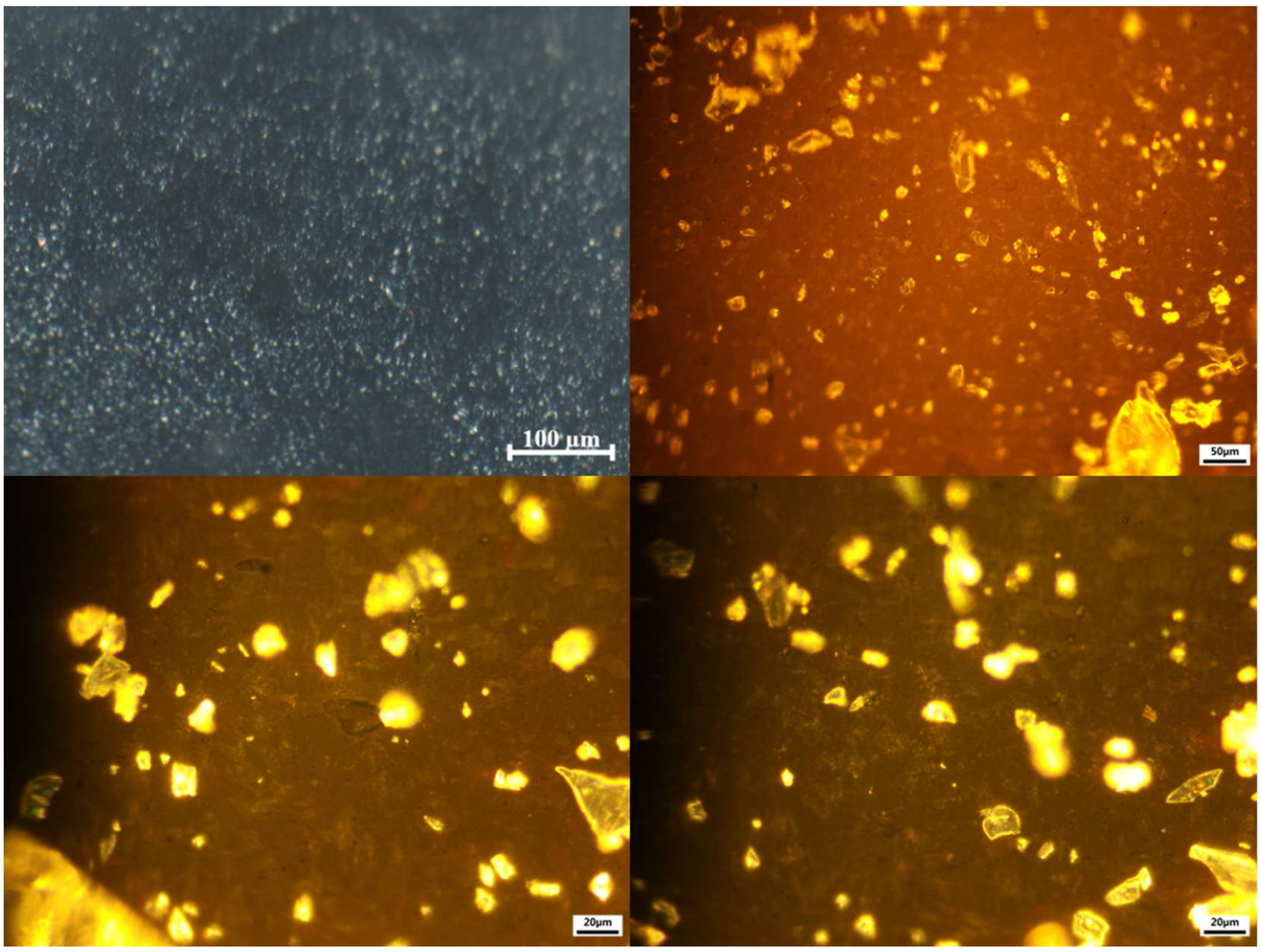

Fig. S34. POM images of 2PtzO-7C+TCB at room temperature.

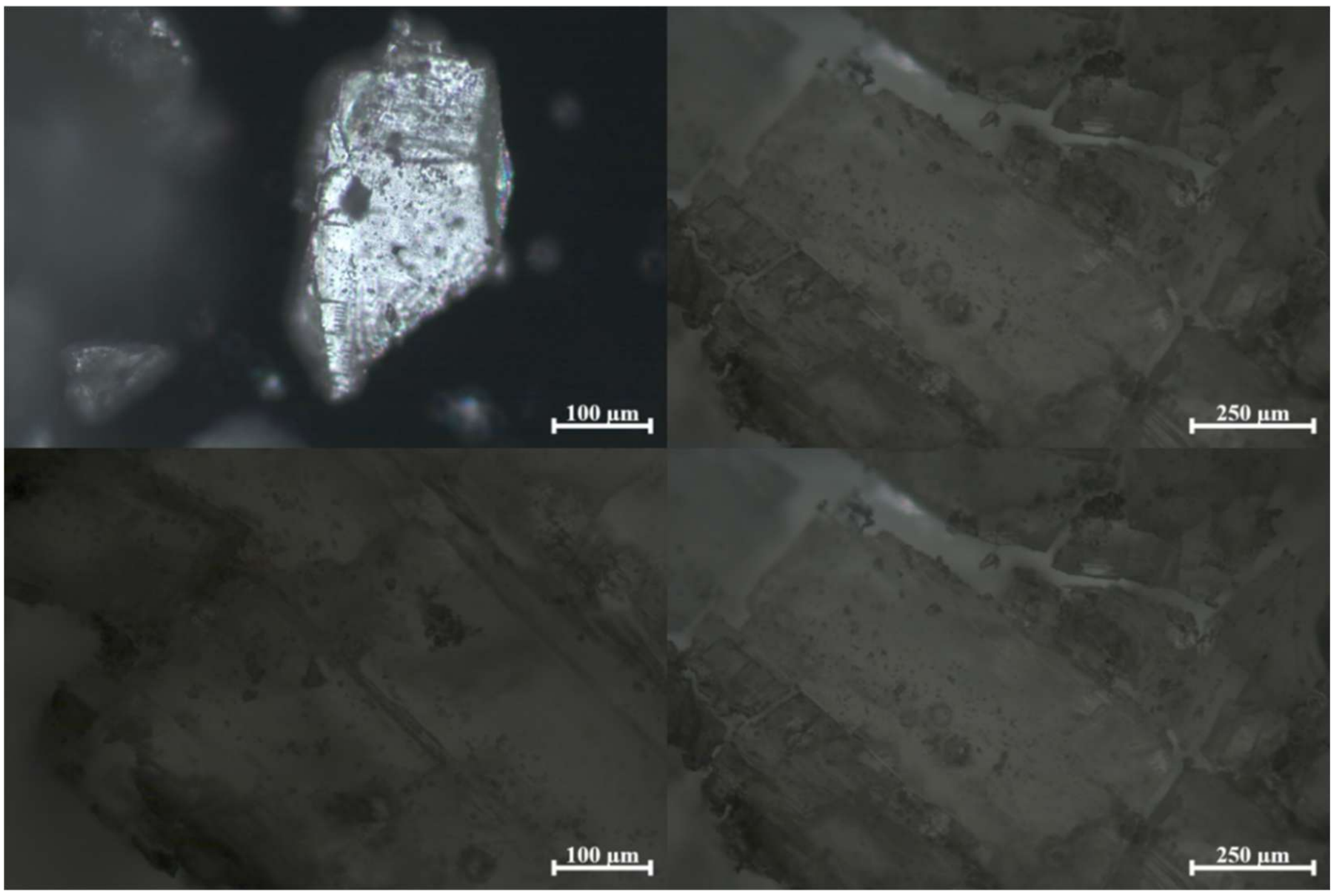

Fig. S35. POM images of PtzO-3C+TCB at room temperature.

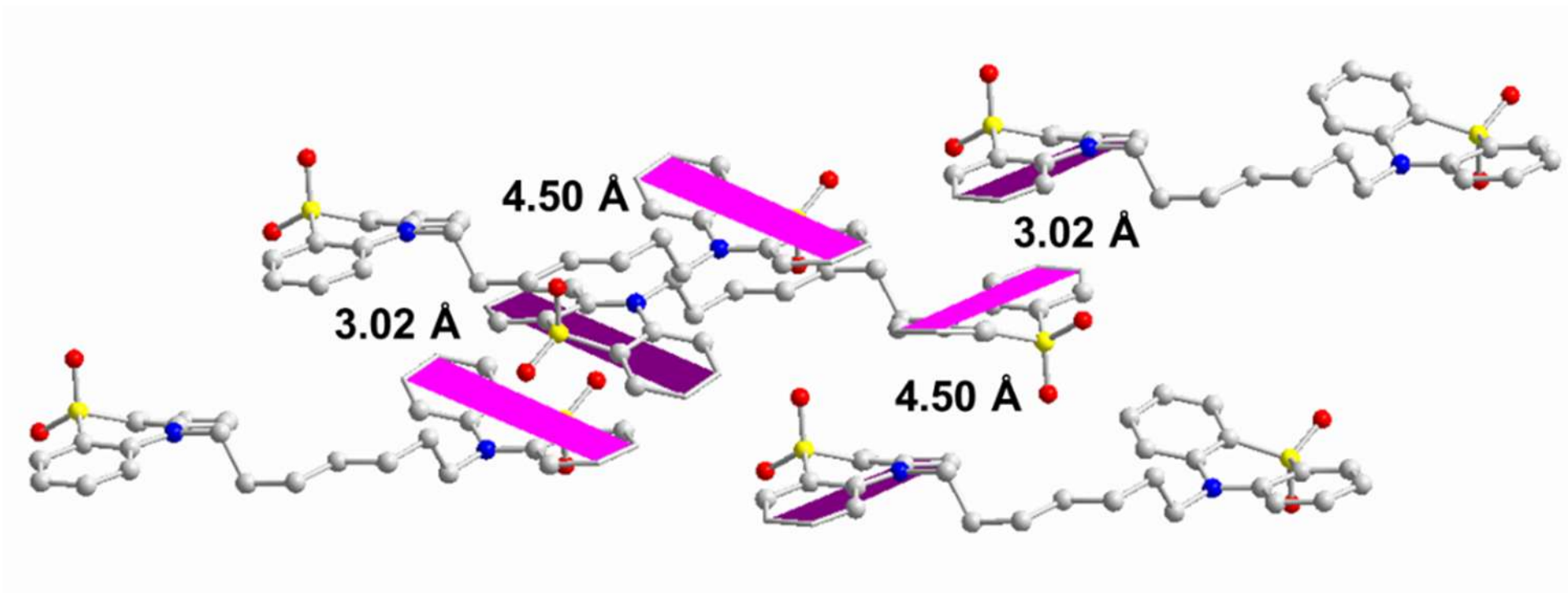

Fig. S36. Local packing modes of 2PtzO-7C.

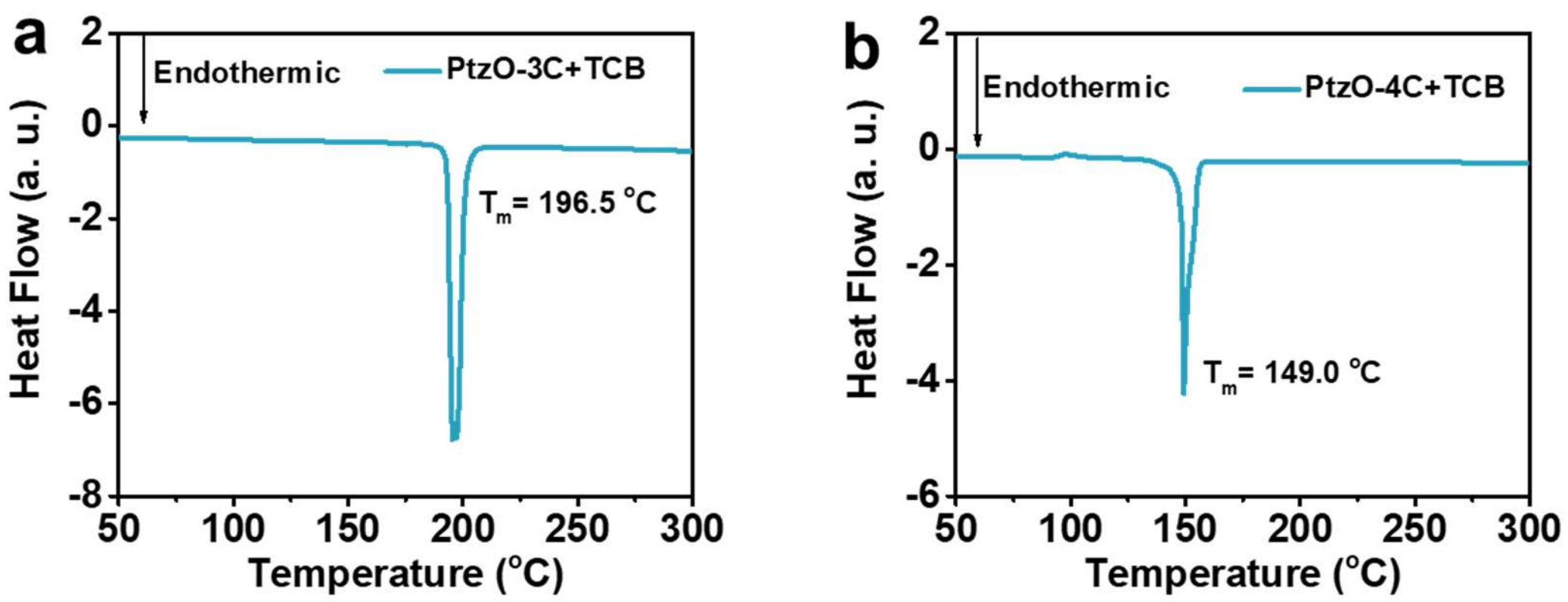

Fig. S37. a) DSC curves of PtzO-3C+TCB and b) PtzO-4C+TCB.

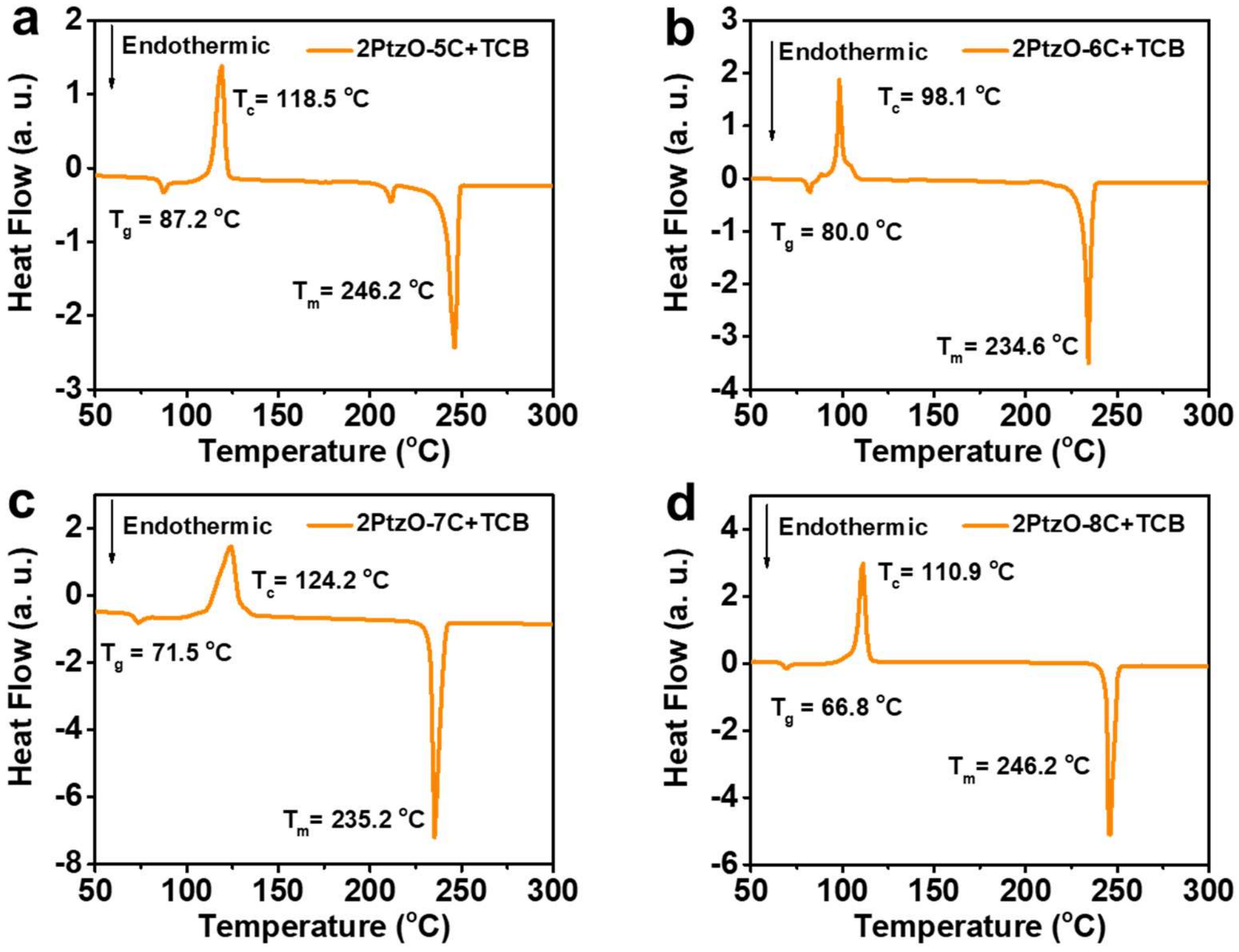

Fig. S38. a) DSC curves of 2PtzO-5C+TCB, b) 2PtzO-6C+TCB, c) 2PtzO-7C+TCB and d) 2PtzO-8C+TCB.

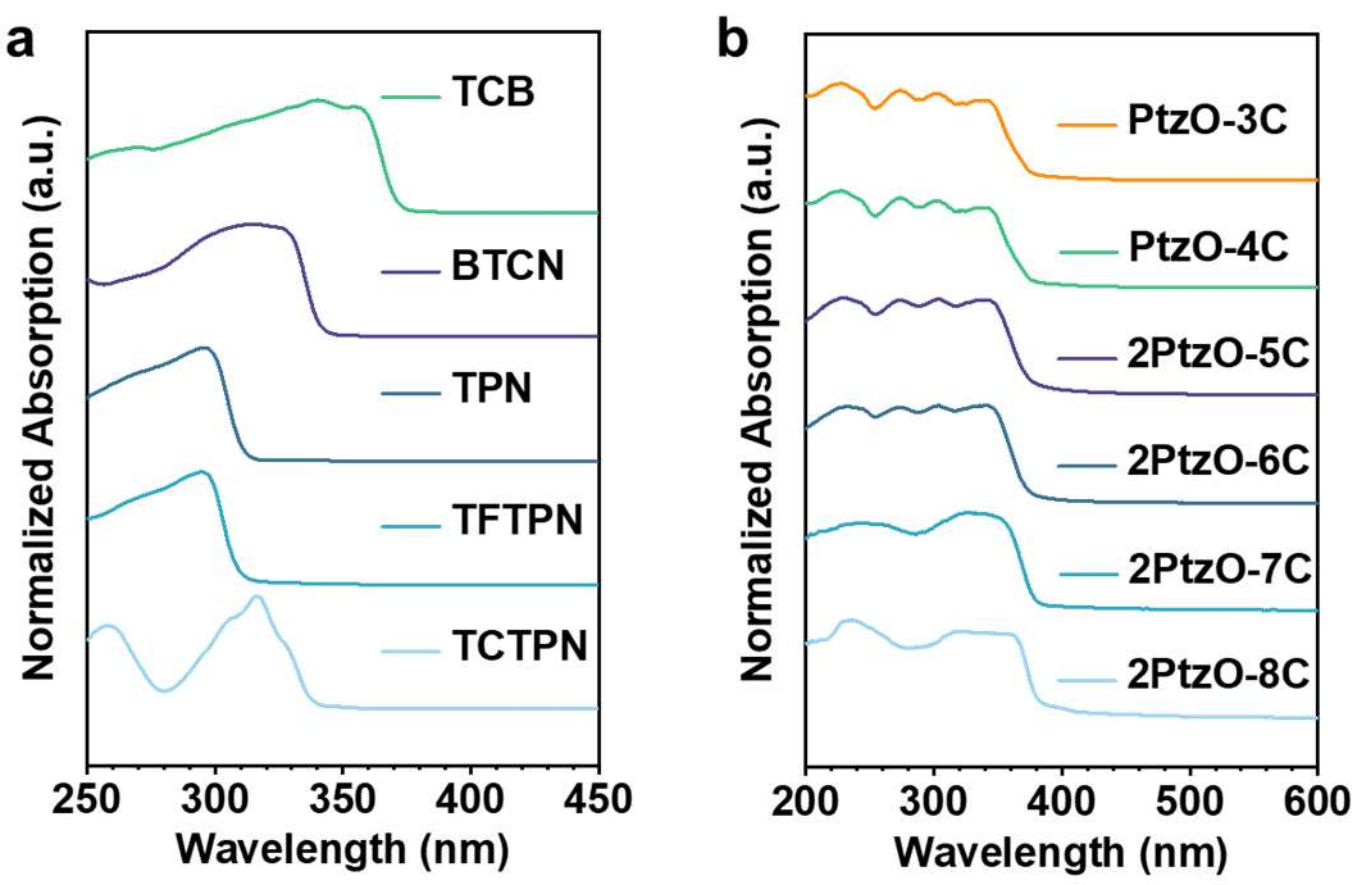

Fig. S39. a) UV-Vis absorption spectra of TCB, BTCN, TPN, TFTPN and TCTPN solids. b) UV-Vis absorption spectra of PtzO-3C, PtzO-4C, 2PtzO-5C, 2PtzO-6C, 2PtzO-7C, 2PtzO-8C solids.

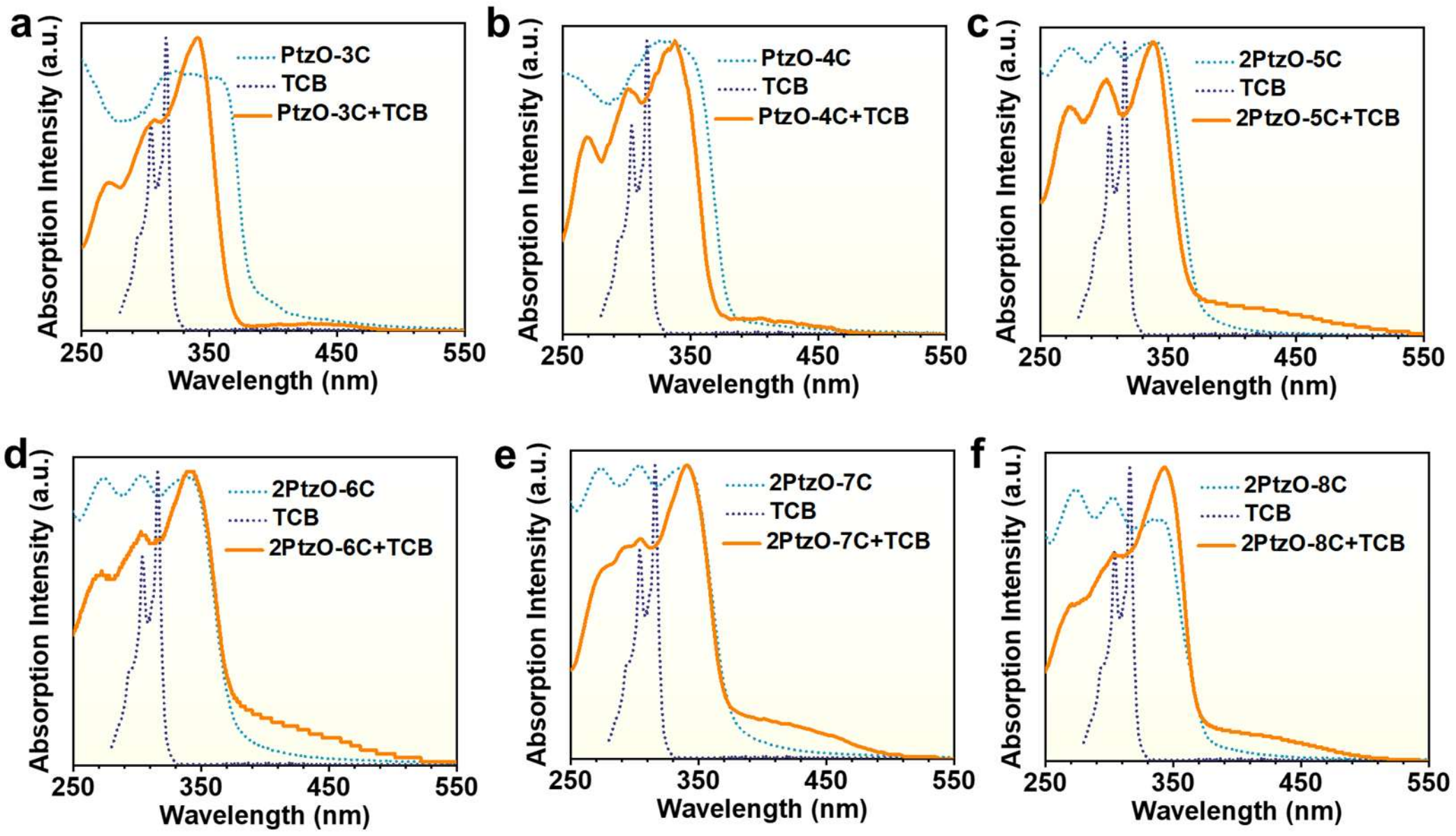

Fig. S40. a) UV-Vis absorption spectra of PtzO-3C+TCB, PtzO-3C in solid state and TCB in DCM solution. b) UV-Vis absorption spectra of PtzO-4C+TCB, PtzO-4C in solid state and TCB in DCM solution. c) UV-Vis absorption spectra of 2PtzO-5C+TCB, 2PtzO-5C in solid state and TCB in DCM solution. d) UV-Vis absorption spectra of 2PtzO-6C+TCB, 2PtzO-6C in solid state and TCB in DCM solution. e) UV-Vis absorption spectra of 2PtzO-7C+TCB, 2PtzO-7C in solid state and TCB in DCM solution. f) UV-Vis absorption spectra of 2PtzO-8C+TCB, 2PtzO-8C in solid state and TCB in DCM solution.

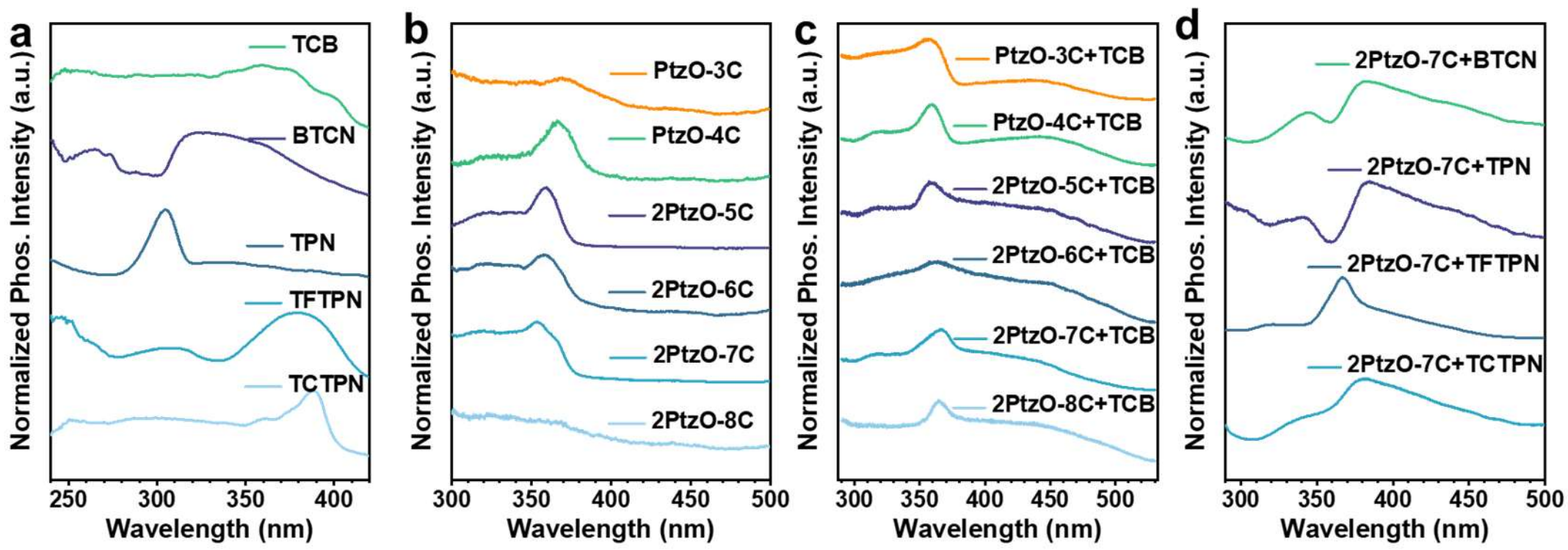

Fig. S41. a) Phosphorescence excitation spectra of TCB, BTCN, TPN, TFTPN, TCTPN in DCM at 77 K. b) Phosphorescence excitation spectra of PtzO-3C, PtzO-4C, 2PtzO-5C, 2PtzO-6C, 2PtzO-7C and 2PtzO-8C molten solids at room temperature. c) Excitation spectra for OLPL emission of PtzO-3C+TCB, PtzO-4C+TCB, 2PtzO-5C+TCB, 2PtzO-6C+TCB, 2PtzO-7C+TCB and 2PtzO-8C+TCB. d) Excitation spectra for OLPL emission of 2PtzO-7C+BTCN, 2PtzO-7C+TPN, 2PtzO-7C+TFTPN and 2PtzO-7C+TCTPN.

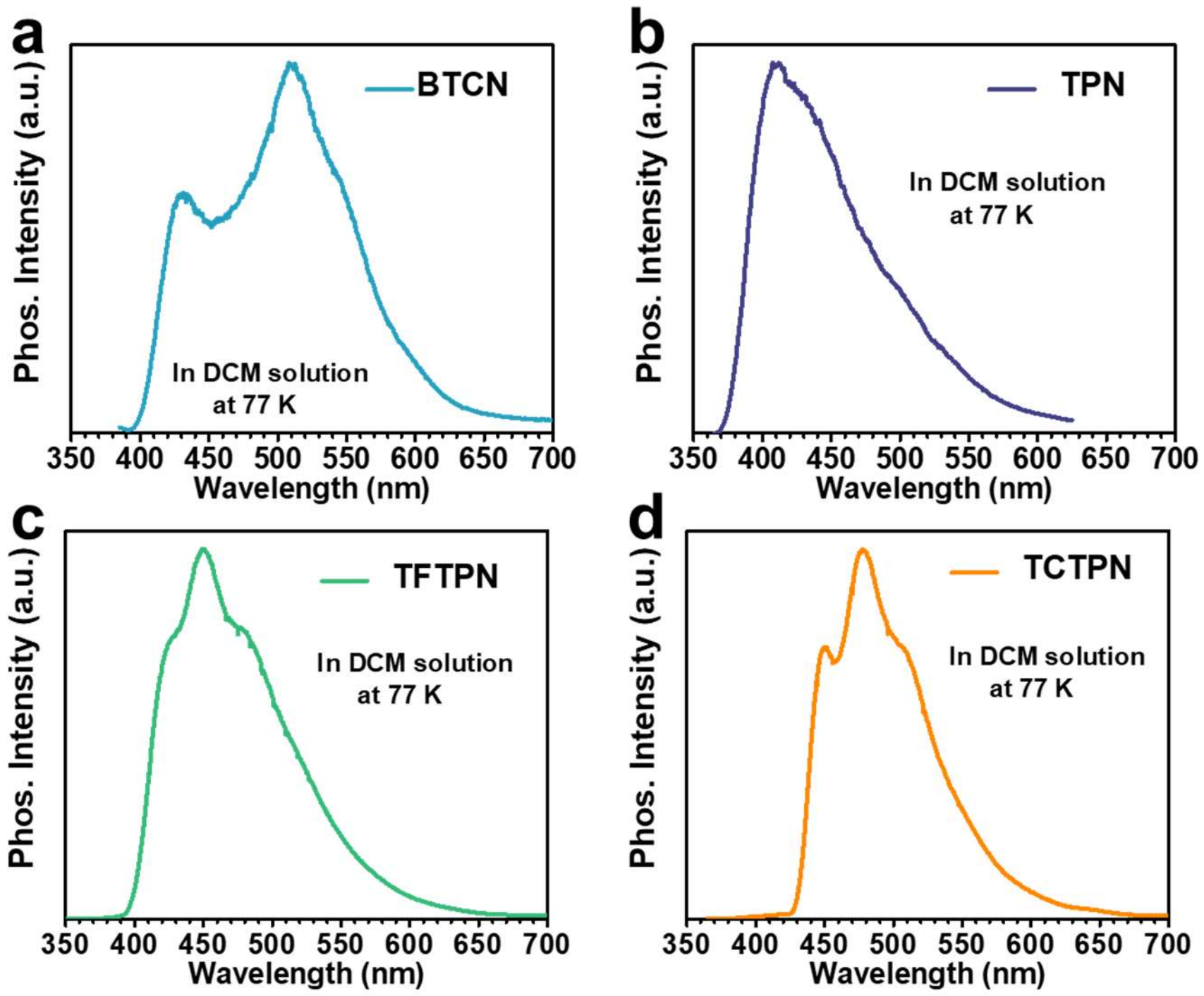

Fig. S42. a) Phosphorescence spectra of BTCN, b) TPN, c) TFTPN and d) TCTPN in DCM solution at 77 K (10^{-5} M).

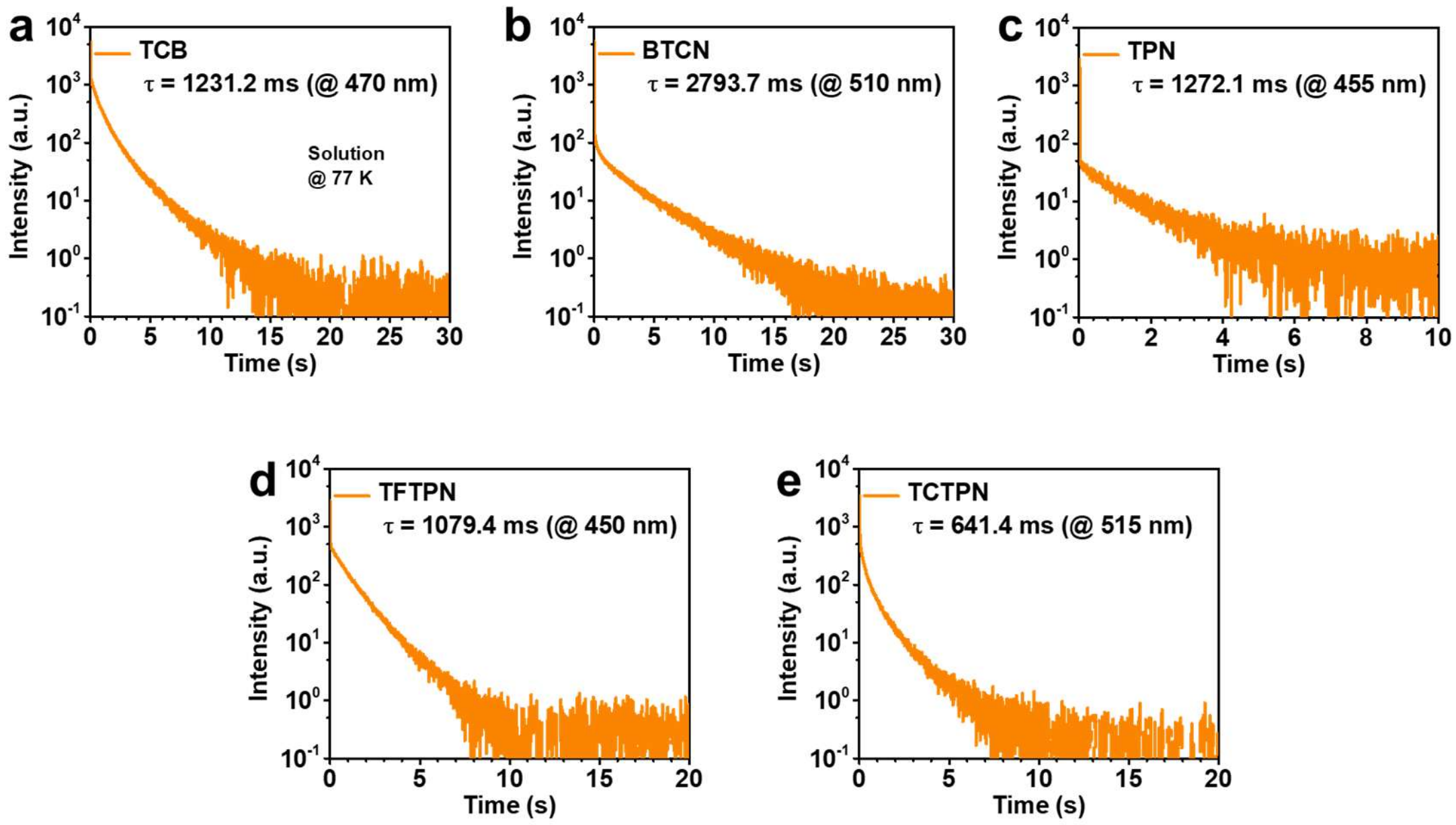

Fig. S43. a) Time-resolved phosphorescence decay curves of TCB, b) BTCN, c) TPN, d) TFTPN and c) TCTPN in DCM solution at 77 K (10^{-5} M).

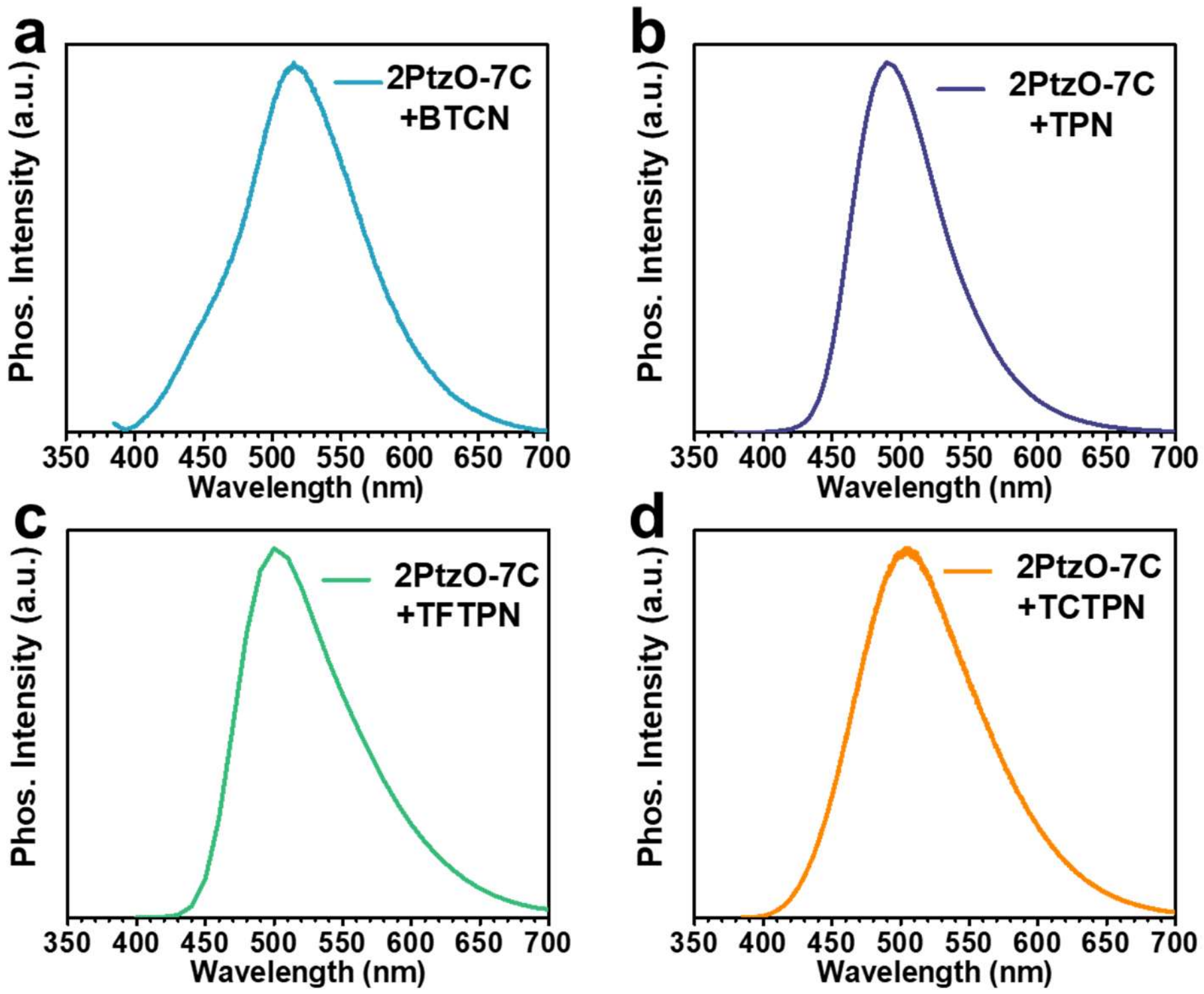

Fig. S44. a) Phosphorescence spectra of BTCN, b) TPN, c) TFTPN and d) TCTPN melted in 2PtzO-7C at room temperature (100:1).

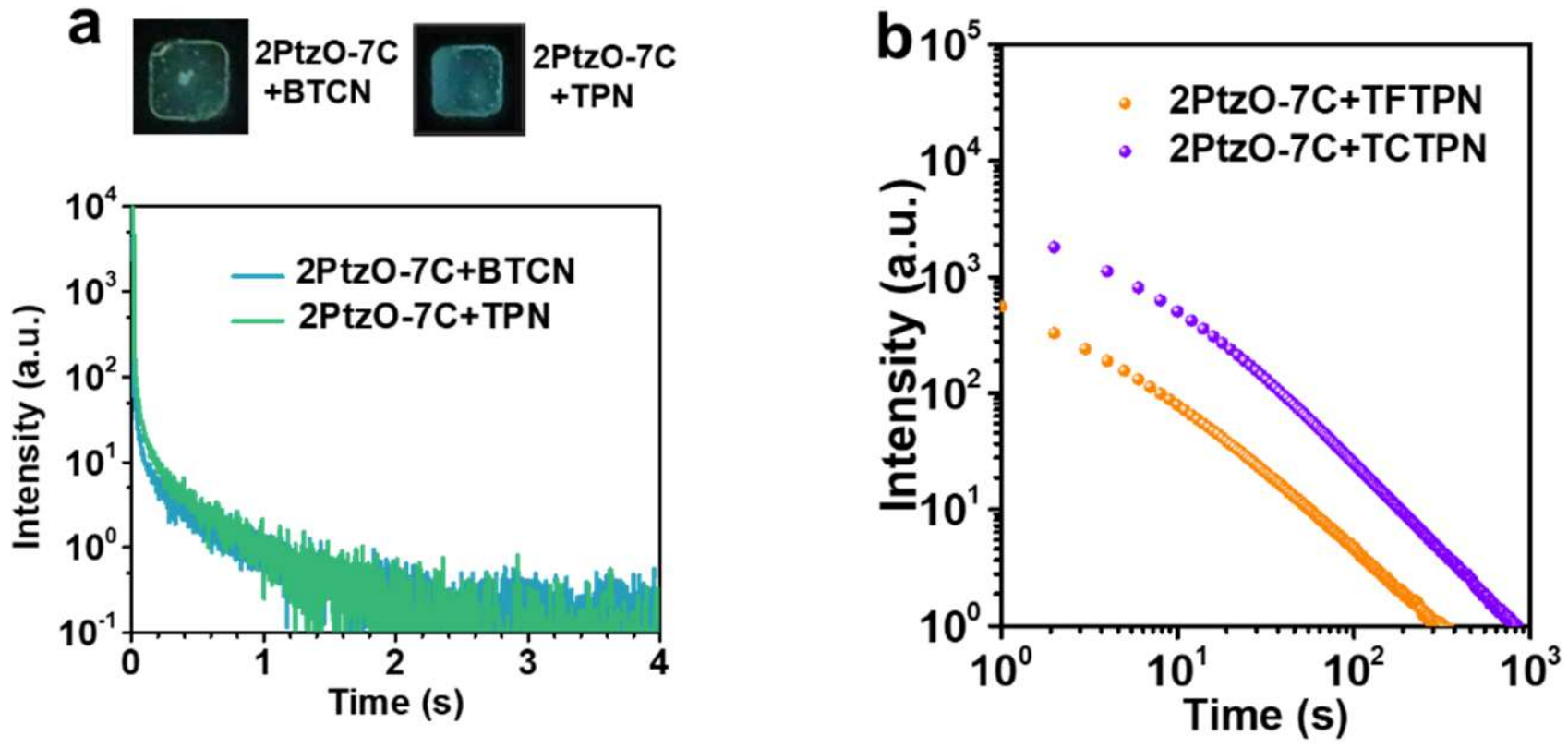

Fig. S45. a) Afterglow photographs of 2PtzO-7C+BTCN and 2PtzO-7C+TPN, and corresponding time-resolved afterglow-decay curves at room temperature. b) Time-resolved OLPL-decay curves of 2PtzO-7C+TFTPN and 2PtzO-7C+TCTPN at atmosphere (100:1).

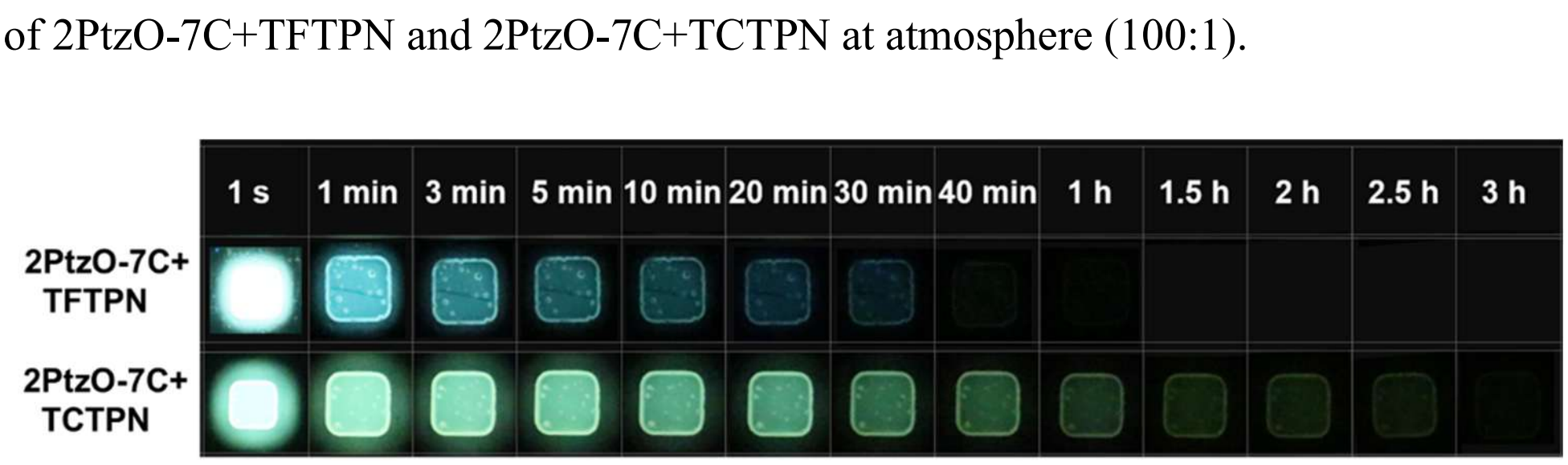

Fig. S46. Photographs of 2PtzO-7C+TFTPN and 2PtzO-7C+TCTPN taken under air by Canon camera after the removal of 365 nm UV lamp for different times.

Table S5. OLPL, ESR and energy data of five kinds of molten mixtures and corresponding acceptors.

	2PtzO-7C +TCB	2PtzO-7C +BTCN	2PtzO-7C +TPN	2PtzO-7C +TFTPN	2PtzO-7C +TCTPN
Φ	23.01	4.19	3.23	18.96	19.97
t(s)[a]	7154.3	4.6	1.6	398.1	1096.5
I (a.u.)	538	206	155	385	414
⊿E (eV)	2.26	1.08	0.77	1.52	1.81
	TCB	BTCN	TPN	TFTPN	TCTPN
t(s)[b]	1584.9	79.4	30.2	1318.3	2187.7

Φ = PLQY detected at room temperature;
t(s) [a] = duration time detected at room temperature;
I (a.u.) = maximum ESR intensity of molten mixtures detected at room temperature;
⊿E (eV) = energy gap between neutral molecule and radical of corresponding acceptors;
t(s) [b] = duration time of guests doped in PMMA detected at 77 K.

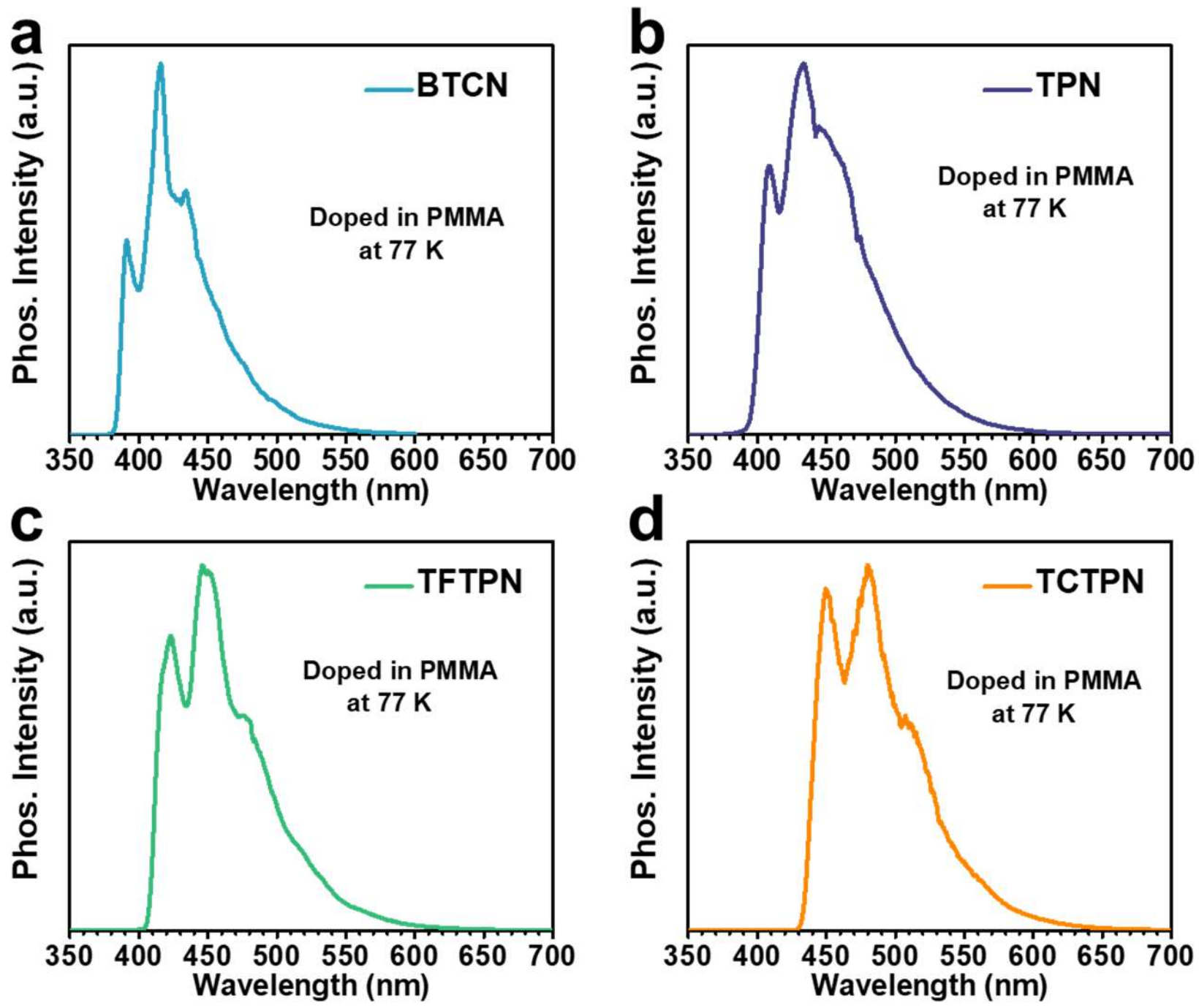

Fig. S47. a) Phosphorescence spectra of BTCN, b) TPN, c) TFTPN and d) TCTPN doped in PMMA at 77 K (10^2: 1).

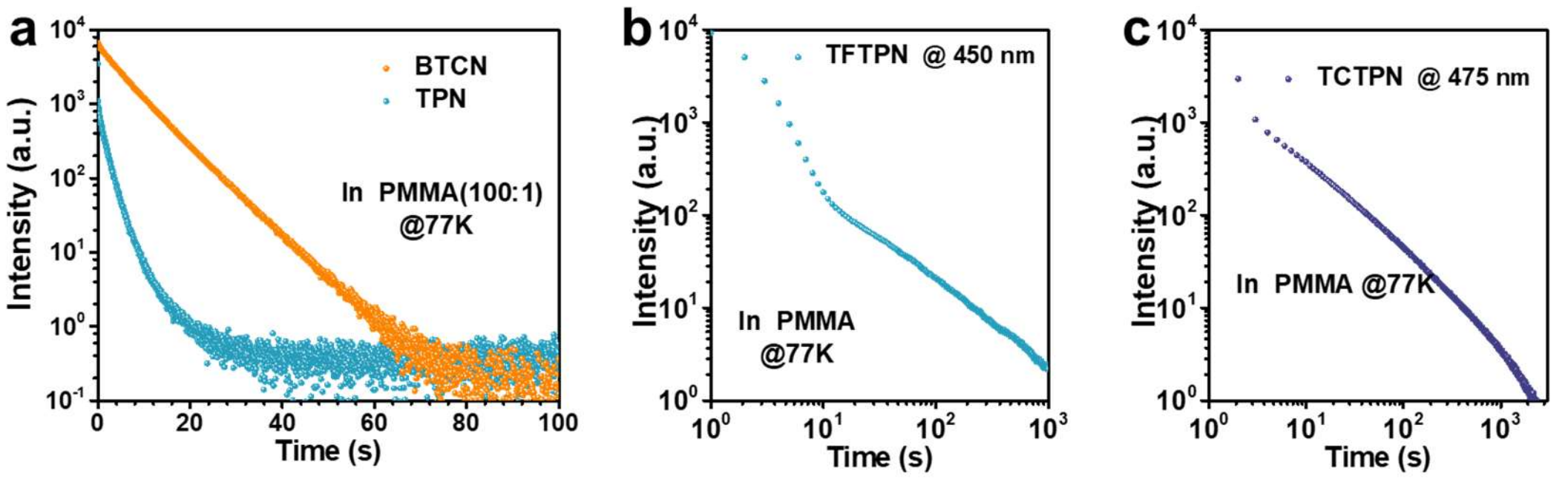

Fig. S48. a) Time-resolved afterglow-decay curves of BTCN and TPN, b) TFTPN and c) TCTPN doped in PMMA at 77 K.

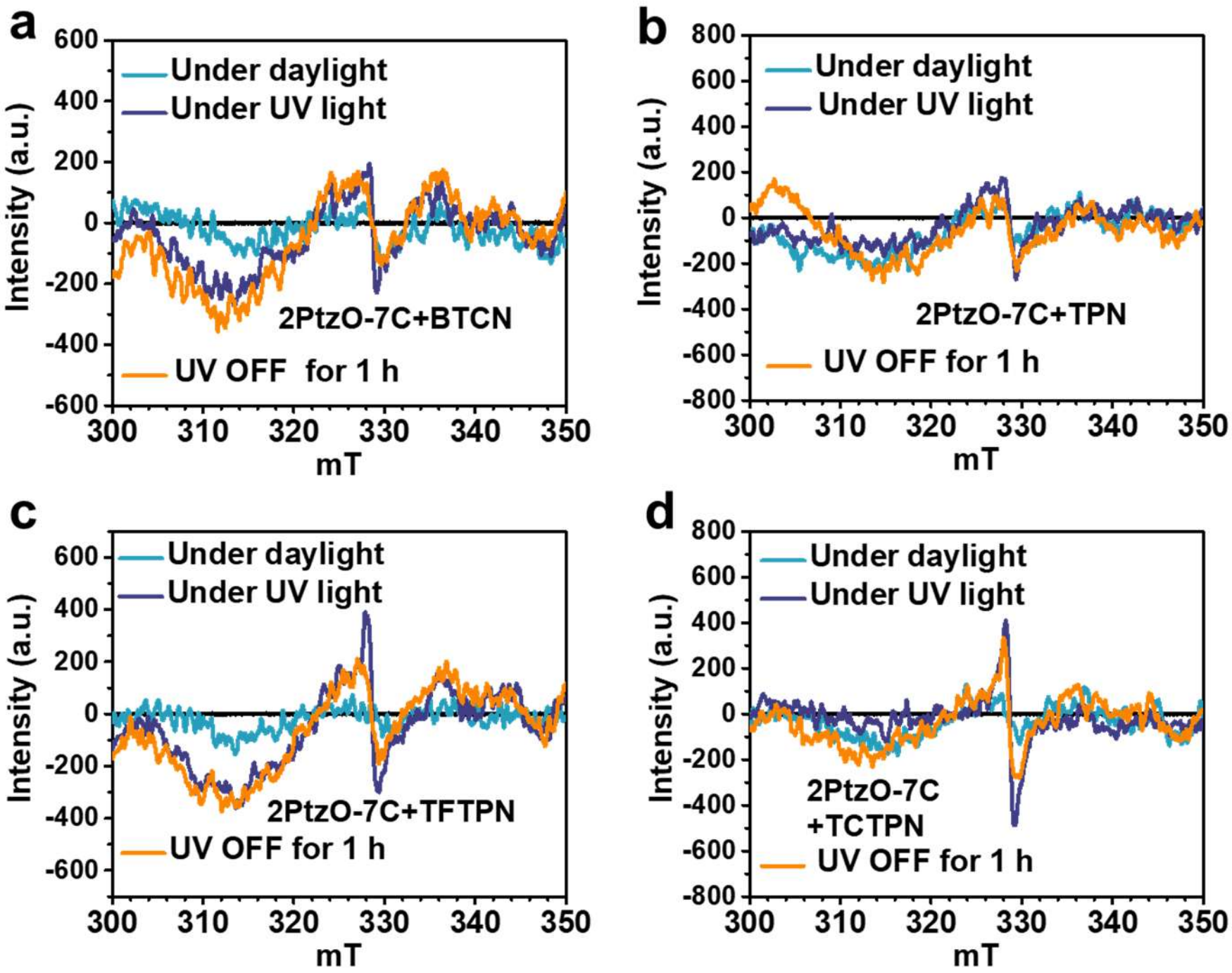

Fig. S49. a) ESR spectra of 2PtzO-7C+BTCN, b) 2PtzO-7C+TPN, c) 2PtzO-7C+TFTPN, d) 2PtzO-7C+TCTPN before, under and after the UV excitation at room temperature.

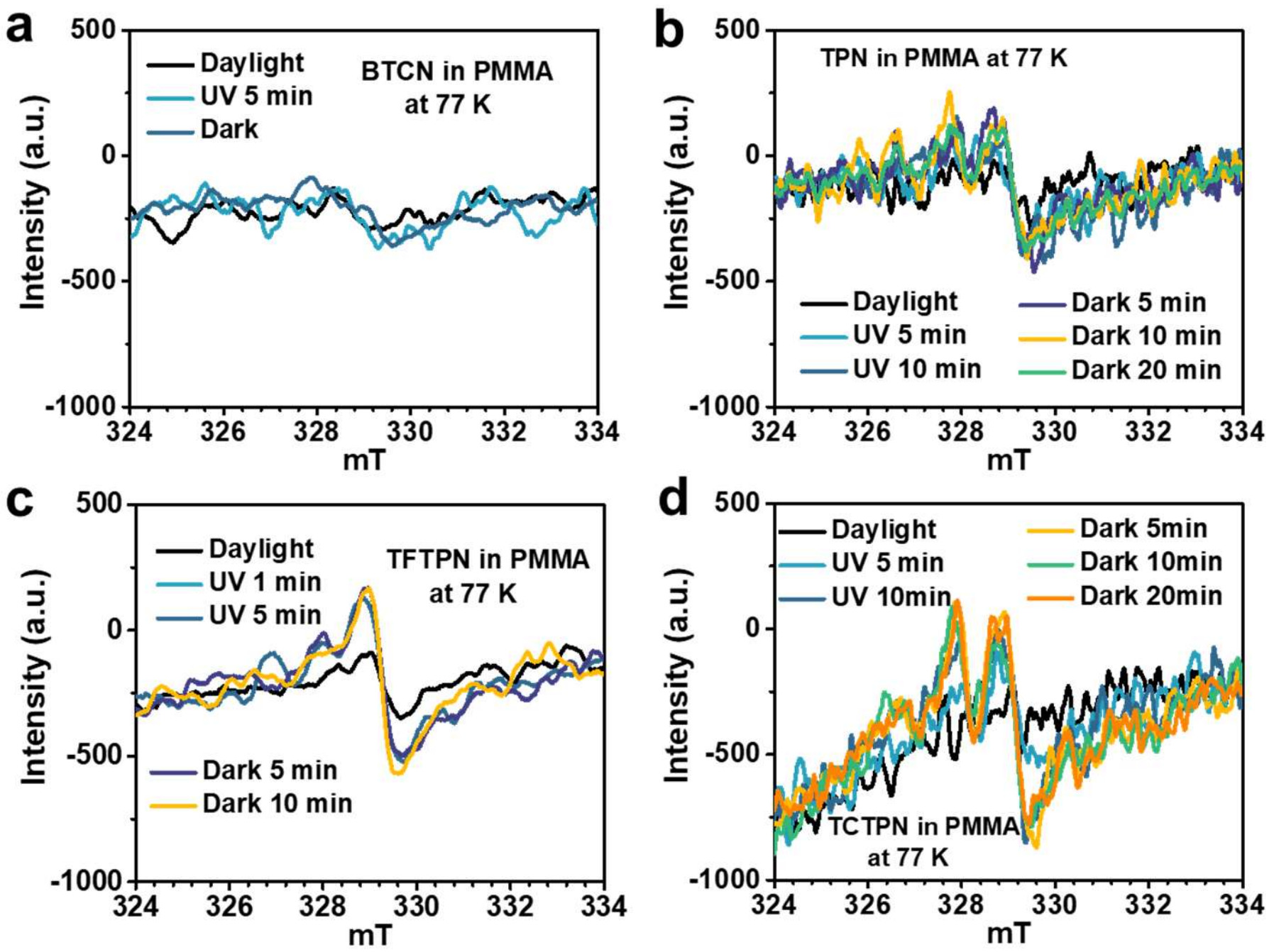

Fig. S50. a) ESR spectra of BTCN, b) TPN, c) TFTPN, d) TCTPN doped in PMMA before, under and after the UV excitation at 77 K.

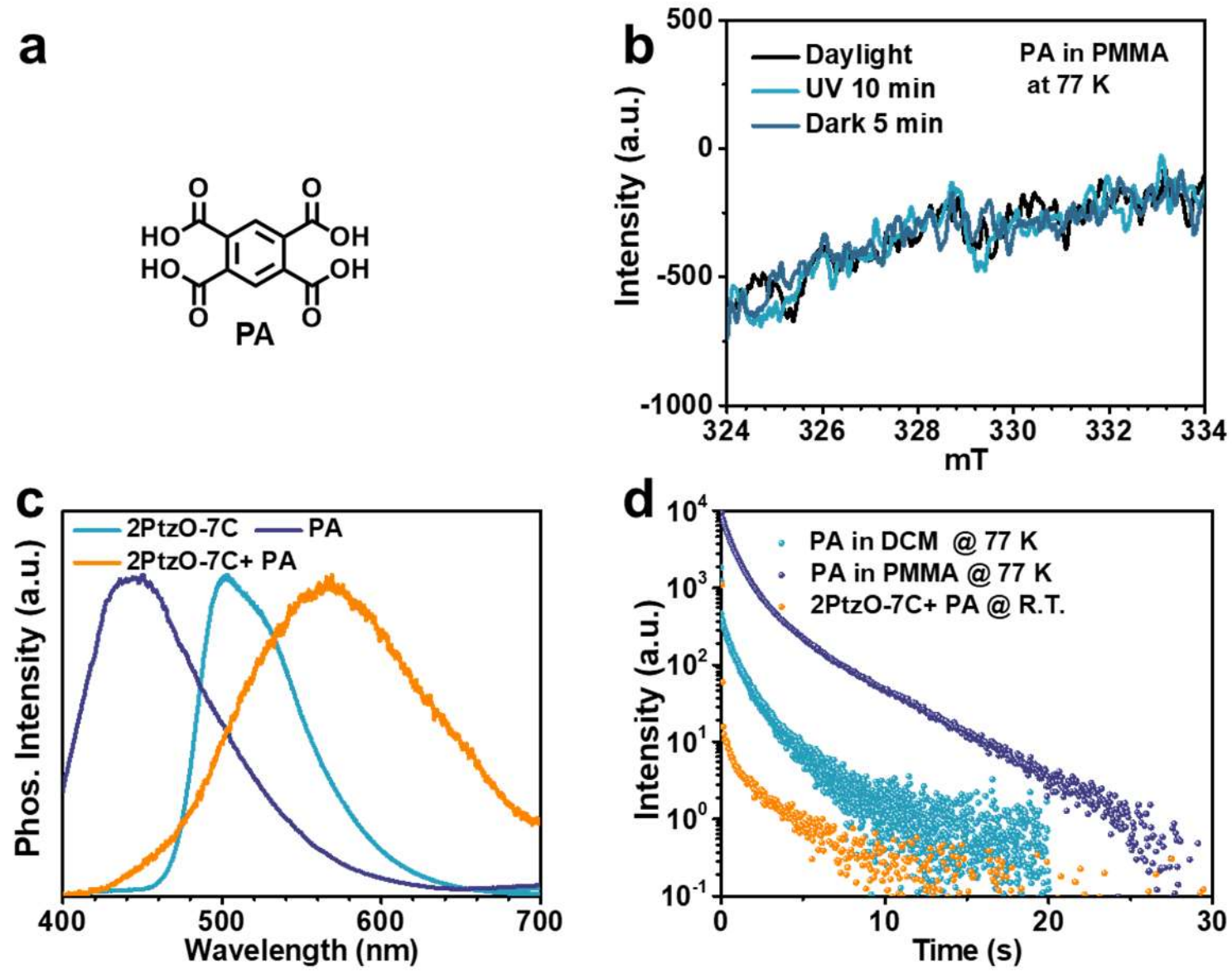

Fig. S51. a) Chemical structure of PA. b) ESR spectra of PA doped in PMMA at 77 K before, under and after the UV excitation. c) Afterglow emission spectra of 2PtzO-7C in solid state, PA in DCM solution at 77 K and 2PtzO-7C+PA at room temperature. d) Time-resolved afterglow decay curves of PA in different states.

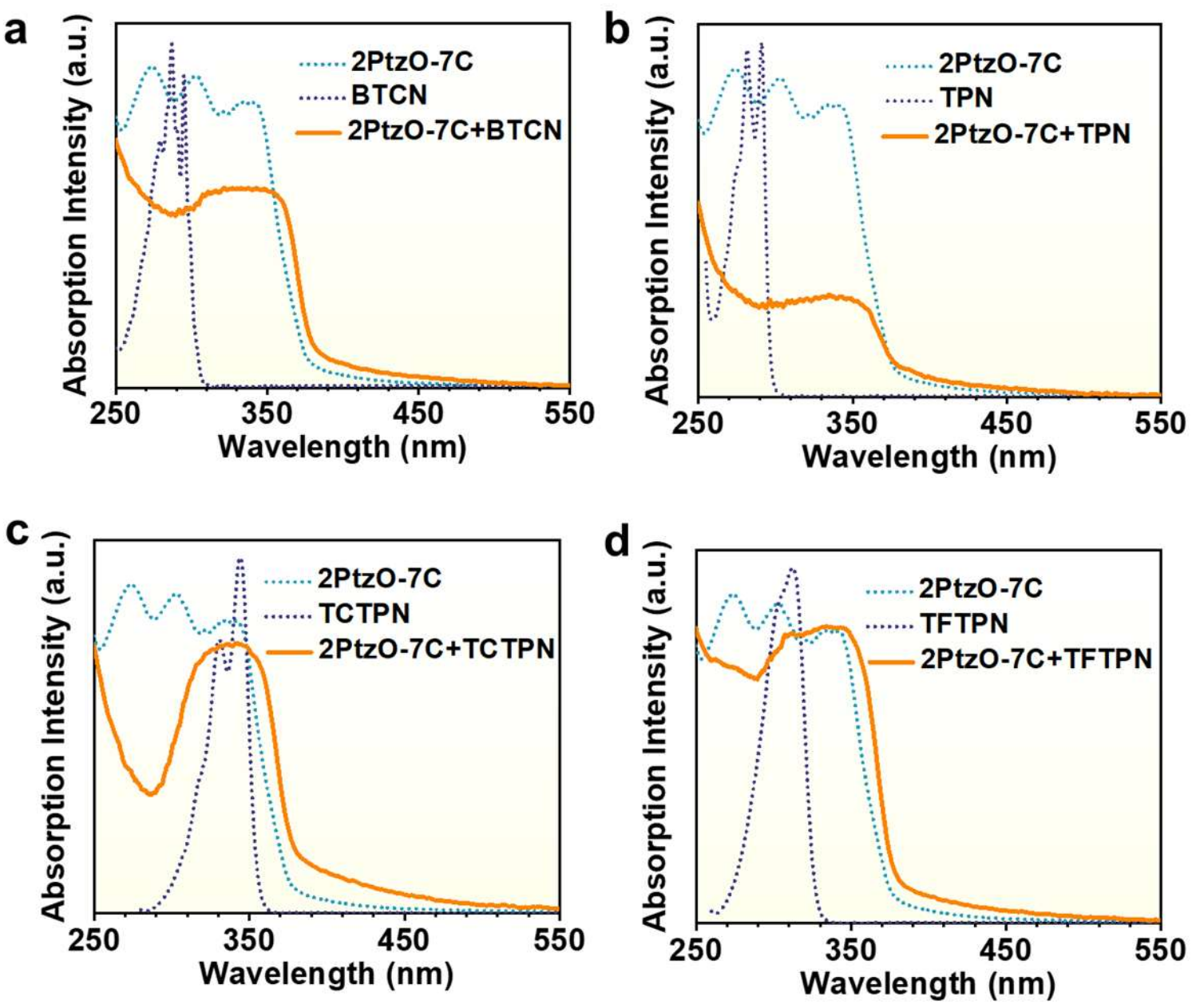

Fig. S52. a) UV-Vis absorption spectra of 2PtzO-7C+BTCN, 2PtzO-7C in solid state and BTCN in DCM solution. b) UV-Vis absorption spectra of 2PtzO-7C+TPN, 2PtzO-7C in solid state and TPN in DCM solution. c) UV-Vis absorption spectra of 2PtzO-7C+TCTPN, 2PtzO-7C in solid state and TCTPN in DCM solution. d) UV-Vis absorption spectra of 2PtzO-7C+TFTPN, 2PtzO-7C in solid state and TFTPN in DCM solution.

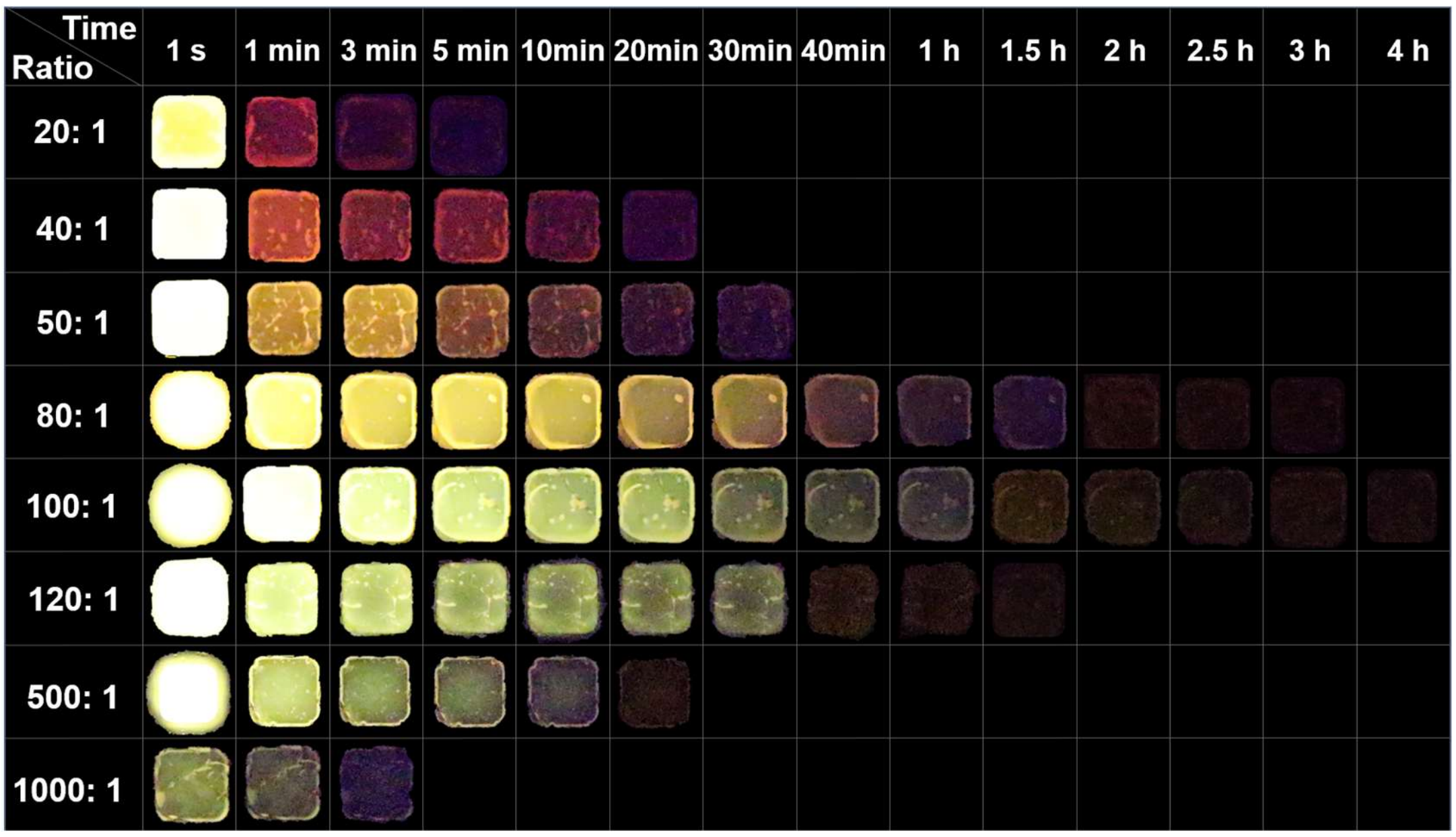

Fig. S53. Photographs of OLPL emission excited by 365 nm UV lamp from 2PtzO-7C+TCB with different ratios.

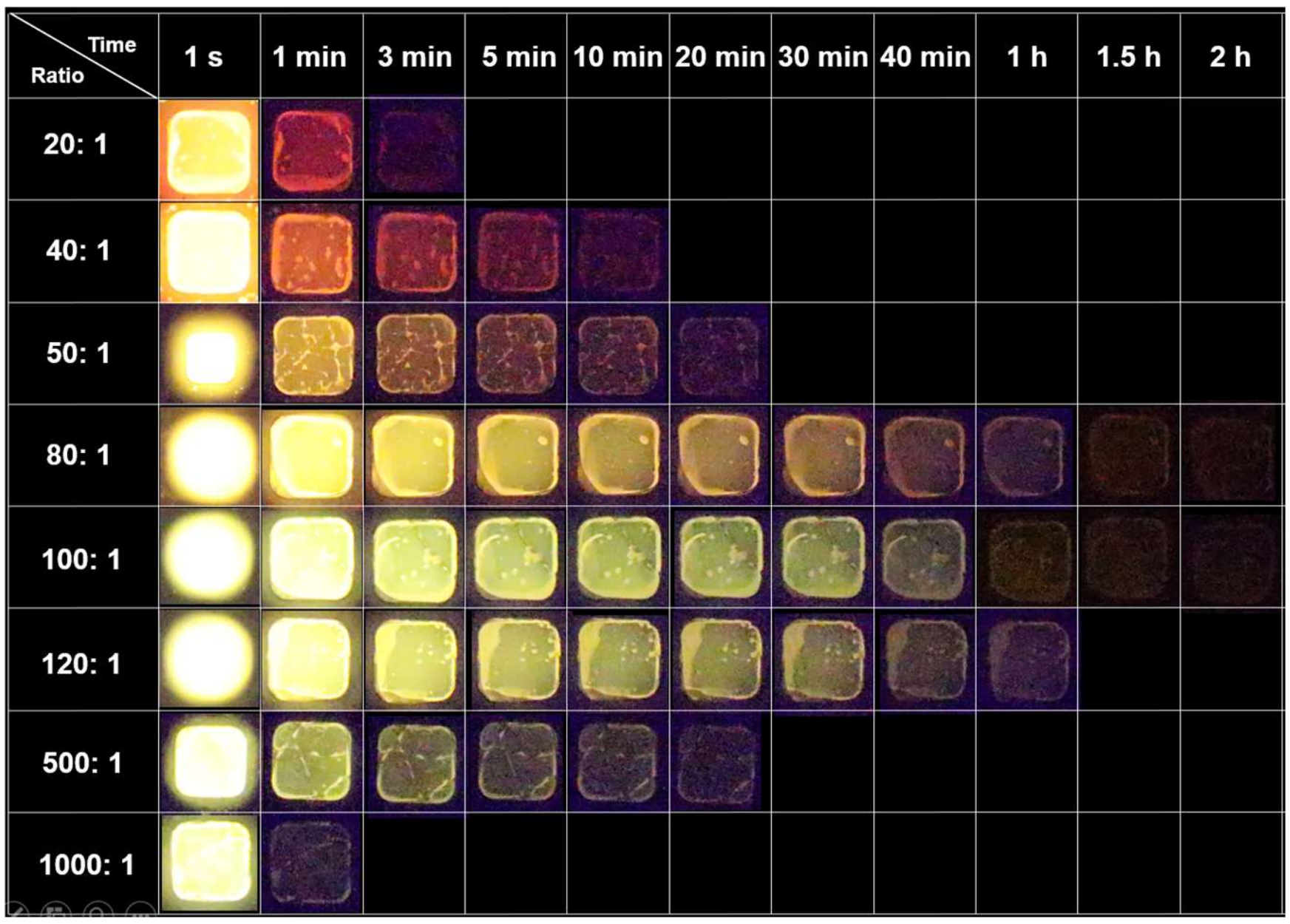

Fig. 54. Photographs of OLPL emission excited by flashlight of i-Phone 13 from 2PtzO-7C+TCB with different ratios.

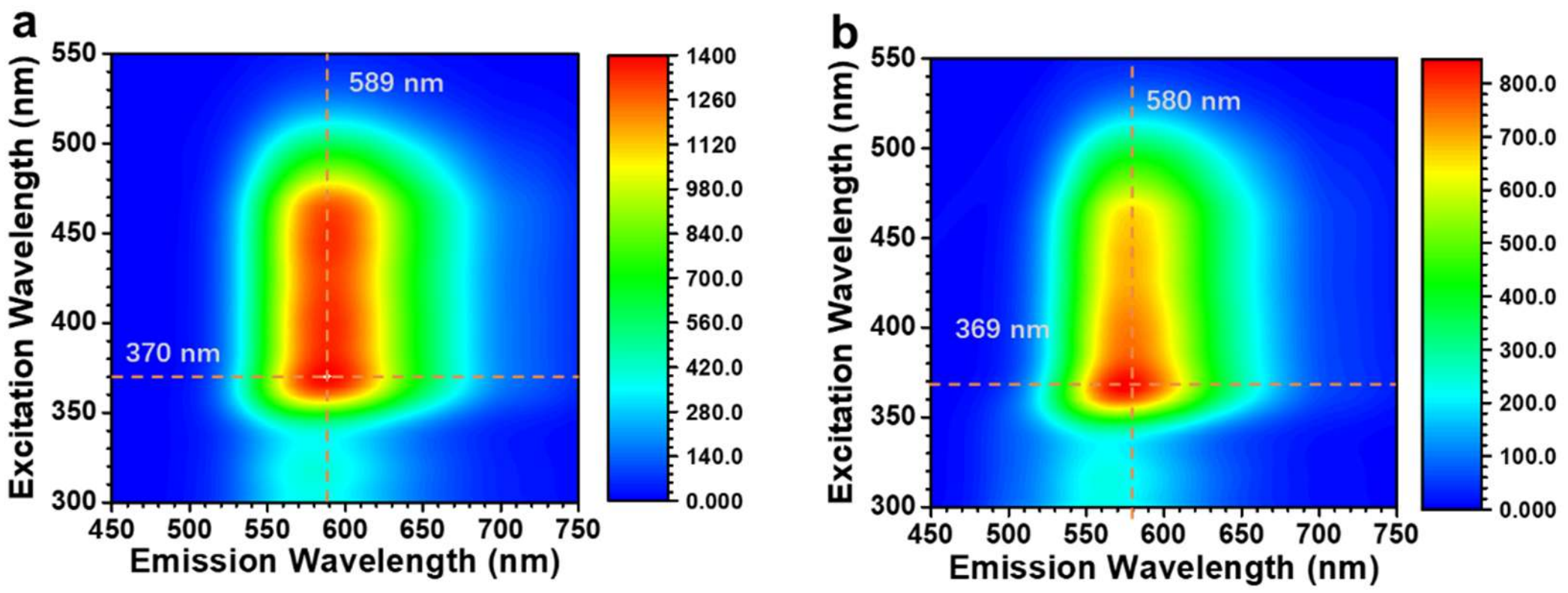

Fig. S55. a) Excitation-emission mapping of 2PtzO-7C+TCB (20:1). b) Excitation-emission mapping of 2PtzO-7C+TCB (40:1).

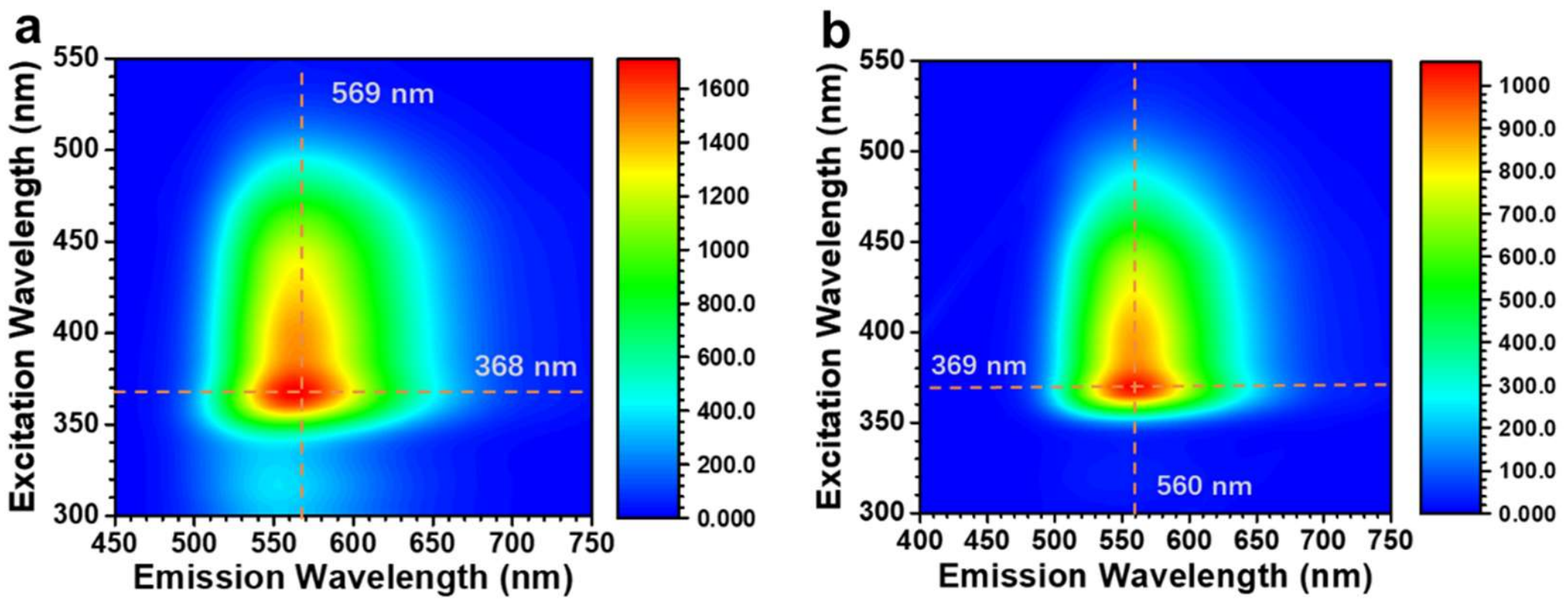

Fig. S56. a) Excitation-emission mapping of 2PtzO-7C+TCB (50:1) b) Excitation-emission mapping of 2PtzO-7C+TCB (80:1).

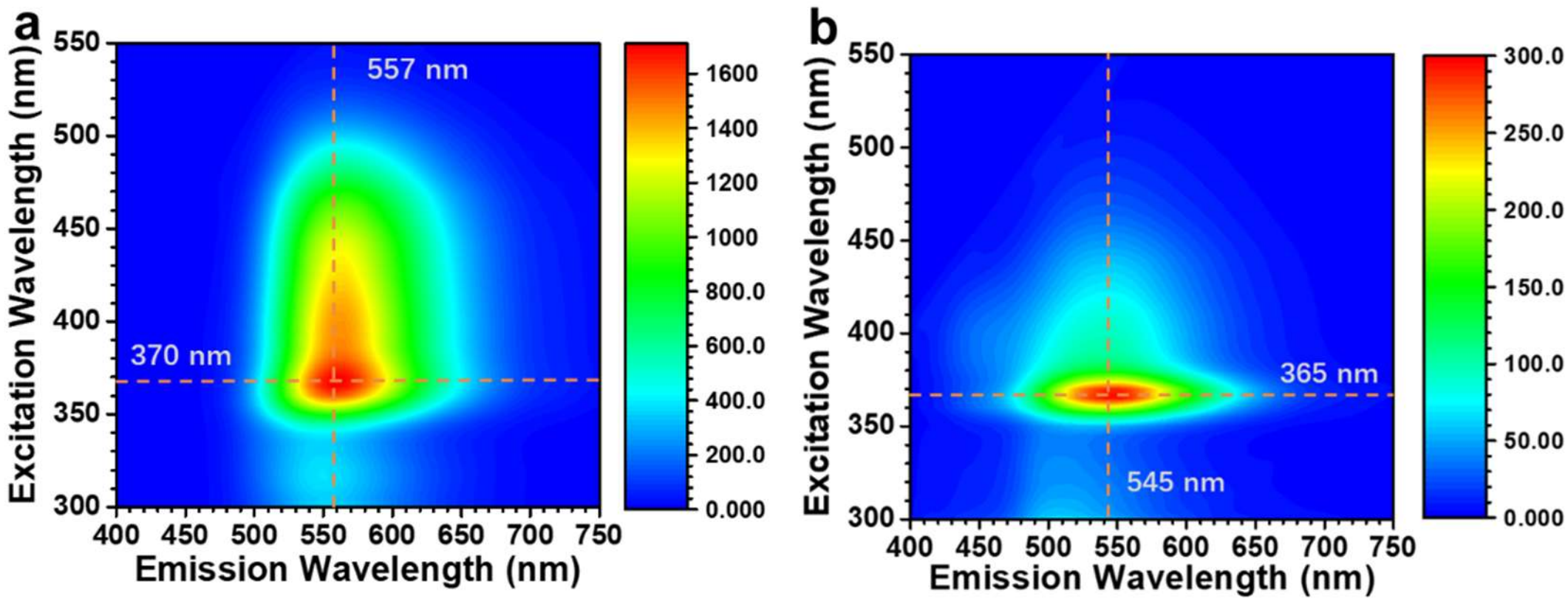

Fig. S57. a) Excitation-emission mapping of 2PtzO-7C+TCB (100:1). b) Excitation-emission mapping of 2PtzO-7C+TCB (120:1).

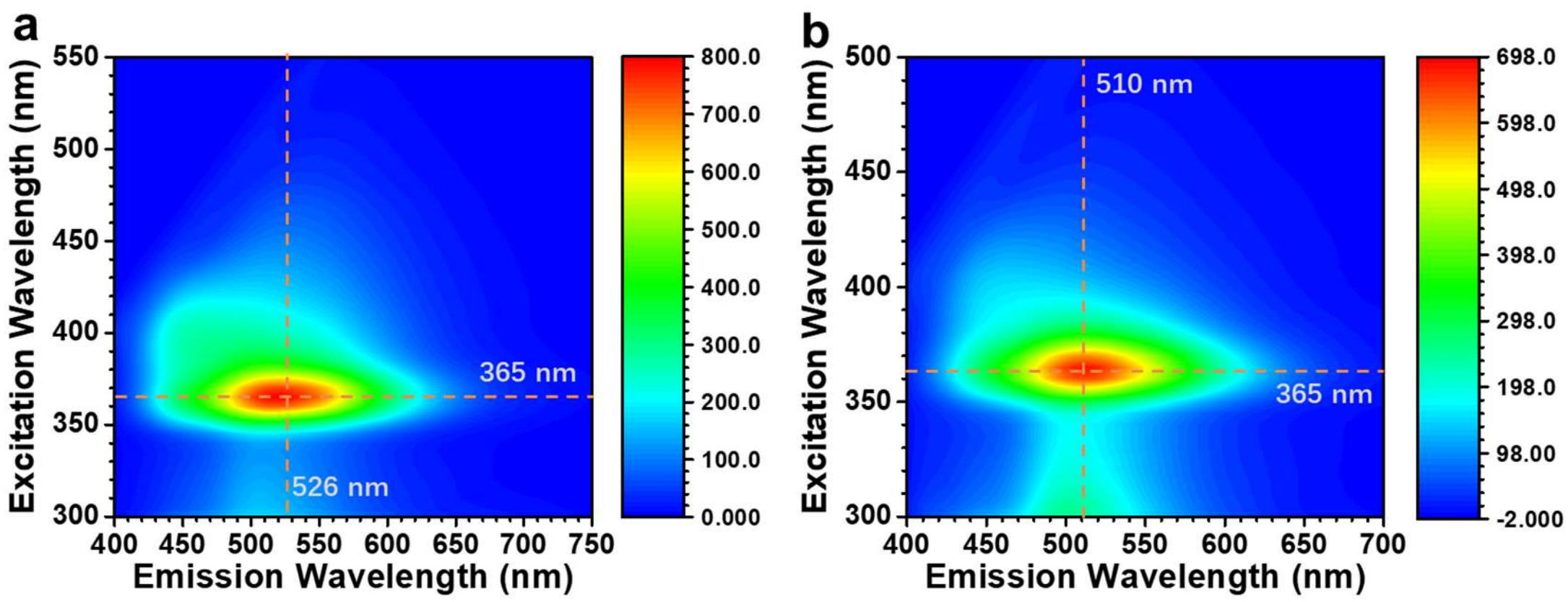

Fig. S58. a) Excitation-emission mapping of 2PtzO-7C+TCB (500:1). b) Excitation-emission mapping of 2PtzO-7C+TCB (1000:1).

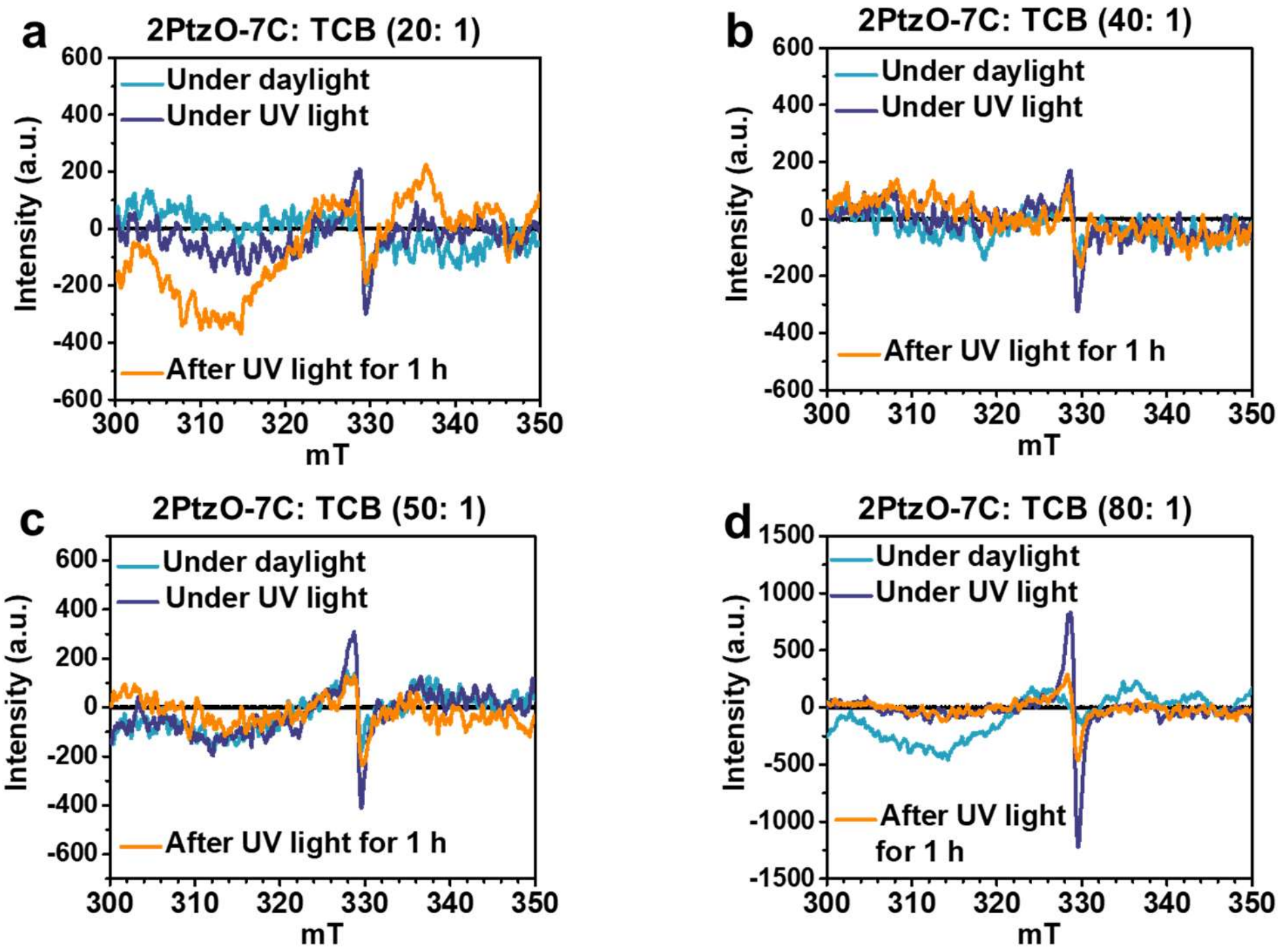

Fig. S59. a) ESR spectra of 2PtzO-7C+TCB with a ratio of 20:1, b) 40:1, c) 50:1 and d) 80:1.

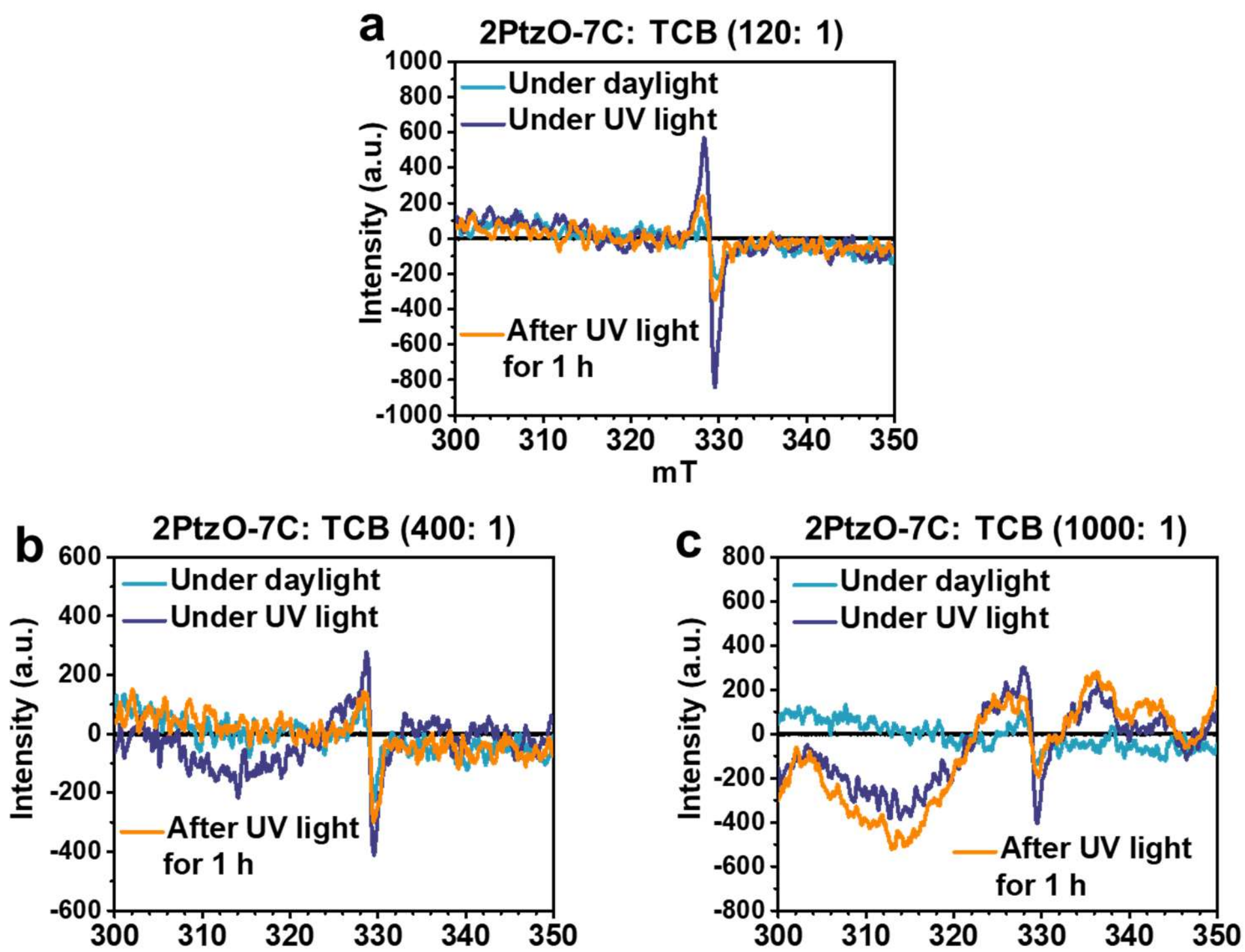

Fig. S60. a) ESR spectra of 2PtzO-7C+TCB with a ratio of 120:1, b) 40:1, c) 500:1 and d) 1000:1.

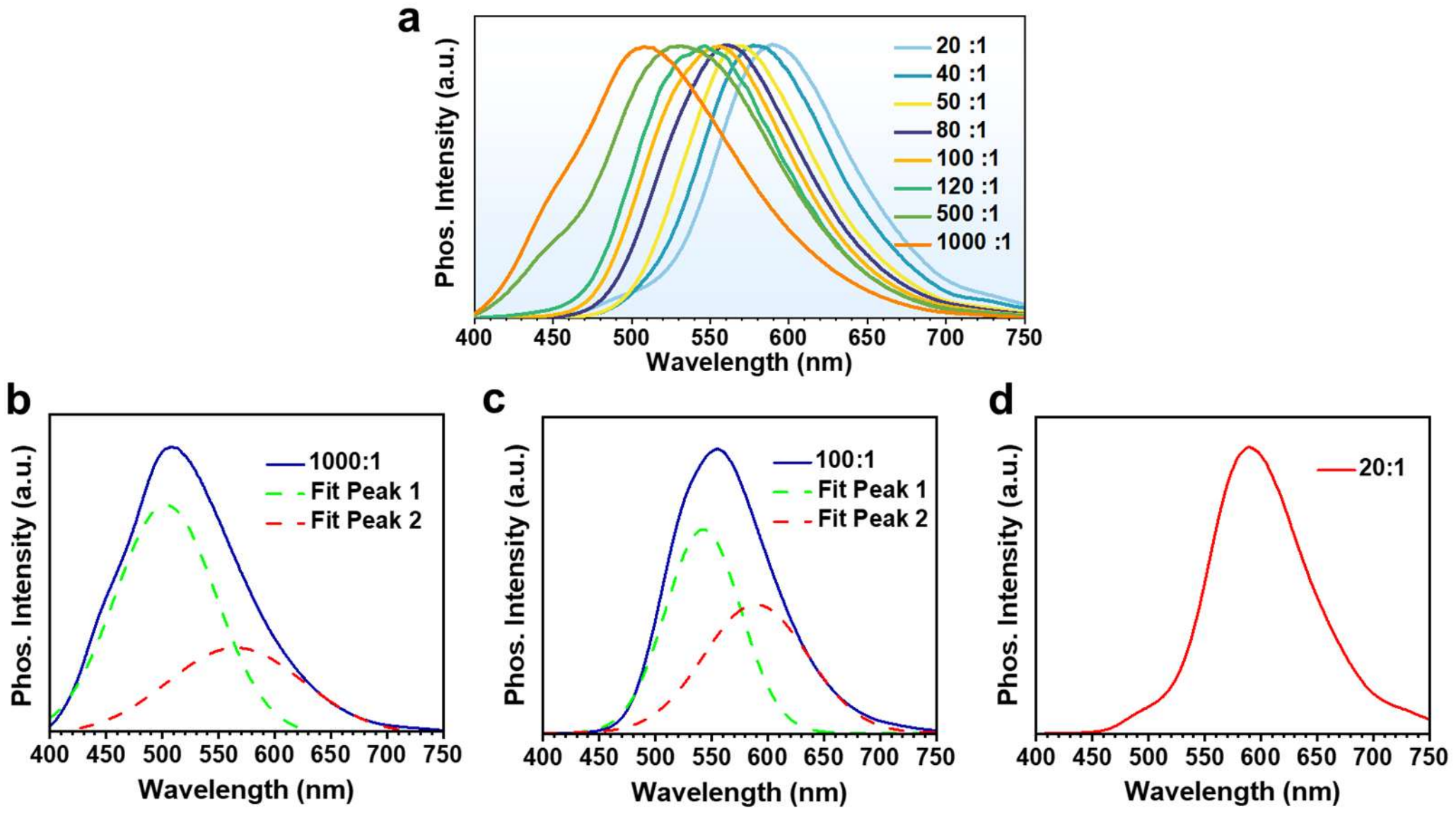

Fig. S61. a) Afterglow emission spectra of 2PtzO-7C+TCB with different ratios. b) Fitted afterglow emission spectra of 2PtzO-7C+TCB with doping ratio of 1000:1. c) Fitted afterglow emission spectra of 2PtzO-7C+TCB with doping ratio of 100:1. d) Afterglow emission spectrum of 2PtzO-7C+TCB with doping ratio of 20:1.

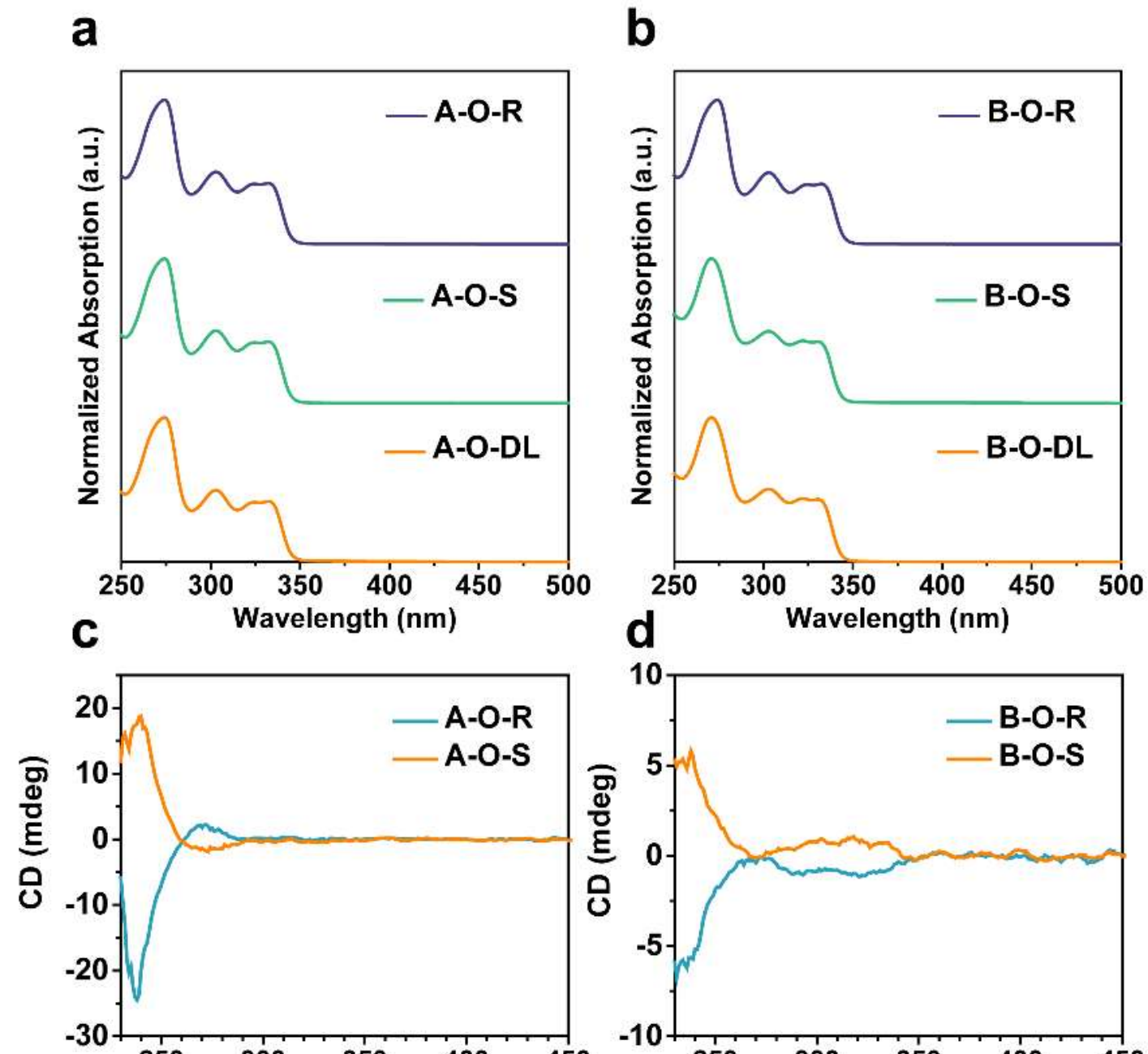

Fig. S62. a) UV-Vis absorption spectra of A-O-S/R/DL. b) UV-Vis absorption spectra of B-O-S/R/DL. c) CD spectra of A-O-S/R/DL. d) CD spectra of B-O-S/R/DL in solution at room temperature.

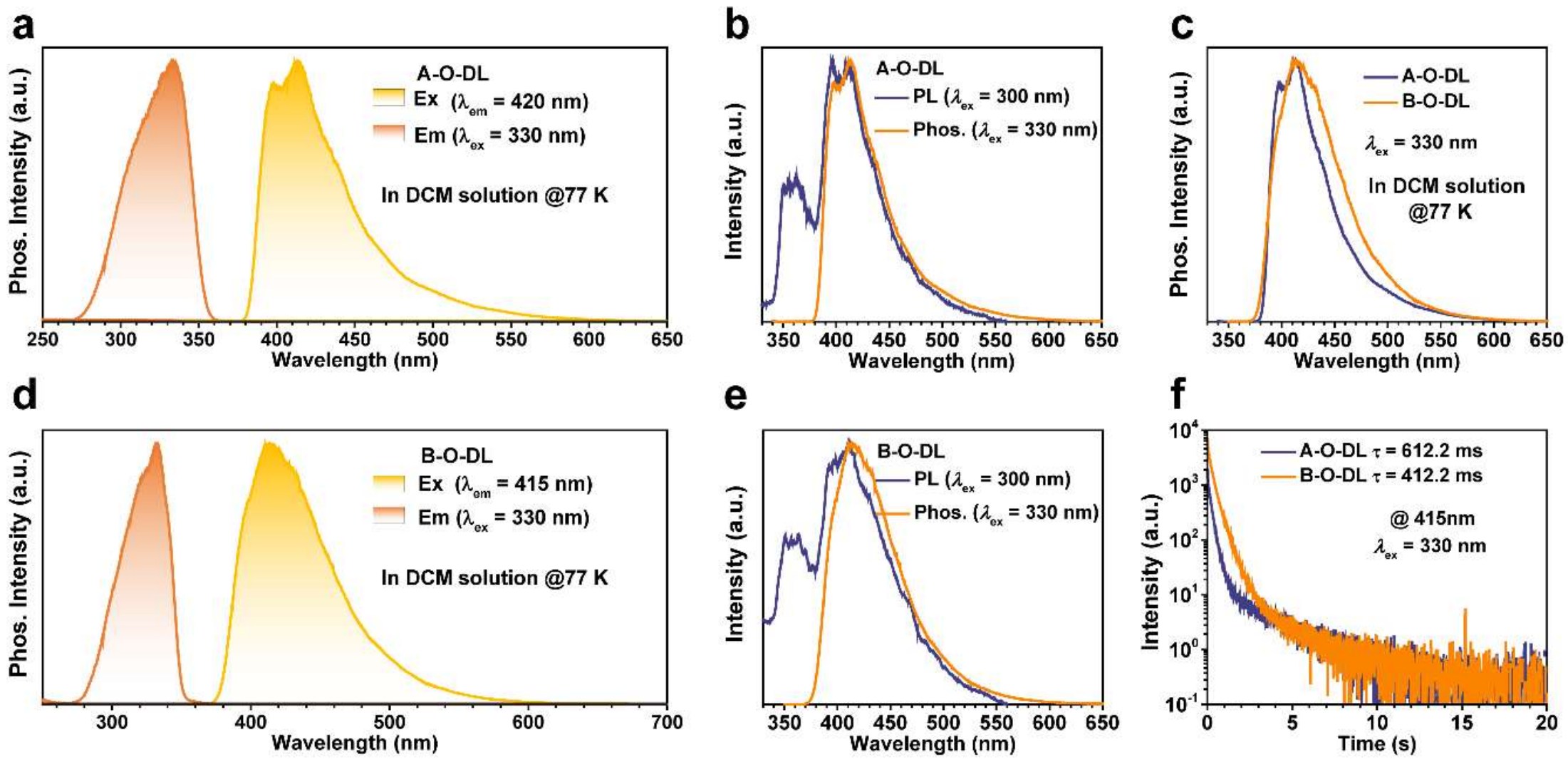

Fig. S63. a) Phosphorescence excitation and emission spectra of A-O-DL in DCM solution at 77 K. b) Steady-state photoluminescence and phosphorescence spectra of A-O-DL in DCM solution at 77 K. c) Comparison of phosphorescence spectra of A-O-DL and B-O-DL in DCM solution at 77 K. d) Phosphorescence excitation and emission spectra of B-O-DL in DCM solution at 77 K. e) Steady-state photoluminescence and phosphorescence spectra of B-O-DL in DCM solution at 77 K. f) Phosphorescence decay curves of A-O-DL and B-O-DL in DCM solution at 77 K.

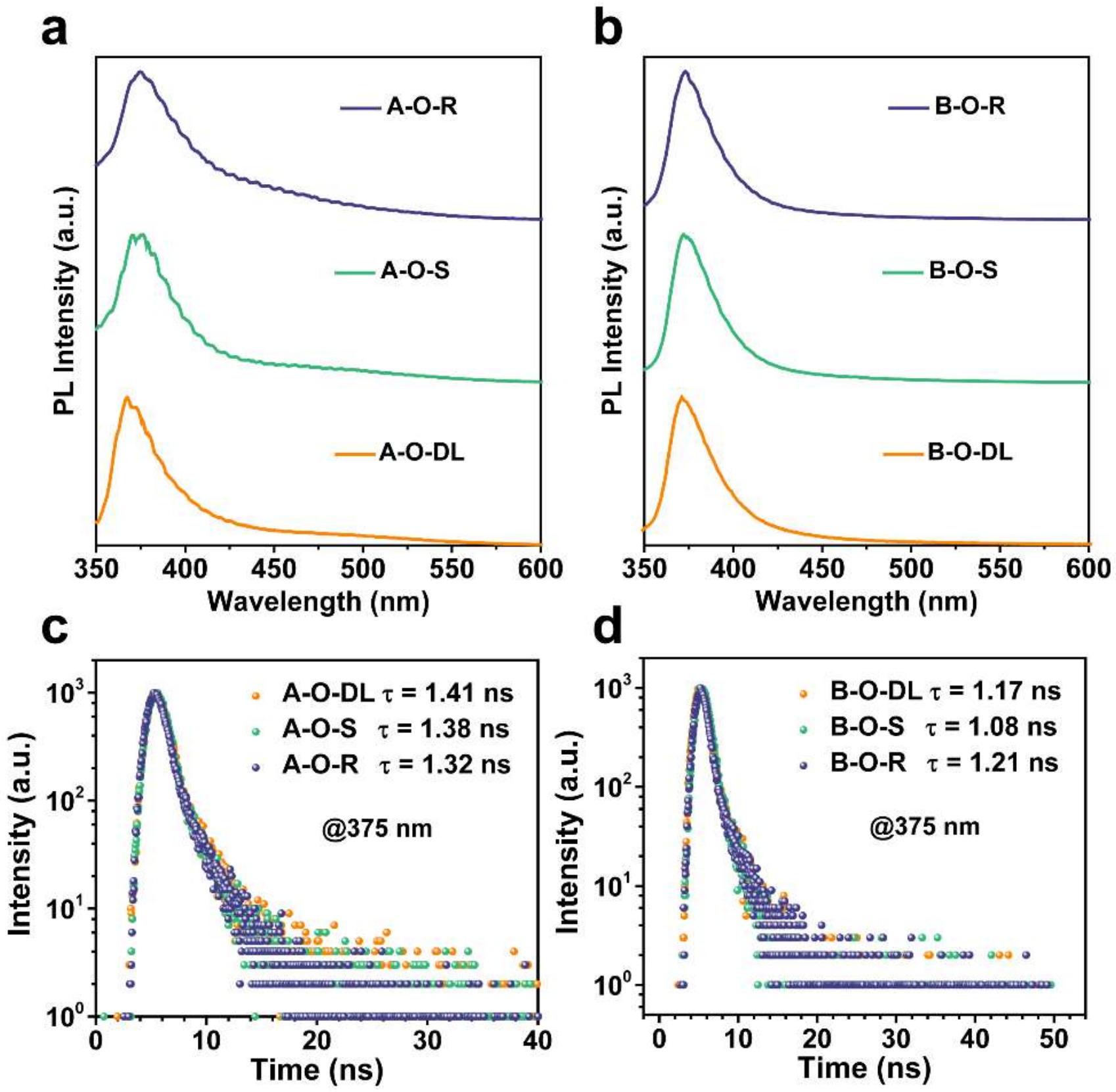

Fig. S64. a) Steady-state PL spectra of A-O-S/R/DL in the solid state. b) Steady-state PL spectra of B-O-S/R/DL in the solid state. c) Time-resolved fluorescence decay curves of A-O-S/R/DL in the solid state at 375 nm. d) Time-resolved fluorescence decay curves of B-O-S/R/DL in the solid state at 375 nm.

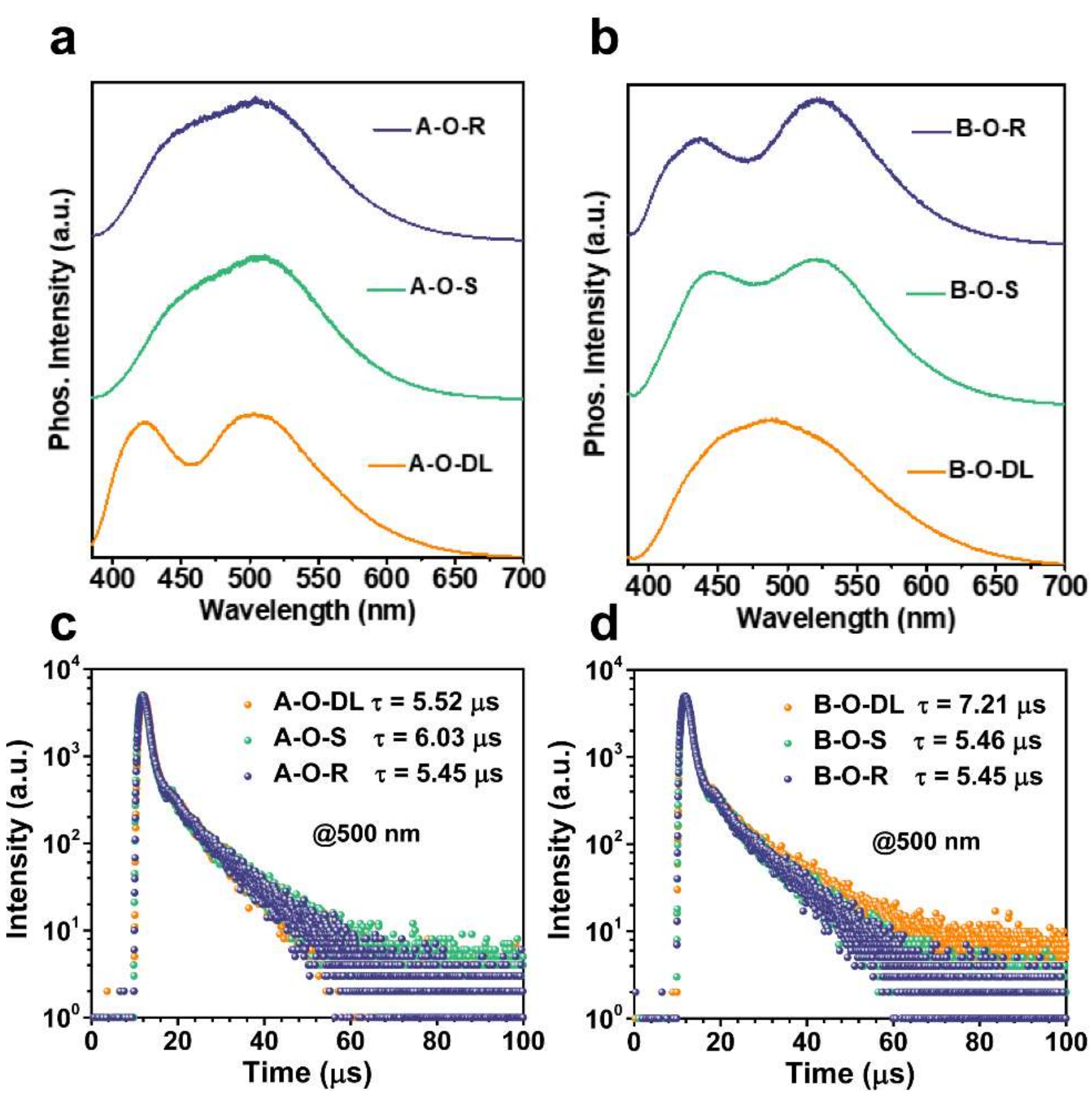

Fig. S65. a) Phosphorescence spectra of A-O-S/R/DL in the solid state at room temperature. b) Phosphorescence spectra of B-O-S/R/DL in the solid state at room temperature. c) Time-resolved phosphorescence decay curves of A-O-S/R/DL in the solid state at 500 nm. d) Time-resolved phosphorescence decay curves of B-O-S/R/DL in the solid state at 500 nm.

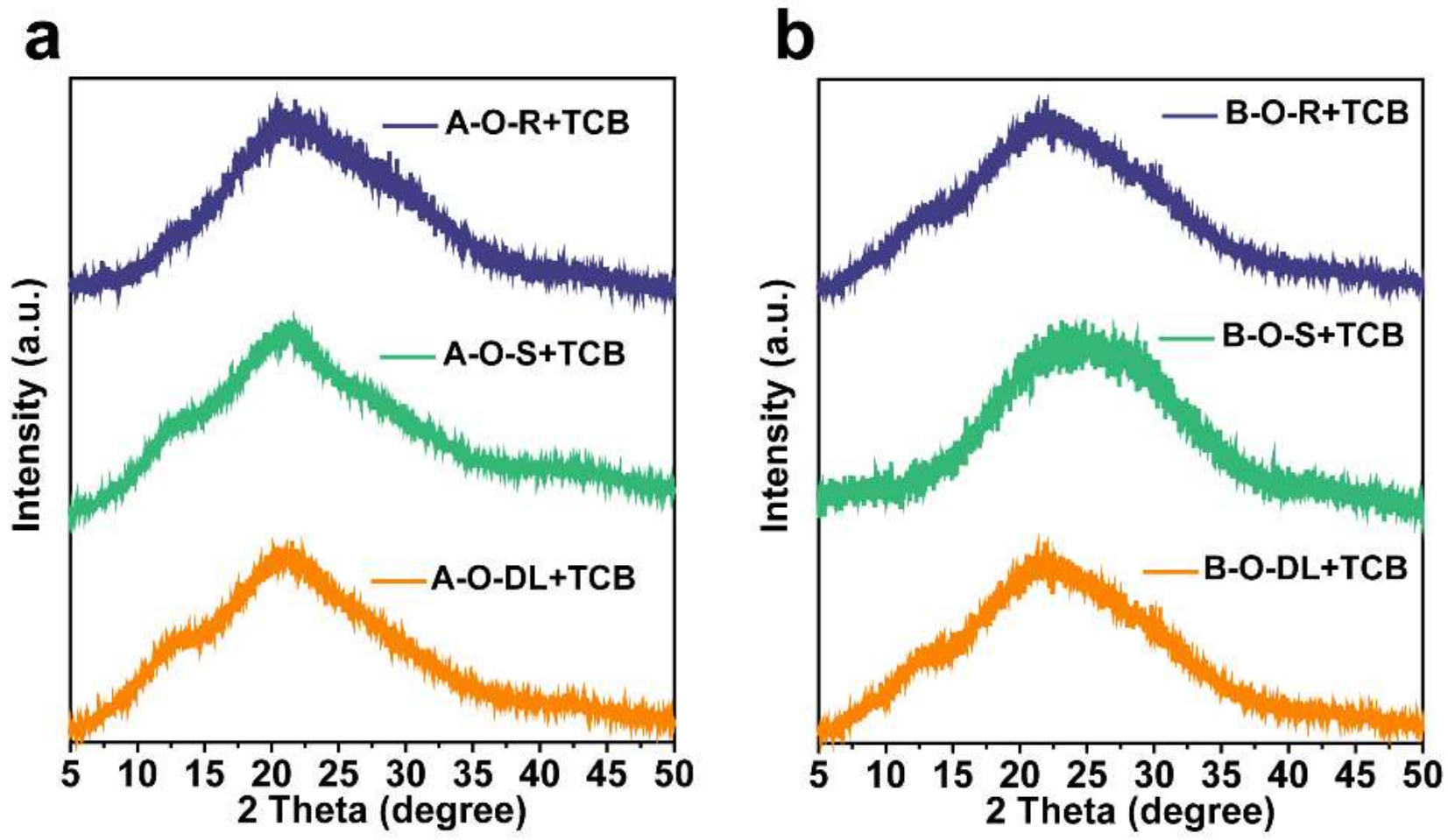

Fig. S66. Powder X-ray diffraction (PXRD) patterns of a) A-O-R/S/DL+TCB and b) B-O-R/S/DL+TCB.

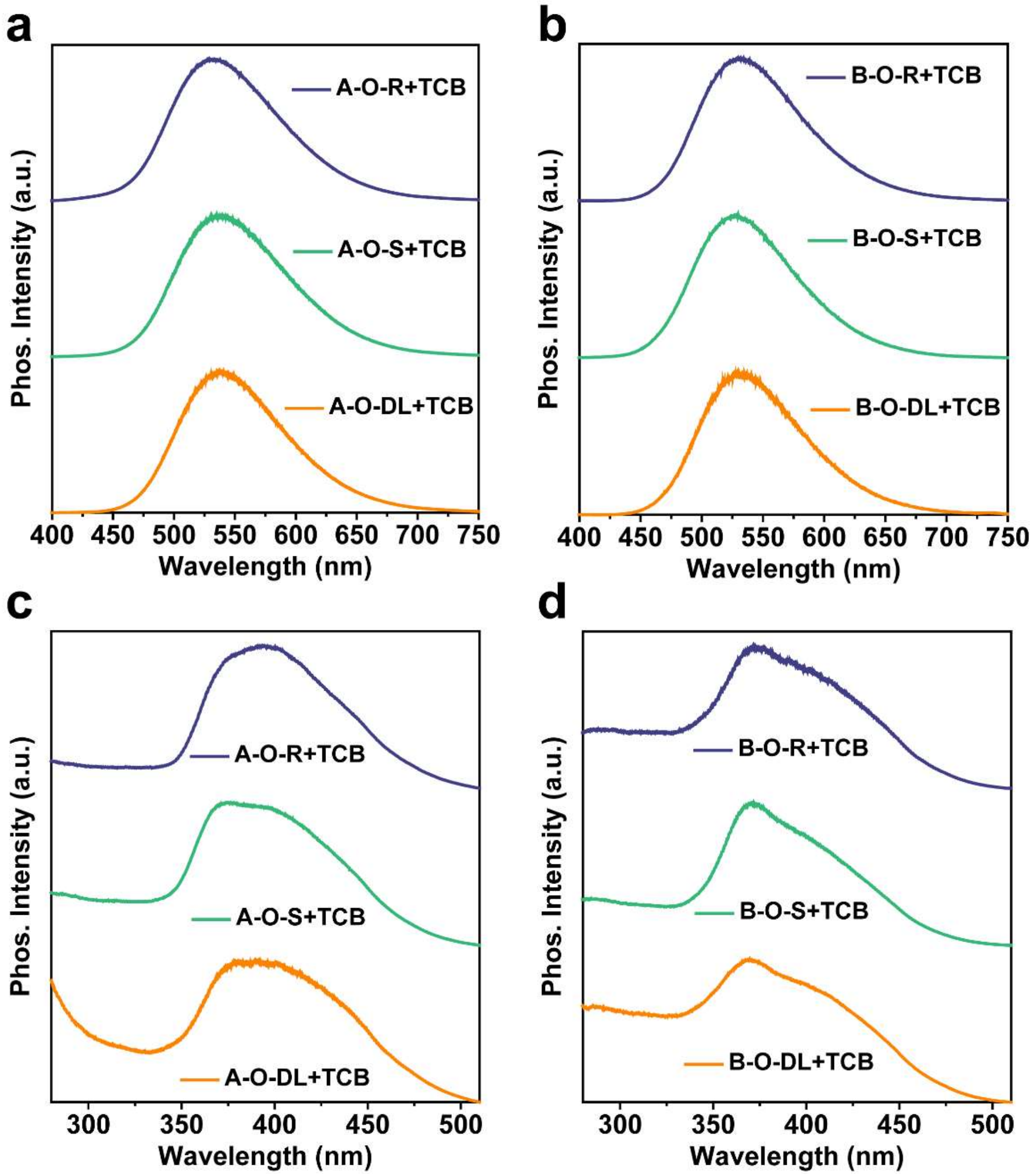

Fig. S67. a) RTP spectra of A-O-S/R/DL+TCB; b) RTP spectra of B-O-S/R/DL+TCB; c) RTP excitation spectra of A-O-S/R/DL+TCB; d) RTP excitation spectra of B-O-S/R/DL+TCB.

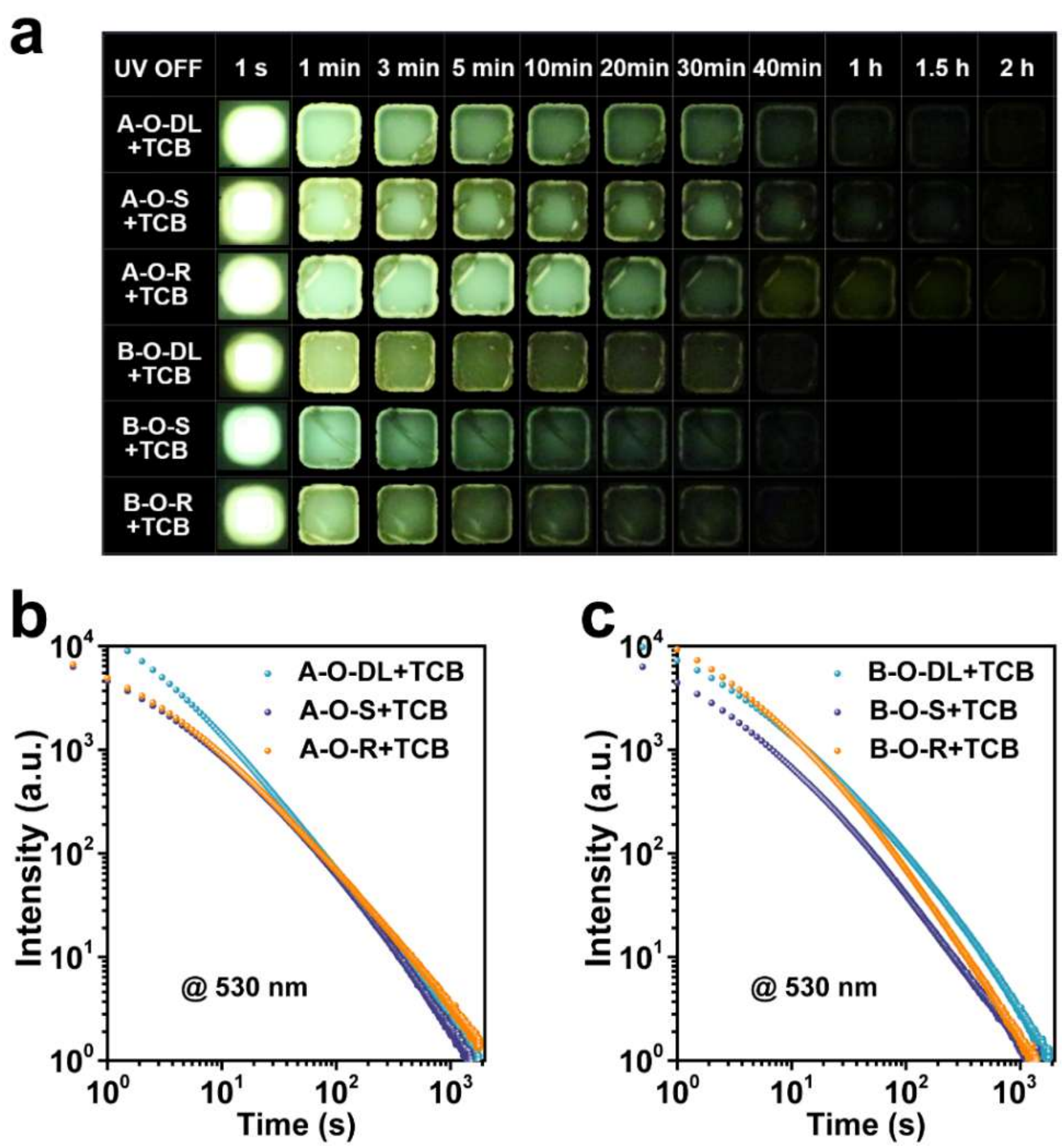

Fig. S68. a) OLPL photographs of A-O-S/R/DL+TCB and B-O-S/R/DL complexes over time. b) Time-resolved OLPL decay curves of A-O-S/R/DL+TCB. c) Time-resolved OLPL decay curves of B-O-S/R/DL+TCB.

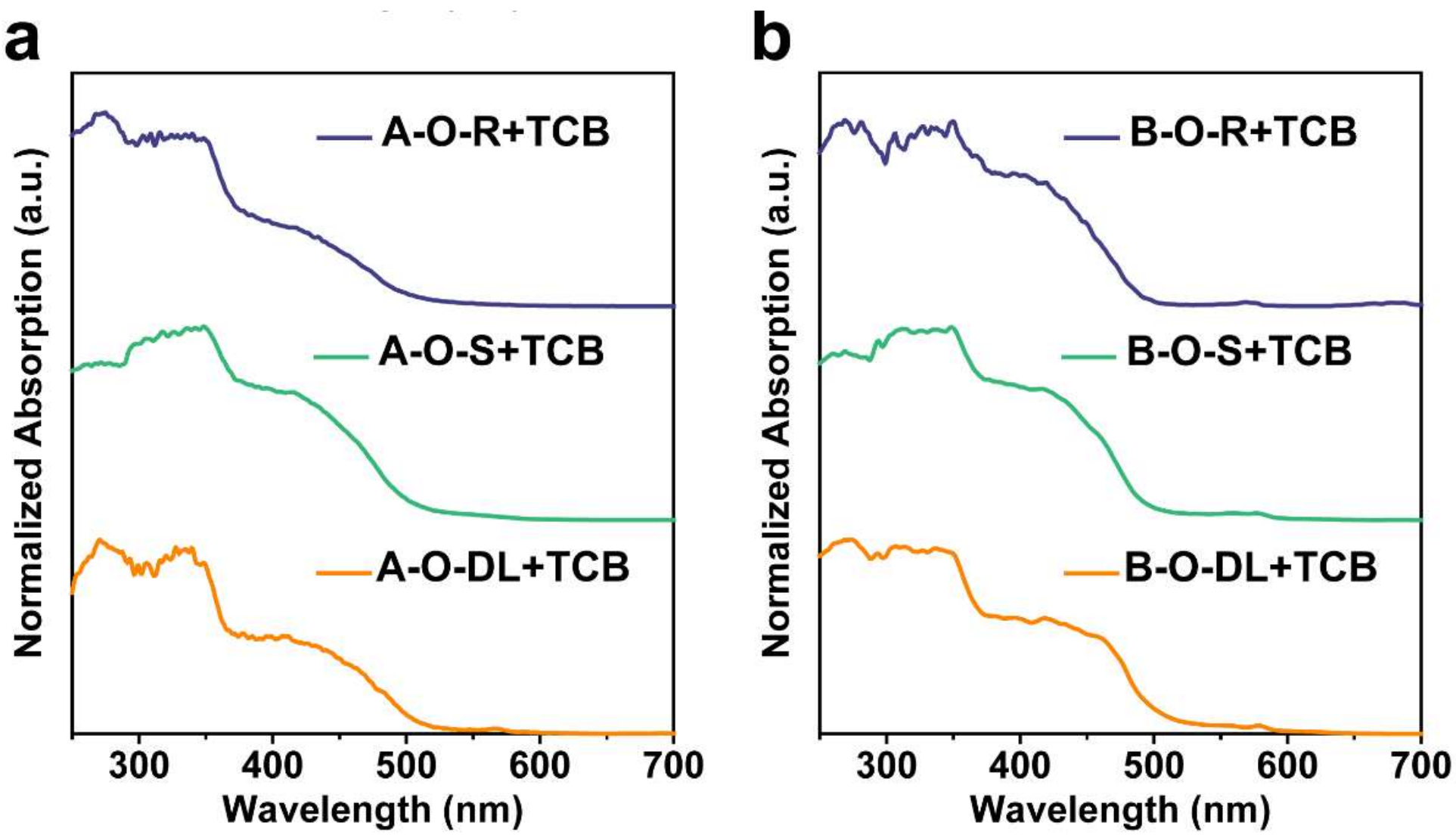

Fig. S69 a) UV-Vis absorption spectra of A-O-R/S/DL+TCB in solid state. (b) UV-Vis absorption spectra of B-O-R/S/DL+TCB in solid state.

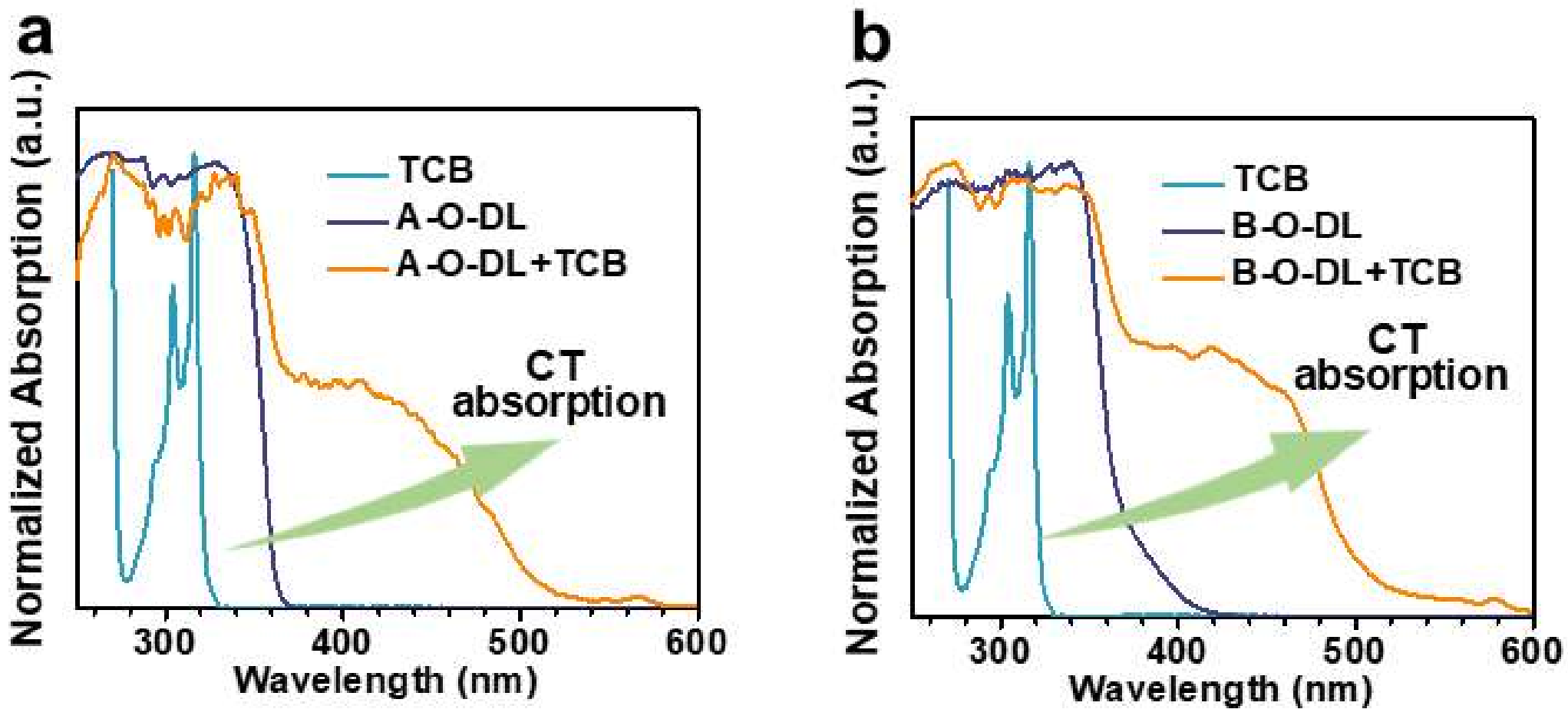

Fig. S70. a) UV-Vis absorption spectra of TCB, A-O-DL and A-O-DL+TCB. b) UV-Vis absorption spectra of TCB, B-O-DL and B-O-DL+TCB.

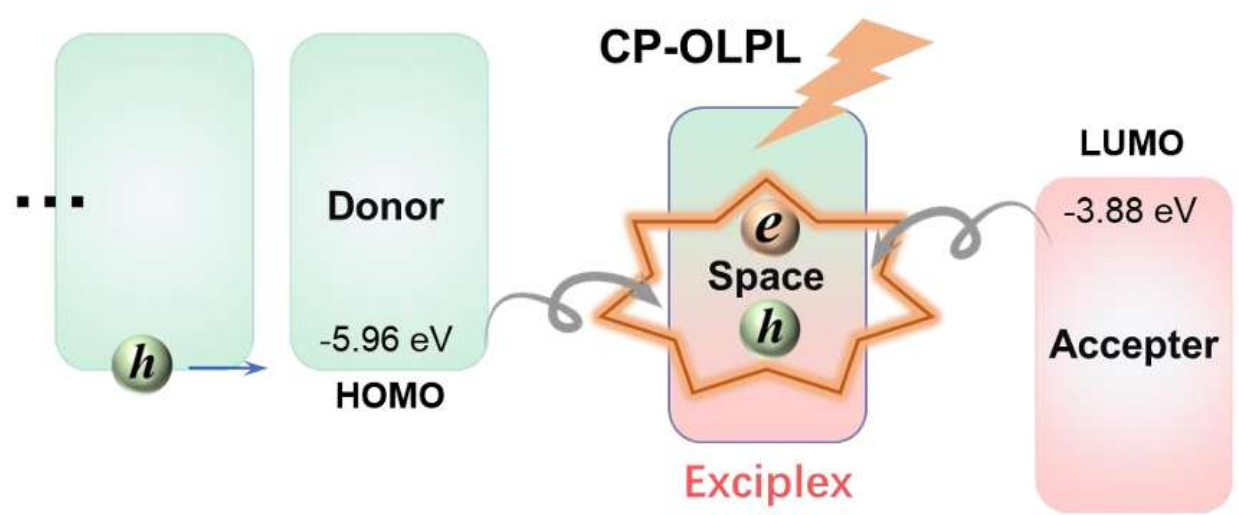

Fig. S71. Circularly polarized organic long persistent luminescence (CP-OLPL) based on charge transfer.

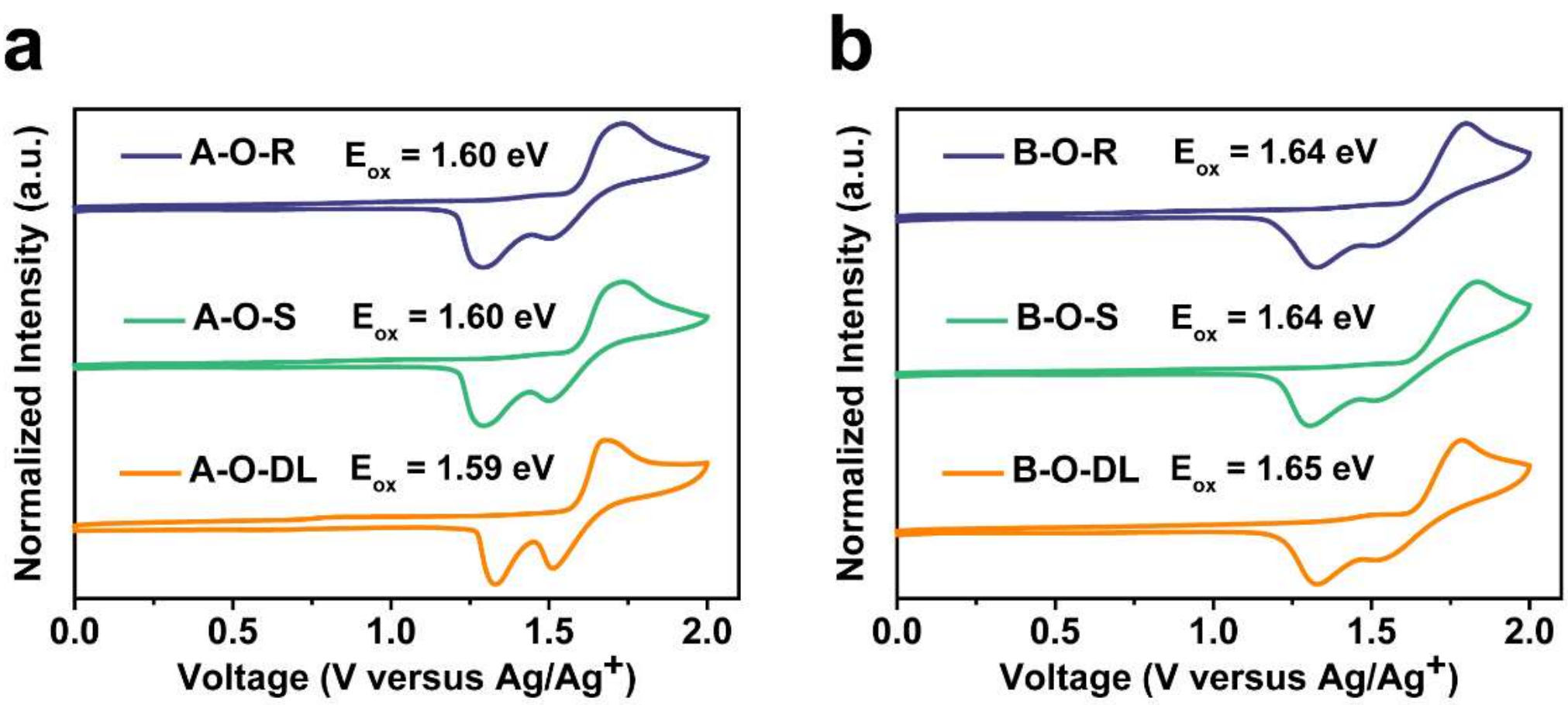

Fig. S72. a) CV curves of A-O-S/R/DL. b) CV curves of B-O-S/R/DL.

Table S6. Energy level data for A-O-S/R/DL and B-O-S/R/DL.

	A-O-S	A-O-R	A-O-DL	B-O-S	B-O-R	B-O-DL
HOMO/eV	-5.95	-5.96	-5.96	-5.01	-5.00	-5.01
LUMO/eV	-2.35	-2.37	-2.36	-1.58	-1.58	-1.58
E_g /eV	3.60	3.59	3.60	3.43	3.42	3.43
E_{ox} /V	1.59	1.60	1.60	0.65	0.64	0.65
λ_{onset}/nm	344.6	345	344.1	361.3	362.1	361.8
$E(_{Fc/Fc^+})$/V	0.44					

Table S7. OLPL data of six kinds of molten mixtures based on different hosts.

	A-O-D+TCB	A-O-S+TCB	A-O-R+TCB	B-O-DL+TCB	B-O-S+TCB	B-O-R+TCB
Φ_{PL}	15.29%	13.29%	12.54%	18.71%	18.87%	18.52%
t(s)	1958.1	1974.9	1960.4	1243.2	1235.5	1245.6

Φ_{PL} = PLQY detected at room temperature;
t(s) = duration time detected at room temperature.

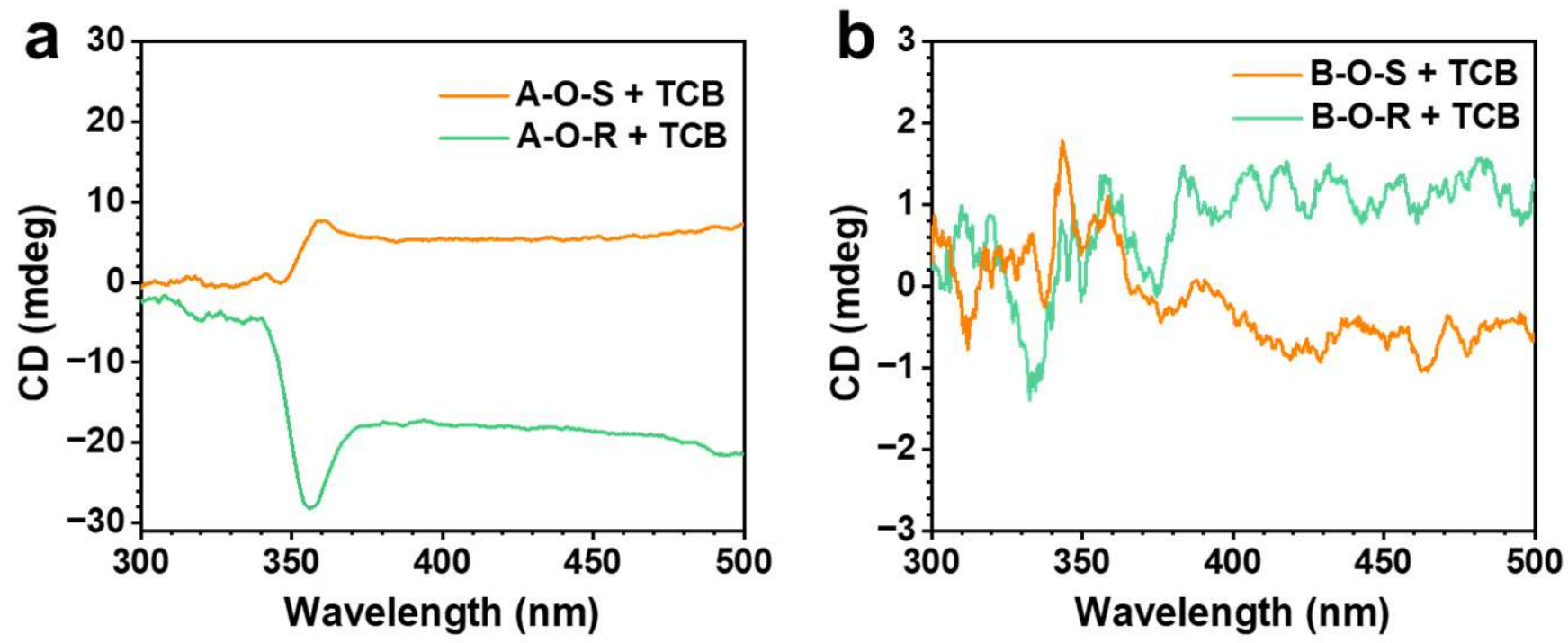

Fig. S73. a) CD spectra of A-O-S/R+TCB. b) CD spectra of B-O-S/R+TCB.

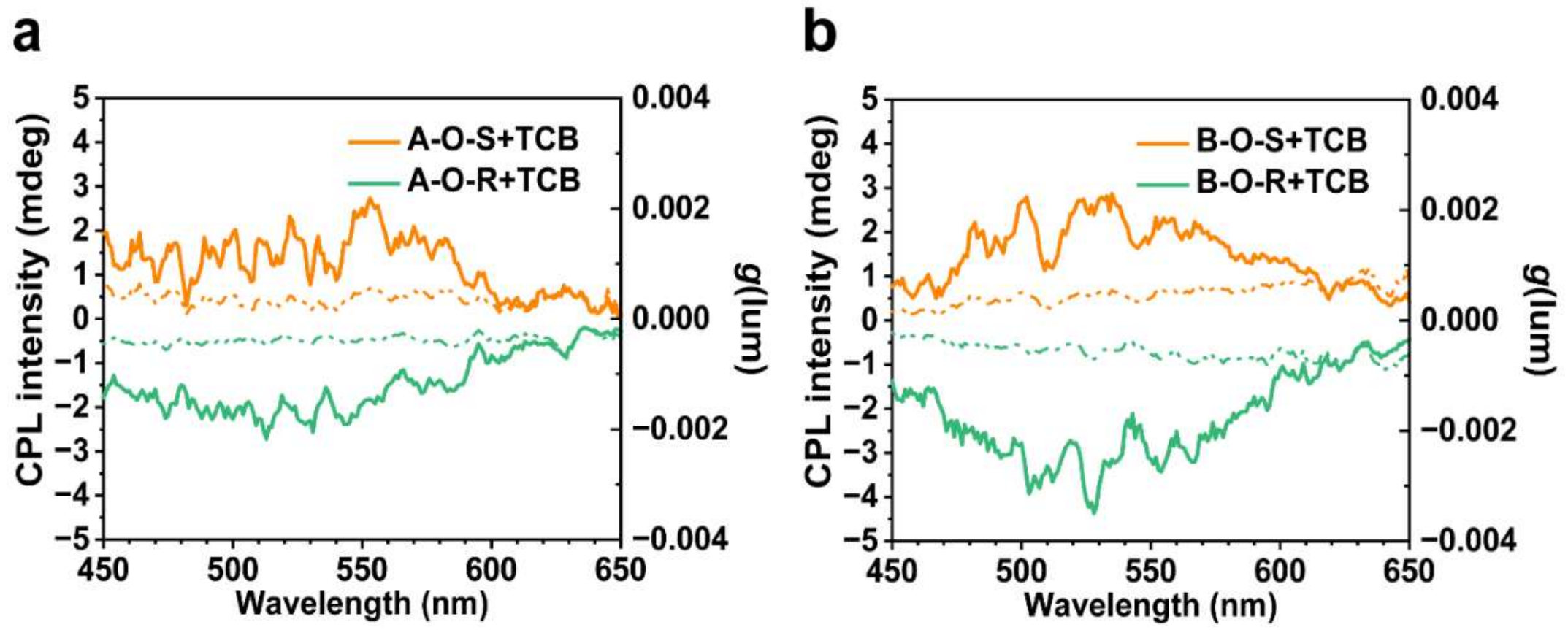

Fig. S74. a) CPL spectra and g_{lum} of A-O-S/R+TCB. b) CPL spectra and g_{lum} of B-O-S/R+TCB.

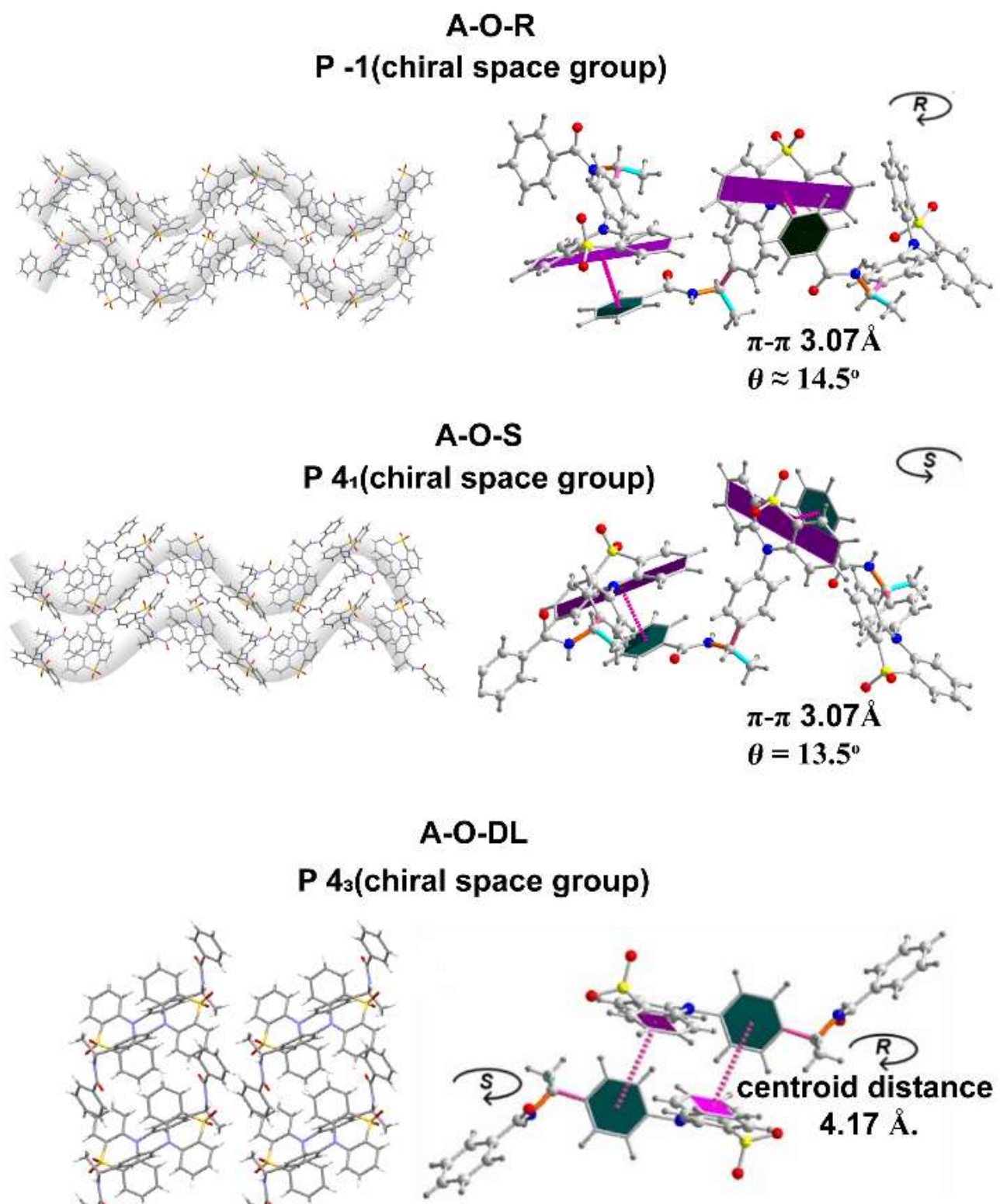

Fig. S75. Crystal structures of A-O-S/R/DL.

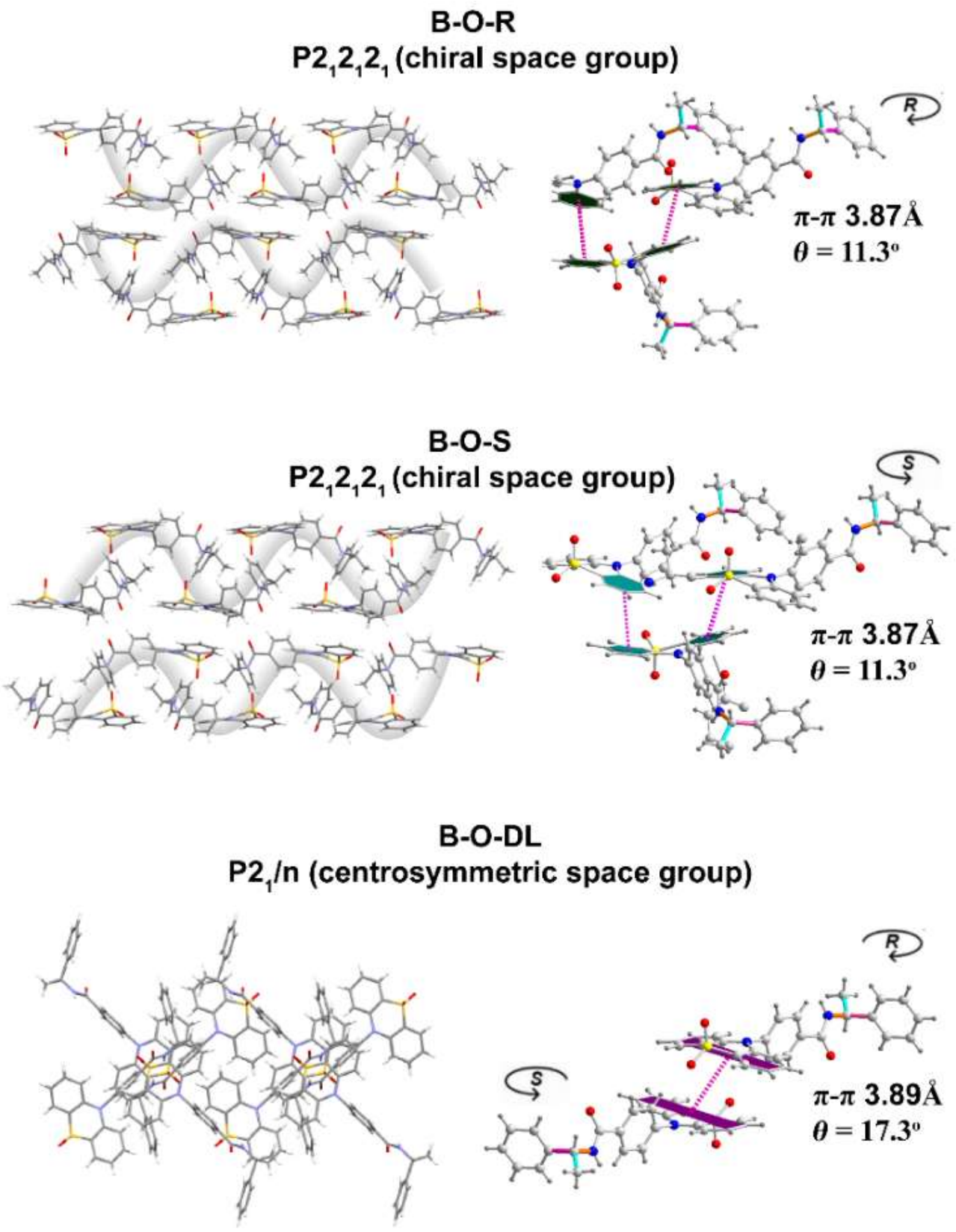

Fig. S76. Crystal structures of B-O-S/R-DL.

Table S8. Single crystal data of A-O-DL, A-O-S, A-O-R, B-O-DL, B-O-S, B-O-R.

	A-O-DL	A-O-S	A-O-R	B-O-DL	B-O-S	B-O-R
Formula	$C_{27}H_{22}N_2O_3S$	$C_{27}H_{22}N_2O_3S$	$C_{27}H_{22}N_2O_3S$	$C_{27}H_{22}N_2O_3S$	$C_{27}H_{22}N_2O_3S$	$C_{27}H_{22}N_2O_3S$
Wavelength (Å)	0.71073	0.71073	0.71073	0.71073	0.71073	0.71073
Space Group	P -1	P 4_1	P 4_3	P 2_1/n	P 2_1 2_1 2_1	P 2_1 2_1 2_1
Cell Lengths (Å)	a=9.1477 (12) b=10.5386 (14) c=12.0501 (16)	a=8.8845 (3) b=8.8845 (3) c=28.2527 (11)	a=8.8784 (3) b=8.8784 (3) c=28.2436 (15)	a=12.5614 (8) b=9.5106 (5) c=18.4350 (12)	a=9.0981 (10) b=12.1380 (16) c=20.349 (3)	a=9.0953 (19) b=12.125 (3) c=20.332 (4)
Cell Angles (°)	α=79.697(5) β=86.723(4) γ=80.779(5)	α=90 β=90 γ=90	α=90 β=90 γ=90	α=90 β=96.976(2) γ=90	α=90 β=90 γ=90	α=90 β=90 γ=90
Cell Volume ($Å^3$)	1127.7(3)	2230.11(17)	2226.33(19)	2186.1(2)	2247.2(5)	2242.2(8)
Z	2	4	4	4	4	4
Density (g/cm^3)	1.339	1.354	1.356	1.381	1.343	1.346
F (000)	476.0	952.0	952.0	952.0	952.0	952.0
h_{max}, k_{max}, l_{max}	11,13,14	11,11,35	11,11,35	15,11,22	11,15,25	11,14,25
CCDC Number	2307971	2307972	2308237	2311016	2308233	2307973

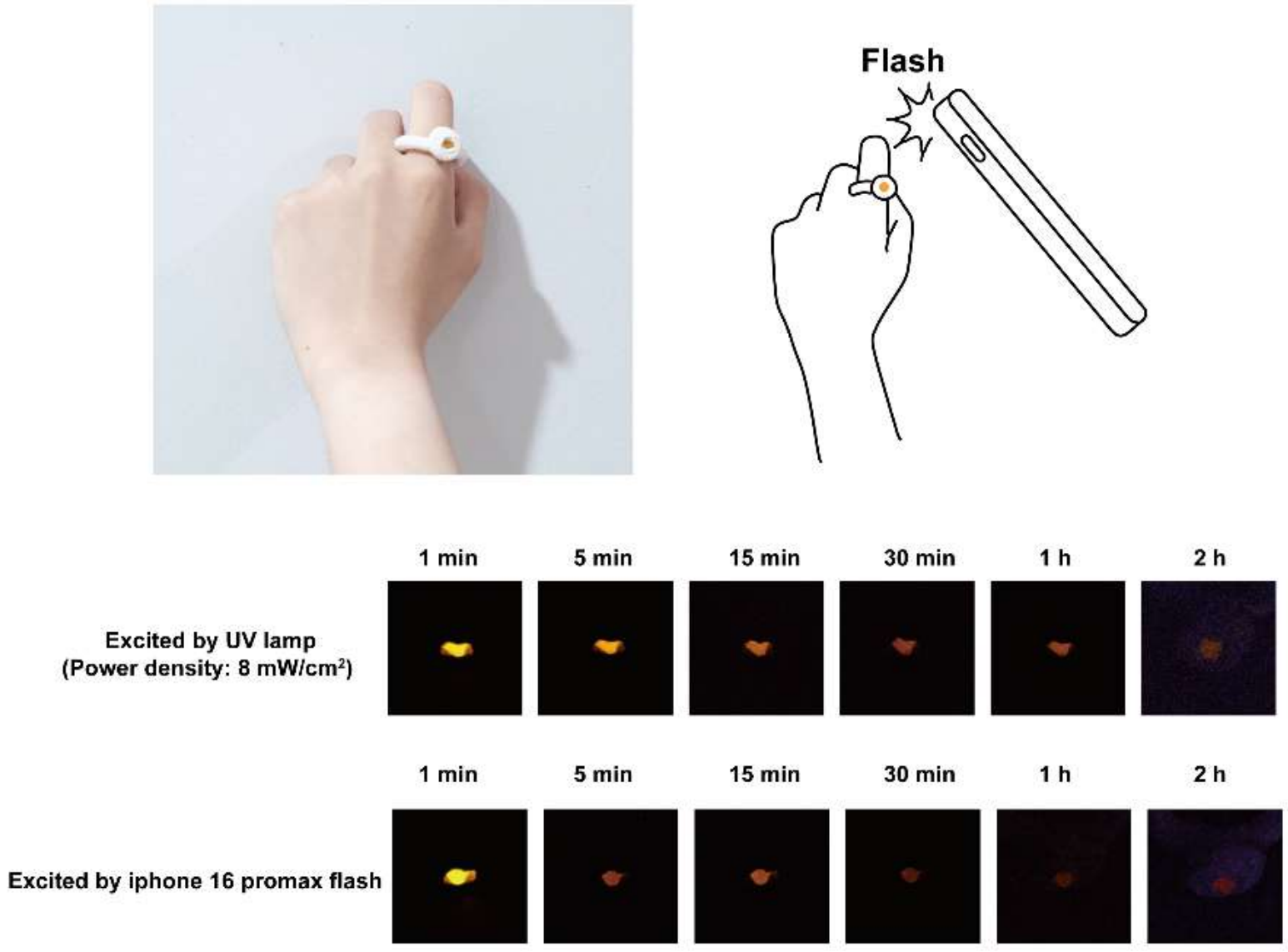

Fig. S77. Demonstration of an OLPL-based ring made by embedding the phosphorescent material into stone-plastic clay.
