# Supplementary material for: A new platform for achieving long-term air-stable long persistent luminescence in amorphous organic system
Source: Light Sci Appl. 2026 Aug 18;15:344. doi: 10.1038/s41377-026-02367-6 (PMC13487193; doi:10.1038/s41377-026-02367-6)

checkCIF/PLATON report

Structure factors have been supplied for datablock(s) A-O-DL

THIS REPORT IS FOR GUIDANCE ONLY. IF USED AS PART OF A REVIEW PROCEDURE FOR PUBLICATION, IT SHOULD NOT REPLACE THE EXPERTISE OF AN EXPERIENCED CRYSTALLOGRAPHIC REFEREE.

No syntax errors found.   CIF dictionary   Interpreting this report

Datablock: A-O-DL

```
Bond precision:   C-C = 0.0032 A           Wavelength=0.71073

Cell:            a=9.1477(12)       b=10.5386(14)     c=12.0501(16)
                 alpha=79.697(5)    beta=86.723(4)    gamma=80.779(5)
Temperature:     296 K

                  Calculated                        Reported
Volume            1127.7(3)                         1127.7(3)
Space group       P -1                              P -1
Hall group        -P 1                              -P 1
Moiety formula    C27 H22 N2 O3 S                   C27 H22 N2 O3 S
Sum formula       C27 H22 N2 O3 S                   C27 H22 N2 O3 S
Mr                454.53                            454.52
Dx,g cm-3         1.339                             1.339
Z                 2                                 2
Mu (mm-1)         0.176                             0.176
F000              476.0                             476.0
F000'             476.44
h,k,lmax          11,13,14                          11,13,14
Nref              4461                              4429
Tmin,Tmax                                           0.543,0.745
Tmin'

Correction method= # Reported T Limits: Tmin=0.543 Tmax=0.745
AbsCorr = MULTI-SCAN

Data completeness= 0.993            Theta(max)= 26.065

R(reflections)= 0.0486( 3643)                      wR2(reflections)=
                                                   0.1239( 4429)
S = 1.105                    Npar= 299
```

The following ALERTS were generated. Each ALERT has the format

test-name_ALERT_alert-type_alert-level.

Click on the hyperlinks for more details of the test.

Alert level C

```
PLAT053_ALERT_1_C Minimum Crystal Dimension Missing (or Error) ...     Please Check
PLAT054_ALERT_1_C Medium  Crystal Dimension Missing (or Error) ...     Please Check
PLAT055_ALERT_1_C Maximum Crystal Dimension Missing (or Error) ...     Please Check
PLAT906_ALERT_3_C Large K Value in the Analysis of Variance ......      2.207 Check
PLAT911_ALERT_3_C Missing FCF Refl Between Thmin & STh/L=    0.600          7 Report
```

Alert level G

```
PLAT007_ALERT_5_G Number of Unrefined Donor-H Atoms ..............          1 Report
PLAT720_ALERT_4_G Number of Unusual/Non-Standard Labels ..........         55 Note
PLAT910_ALERT_3_G Missing # of FCF Reflection(s) Below Theta(Min).          1 Note
PLAT912_ALERT_4_G Missing # of FCF Reflections Above STh/L=  0.600         25 Note
PLAT978_ALERT_2_G Number C-C Bonds with Positive Residual Density.         11 Info
```

0 **ALERT level A** = Most likely a serious problem - resolve or explain
0 **ALERT level B** = A potentially serious problem, consider carefully
5 **ALERT level C** = Check. Ensure it is not caused by an omission or oversight
5 **ALERT level G** = General information/check it is not something unexpected

3 ALERT type 1 CIF construction/syntax error, inconsistent or missing data
1 ALERT type 2 Indicator that the structure model may be wrong or deficient
3 ALERT type 3 Indicator that the structure quality may be low
2 ALERT type 4 Improvement, methodology, query or suggestion
1 ALERT type 5 Informative message, check

It is advisable to attempt to resolve as many as possible of the alerts in all categories. Often the minor alerts point to easily fixed oversights, errors and omissions in your CIF or refinement strategy, so attention to these fine details can be worthwhile. In order to resolve some of the more serious problems it may be necessary to carry out additional measurements or structure refinements. However, the purpose of your study may justify the reported deviations and the more serious of these should normally be commented upon in the discussion or experimental section of a paper or in the "special_details" fields of the CIF. checkCIF was carefully designed to identify outliers and unusual parameters, but every test has its limitations and alerts that are not important in a particular case may appear. Conversely, the absence of alerts does not guarantee there are no aspects of the results needing attention. It is up to the individual to critically assess their own results and, if necessary, seek expert advice.

Publication of your CIF in IUCr journals

A basic structural check has been run on your CIF. These basic checks will be run on all CIFs submitted for publication in IUCr journals (*Acta Crystallographica*, *Journal of Applied Crystallography*, *Journal of Synchrotron Radiation*); however, if you intend to submit to *Acta Crystallographica Section C* or *E* or *IUCrData*, you should make sure that full publication checks are run on the final version of your CIF prior to submission.

Publication of your CIF in other journals

Please refer to the *Notes for Authors* of the relevant journal for any special instructions relating to CIF submission.

PLATON version of 06/07/2023; check.def file version of 30/06/2023

Datablock A-O-DL - ellipsoid plot

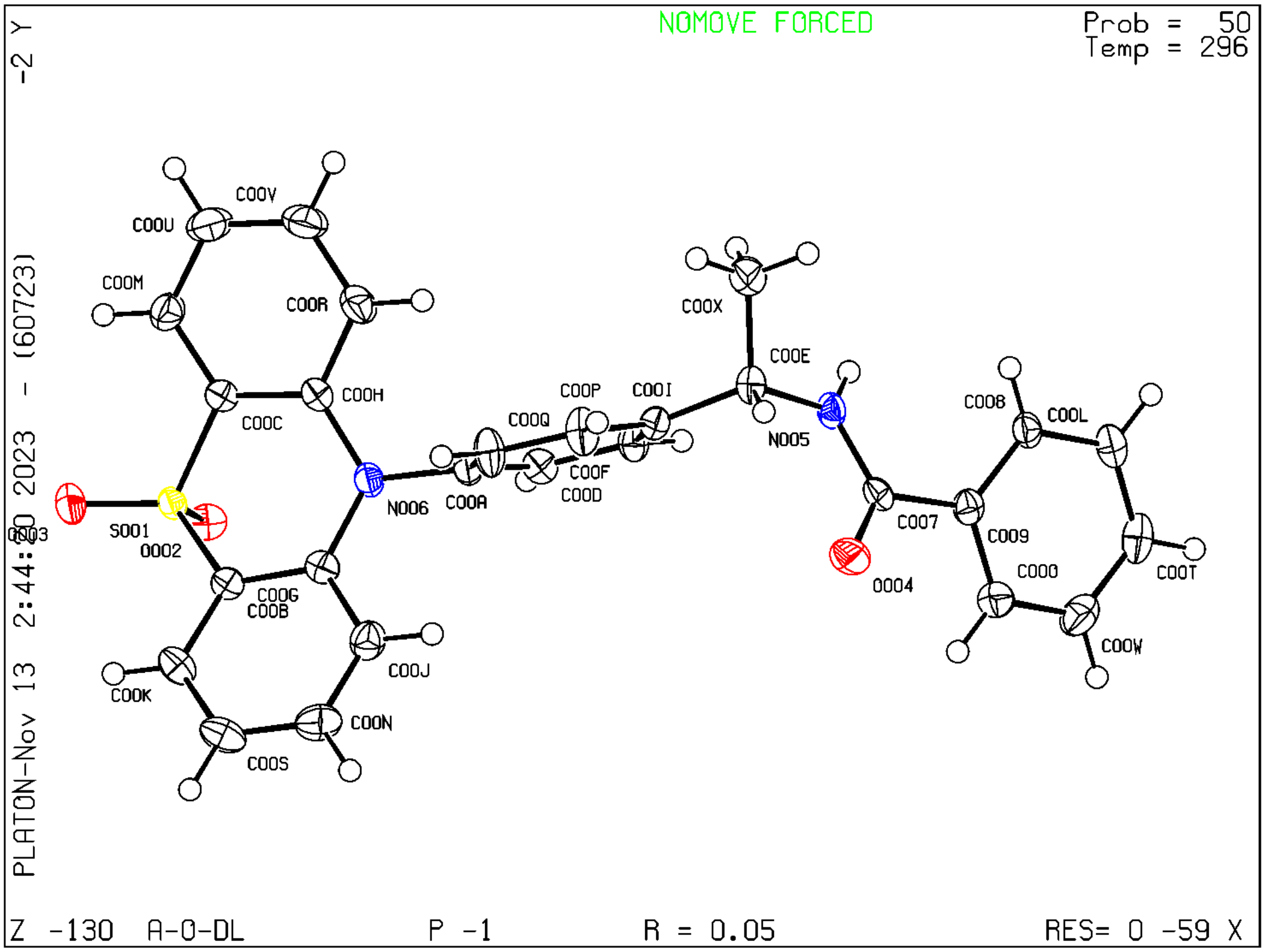

Supplement: Supplementary file 6 [file 41377_2026_2367_MOESM8_ESM.pdf]
